# Identifying geographically differentiated features of Ethopian Nile tilapia (Oreochromis niloticus) morphology with machine learning

**DOI:** 10.1371/journal.pone.0249593

**Published:** 2021-04-15

**Authors:** Wilfried Wöber, Manuel Curto, Papius Tibihika, Paul Meulenbroek, Esayas Alemayehu, Lars Mehnen, Harald Meimberg, Peter Sykacek

**Affiliations:** 1 Institute for Integrative Nature Conservation Research, University of Natural Resources and Life Sciences, Vienna, Austria; 2 Department of Industrial Engineering, University of Applied Science Technikum Wien, Vienna, Austria; 3 Marine and Environmental Sciences Centre, Universidade de Lisboa, Lisboa, Portugal; 4 National Environment Management Authority, Kampala, Uganda; 5 Institute of Hydrobiology and Aquatic Ecosystem Management, University of Natural Resources and Life Sciences, Vienna, Austria; 6 WasserCluster Lunz – Biological Station, Lunz am See, Austria; 7 National Fishery and Aquatic Life Research Center, Sebeta, Ethiopia; 8 Faculty Life Science Engineering, University of Applied Science Technikum Wien, Vienna, Austria; 9 Institute of Computational Biology, University of Natural Resources and Life Sciences, Vienna, Austria; Taipei Medical University, TAIWAN

## Abstract

Visual characteristics are among the most important features for characterizing the phenotype of biological organisms. Color and geometric properties define population phenotype and allow assessing diversity and adaptation to environmental conditions. To analyze geometric properties classical morphometrics relies on biologically relevant landmarks which are manually assigned to digital images. Assigning landmarks is tedious and error prone. Predefined landmarks may in addition miss out on information which is not obvious to the human eye. The machine learning (ML) community has recently proposed new data analysis methods which by uncovering subtle features in images obtain excellent predictive accuracy. Scientific credibility demands however that results are interpretable and hence to mitigate the black-box nature of ML methods. To overcome the black-box nature of ML we apply complementary methods and investigate internal representations with saliency maps to reliably identify location specific characteristics in images of Nile tilapia populations. Analyzing fish images which were sampled from six Ethiopian lakes reveals that deep learning improves on a conventional morphometric analysis in predictive performance. A critical assessment of established saliency maps with a novel significance test reveals however that the improvement is aided by artifacts which have no biological interpretation. More interpretable results are obtained by a Bayesian approach which allows us to identify genuine Nile tilapia body features which differ in dependence of the animals habitat. We find that automatically inferred Nile tilapia body features corroborate and expand the results of a landmark based analysis that the anterior dorsum, the fish belly, the posterior dorsal region and the caudal fin show signs of adaptation to the fish habitat. We may thus conclude that Nile tilapia show habitat specific morphotypes and that a ML analysis allows inferring novel biological knowledge in a reproducible manner.

## Introduction

Visual analysis and use of anatomic features has a long tradition in biology [1–3]. Visual features which are distinct for groups of specimen images provide important information for biological systematics, paleontology, evolutionary, developmental and conservation biology. Classical morphology uses landmarks to define phenotype and modern morphometrics [4] to remove confounding factors that could otherwise impede drawing robust conclusions. Morphometrics is well established [5, 6] and of particular importance in fish biology [7–10]. Fish morphology allows to discriminate genera, species, populations, and even individuals [11]. Morphology allows studying the response of shape to environmental and ecological factors such as trophic behavior [12]. Morphology can furthermore quantify relationships between different species [13, 14] and adaptation of body shape to environmental change [15, 16]. Fish morphology is also known to be an expression of ecological interactions [17].

Landmarks in morphometrics are based on predefined biological knowledge [4, 18]. The advantage of landmarks coming with a biological justification is however paired with the disadvantage of investigations relying on predetermined features. The discovery of novel features of fish anatomy that could be more informative for the question at hand is impossible. Landmark based analyses have the additional disadvantage of being quite laborious. Every sample needs a careful characterization by placing landmarks in images before further analyses are possible. Attempts to replace landmarks with automatically derived sample characteristics are thus on the agenda of many scientific laboratories. Using Machine learning (ML) for this task depends on a collection of labeled images which are used as training data. To identify location specific characteristics in Nile tilapia morphology we will simultaneously learn to distinguish sample categories and assess the relevance of input features.

Classification can be approached with fully generative models [19, 20] or by diagnostic models which express class labels or their probabilities as function of input features [21]. An intermediate approach between fully generative and diagnostic classification was recently proposed in [22] where transitions between prototypical samples are represented as one dimensional manifolds and classification assesses test cases on the basis of calculated distances. Approaches like [19, 20, 22] which represent input data density or aspects thereof are advantageous if missing data is a problem or in situations which require detection of outliers. To identify visual features in specimen images which are important to discriminate between labels this work relies on established practice and combines diagnostic models with an identification of feature relevance [45]. Recent success of deep learning [23] where in particular convolutional neuronal networks (CNNs) dominated all recent image classification contests since 2012 suggests to consider methods like [24] to analyze the Nile tilapia images. Deep learning refers to a class of methods which descend from neural networks, where many layers of neurons are concatenated. CNNs [25, 26] use deep architectures which are very useful for image processing. In a biological context CNNs have been used to classify plants [27, 28] and fish [29–34].

While technical aspects like good classification accuracy are a necessary prerequisite, inferring reproducible biological knowledge depends in addition on critical assessments of how we arrive at predictions. The latter consideration is of particular importance when relying on CNNs and other powerful approaches. Subtle dependencies between input and output variables inform predictions irrespective of whether extracted features represent meaningful biology or might be caused by artifacts which show an unfortunate confounding with the analysis goal. This paper hypothesizes that a robust and transparency enforcing machine learning based analysis will help to draw reproducible conclusions. We evaluate this hypothesis against an alternative approach which relies on a naïve analysis with CNNs. Striving for robust conclusions is motivated by the no free lunch theorems [35] which prove that the unknown characteristics of a data set are linked with how well different approaches cope with an analysis challenge. Looking for insight about how predictions come about is rooted in recent observations (e.g. [36]) that excellent predictive performance may be aided by artifacts in the data which are correlated with the class label. According to [37] it is thus imperative to safeguard that predictions are a result of biologically plausible features. By highlighting image regions which carry discriminatory information of a sample, transparency enforcing mechanisms will also help to elucidate which Nile tilapia body characteristics are location specific.

The data which we use to evaluate our hypothesis consists of 209 Nile tilapia images which were sampled from the six different Ethiopian lakes which we list in Table 1.

**Table 1:** Six Ethiopian lakes which constitute the origin of 209 Nile tilapia specimen.

Lake name	Coordinates	Elevation [*m*]	Area [*km*^2^]	Nr. samples
Chamo	*N*5°5′0 *E*37°33′0	1110	317	36
Hawassa	*N*7°3′0 *E*38°26′0	1686	129	38
Koka	*N*8°23′31.1 *E*39°4′36.45	1595	180	31
Langano	*N*7°37′0 *E*38°46′0	1585	230	26
Tana	*N*12°1′0 *E*37°17′31	1788	3060	38
Ziway	*N*8°0′3 *E*38°49′16	1636	440	40

This table lists the six Ethiopian lakes which were visited for sample collection. The lakes are characterized by geographical coordinates, elevation above sea level, area and the number of Nile tilapia sample images which were collected at every site.

By learning to predict the known origin of fish images, data analysis will extract image characteristics which are specific for every lake. If we may assume that the extracted features represent valid aspects of fish morphology, good predictive accuracy will facilitate investigations about specialization and population differentiation. Such analysis is in principle suitable to relate species [13, 14] or to evaluate intraspecific variation [15, 16]. To obtain robust and reproducible verdicts about variation of Nile tilapia morphology we analyze this data with complementary data analysis methods.

- ML based feature extraction with CNNs [25, 26] and Bayesian Gaussian process latent variable models (GP-LVM) [38] are compared against manually assigned landmarks of fish morphology which underwent a generalized Procrustes analysis (GPA) [39–41].
- To infer classifiers which predict the origin of samples we applied Gaussian process classifiers (GPC) [42, 43] and a Bayesian multi-layer perceptron (HMC-MLP) which was inferred with hybrid Monte Carlo sampling [44, 45]. Since CNNs are classifiers with built in feature extractors, CNNs constitute a third approach which classifies fish images by sample origin.
- We rely on generalization accuracy (Acc), mutual information (MI) and McNemars significance test (Sig) to obtain complementary assessments of classification performance [46]. For judging reproducibility and to guarantee unbiased assessments we repeat a ten fold cross testing procedure [42, 46, 47] ten times on reshuffled data sets.
- To assess which image features aid CNN predictions, we apply backward propagation of relevance (LPR) [48] and Grad-CAM [49] to individual samples. LPR and Grad-CAM based saliency maps are used to identify regions in sample images which are influential for the calculated predictions. GP-LVM provides on the scale of the entire data set insight into how different image regions contribute to the learned representation. Using automatic relevance determination (ARD) during GPC and HMC-MLP inference fosters identifying Nile tilapia body characteristics that are specific to the animals habitat. To mitigate the human factor in interpreting saliency maps we devise a novel statistical test and use a p-value cutoff of 0.001 to mark important image regions in a reproducible manner.

**Fig 1:**
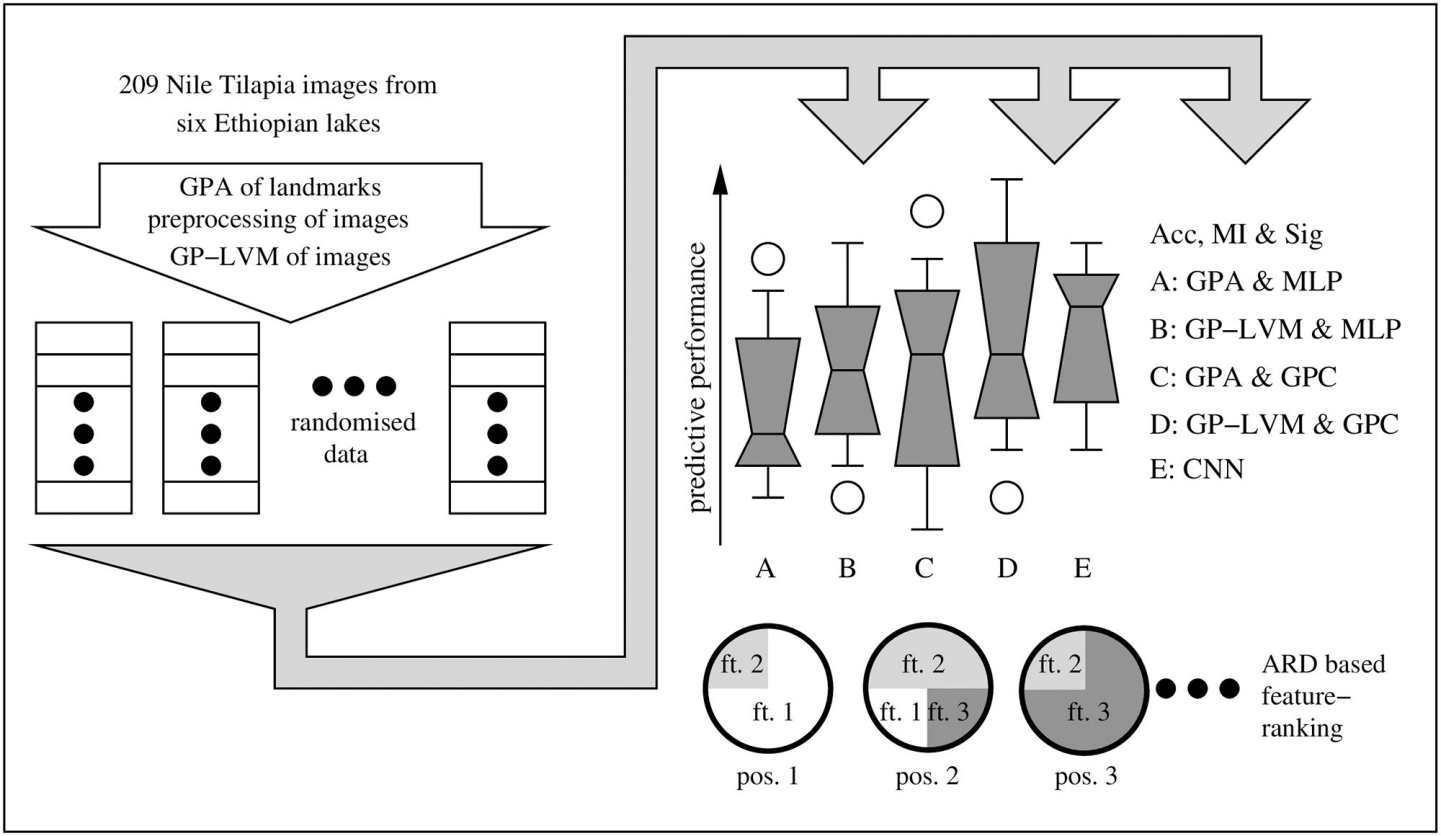
Data analysis of Nile tilapia images. The objective of our approach is to provide robust conclusions by randomly reshuffling the original data and repeating analysis ten times. The input features considered in our analysis are 1) established landmarks of fish morphology which underwent a generalized Procrustes analysis (GPA); 2) Gaussian process latent variable representations of fish images (GP-LVM) and 3) features extracted by deep convolutional neural networks (CNN). Nile tilapia origin is classified from GPA and selected GP-LVM features with Gaussian process classifiers (GPC) and Bayesian multi layer perceptrons which were inferred with a hybrid Monte Carlo algorithm (MLP). Combining feature extraction and classification, the CNN is directly applied to Nile tilapia images. Unbiased predictions were obtained with ten fold cross testing and assessed by generalization accuracy (Acc) and mutual information between features and labels (MI). McNemars test was used to assess the differences between the least performing classifier and all improved results for statistical significance (Sig). ARD level based feature ranks allow us to reason which visual features of Nile tilapia adapt to habitat.

The remaining part of the paper provides a detailed description of a Nile tilapia data set and the data analysis pipelines which we illustrate in Fig 1. We use the term *pipeline* to denote a cascade of compatible data analysis stages. The emphasis of the rather involved data analysis strategy is to obtain reproducible conclusions about habitat specific characteristics in Nile tilapia images which may have some adaptation value. The results section applies all analysis methods in practice. We provide an assessment of different analysis methods, investigate robustness of preferences and provide insight into inferred models. Careful model checking identifies and removes technical artifacts and provides reproducible biological conclusions that Nile tilapia morphology shows population specific variation. The paper closes with a conclusion where we evaluate our hypothesis and provide a short guideline on how to apply ML to arrive at biologically meaningful findings.

## Materials and methods

Evaluating whether Nile tilapia has population specific morphotypes in a biologically robust manner depends on a comprehensively annotated data set and the elaborate data analysis which we discuss in the following subsections of the paper.

### Data

#### Ethical and legal considerations

Nile tilapia is not a protected species, but rather fished commercially. Sampling was conducted in collaboration with respective local authorities and therefore no special permission was required. All Nile tilapia samples which went into this investigation where harvested by local fishermen and used for commercial purposes. In all cases, the fish were already dead when obtained from the fishermen. For obtaining the Nile tilapia images a special treatment of the animals was therefore not required.

#### Data collection

**Fig 2:**
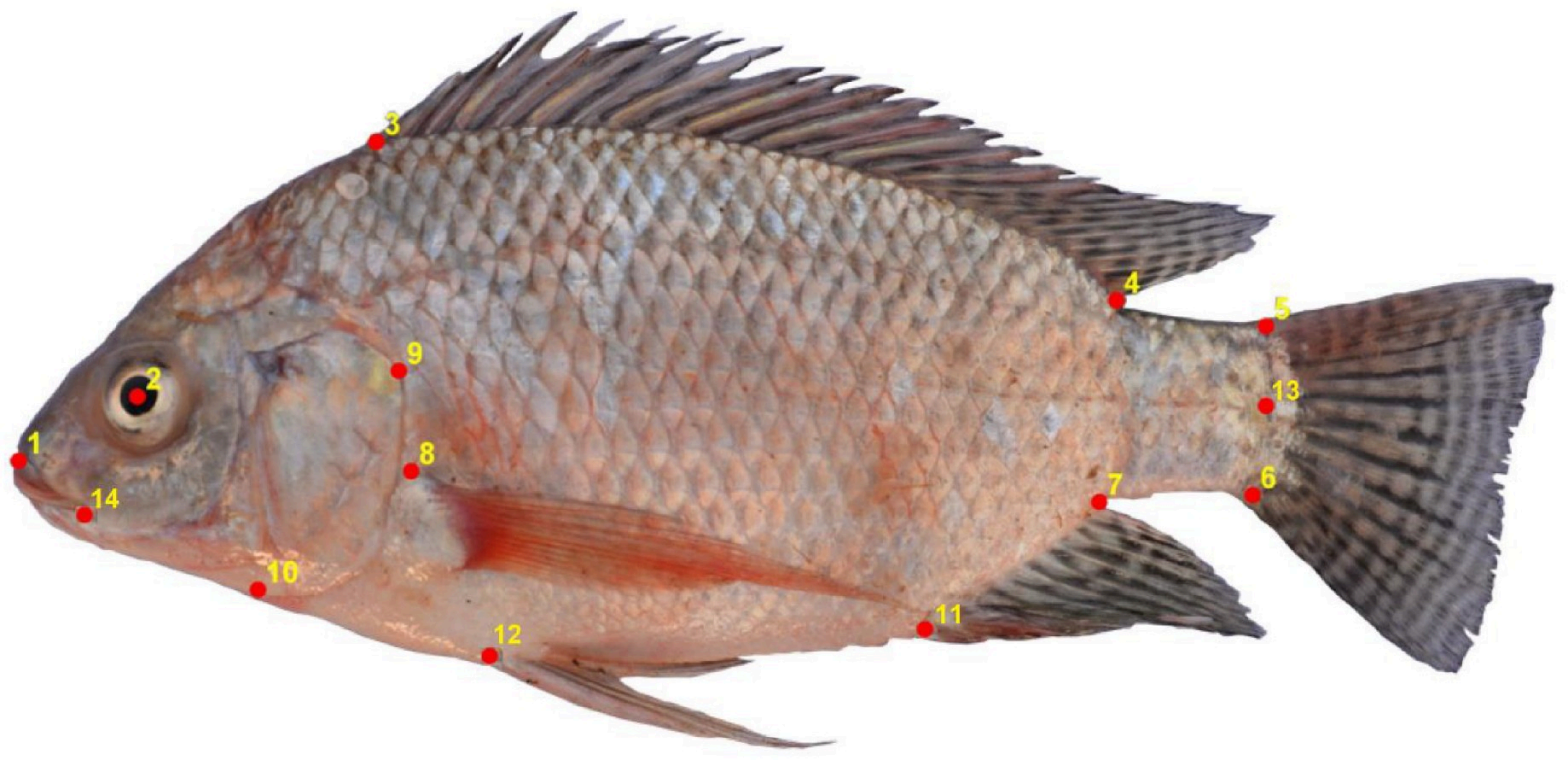
Landmark positions on an image of a Nile tilapia fish. A classical morphometrics analysis of Ethiopian Nile tilapia is based on the 14 landmark positions which we illustrate in this image of a Nile tilapia fish.

To provide conclusions which are relevant for typical field work, the data which we consider in this study consists of 209 Nile tilapia fish images that were collected at six different Ethiopian lakes under natural conditions. An overview of the locations and the number of samples is found in Table 1. To maintain identical orientation all Nile tilapia samples were photographed from the left side with a Nikon D5200 digital camera. Images were taken with a 18mm lens at constant focal length of 30cm and labeled according to the lake of origin. To compare purely ML based analysis strategies with classical morphometrics, all fish images were annotated with the 14 two dimensional landmarks that were previously used by [50]. The landmarks are depicted in Fig 2 and further described in Table 2. The data which we use in this study consists thus of 209 images and a 209 × 28 dimensional matrix with landmark coordinates. Images and landmarks were processed and subsequently used as input to infer classifiers which learn to predict the respective lake of origin. We are however not interested in the predictions per se. The objective of a biologically meaningful analysis lies in 1) identifying whether and how much information Nile tilapia body characteristics contain about the lake of origin and 2) an identification of Nile tilapia body characteristics which allow to distinguish the origin of samples. To deduce this information we apply the analysis pipelines which are shown in Fig 1.

**Table 2:** Characterization of the 14 landmark positions in Fig 2.

Nr.	Landmark name	acronym
1	Upper tip of snout	UTP
2	Center of eye	EYE
3	Anterior insertion of dorsal fin	AOD
4	Posterior insertion of dorsal fin	POD
5	Dorsal insertion of caudal fin	DIC
6	Ventral insertion of caudal fin	VOC
7	Posterior insertion of anal fin	PIA
8	Dorsal base of pectoral fin	BPF
9	Most posterior edge of operculum	PEO
10	Ventral edge of operculum	VEO
11	Anterior insertion of anal fin	AOA
12	Anterior insertion of pelvic fin	AOP
13	Halfway between dorsal and ventral insertion of caudal fin	HCF
14	Posterior end of mouth	EMO

This table relates the landmark numbering of Fig 2 to a textual description and an acronym which we use throughout the paper for referring to different landmarks. These landmarks are used to characterize 209 Nile tilapia specimen for a classical morphometrics analysis.

### Data analysis

Our decision to apply complementary methods is motivated by established knowledge that models have an impact on analysis results [35, 51]. The ability of modern ML methods to discover subtle features in data may impede inferring reproducible biological knowledge, if technical artifacts happen to contain information about class labels [36]. To approach both challenges we apply the data analysis pipeline in Fig 1. The coordinates of the 14 landmarks in Table 2 are transformed by a generalized Procrustes analysis (GPA) [41] to yield GPA-shape features. The Nile tilapia images are also used directly as inputs of a Gaussian process latent variable model (GP-LVM) [38] and for analysis with convolutional neural networks (CNNs) [25, 26].

To prepare analysis all images are converted to gray scale and resized such that all fish have the same length. An important consideration in image processing is deciding on a spatial resolution which is adequate for the task. While undersized resolutions bear the risk of information loss, too large resolution will pick up noise which will again deteriorate performance. We decided to follow the choice reported in [24] for their CNN implementation which ranked highly successful in the ImageNet 2014 challenge and converted all Nile tilapia images to a resolution of 224 × 224 pixels. The added value of using 224 × 224 pixel images is that the applied CNN can be initialized with the pre-trained ImageNet [52] model of VGG-16 which comes with Keras [53, 54]. Nile tilapia images at a resolution of 224 × 224 pixels are also the input to feature extraction with GP-LVM. To quantify model uncertainty the analysis pipeline in Fig 1 reshuffles the 209 Nile tilapia samples and repeats all analyses ten times. To obtain paired predictions, all data analysis pipelines use the fish specimen in the same order.

The proposed analysis classifies GPA-shape and GP-LVM features with Gaussian process classifiers [42, 43] (GPC) and a multi layer perceptron which we infer with R. Neals Hybrid Monte Carlo approach [44, 45] (HMC-MLP). Both classifiers allow for automatic relevance determination (ARD) and thus to assess the importance of input features. Providing performance assessments and feature rank metrics allows us to infer whether Nile tilapia adapts to habitat and which body parts change. CNNs [25, 26] map images directly to probabilities of class and constitute a fifth approach for classifying Nile Tilapia with respect to lake of origin. Ten fold cross testing [47] provides unbiased performance assessments on all reshuffled data sets.

Complementary views at predictive performance are obtained by calculating generalization accuracy (Acc), mutual information between inputs and outputs (MI) and pairwise McNemar significance levels (Sig) when comparing the least performing classifier against all other predictors. Training on reshuffled data will lead to model inference finding different local optima and hence capture the variation in performance of different pipelines. The variability in Acc, MI and Sig which results from data reshuffling is visualized as box plots. Randomization also captures the uncertainty about feature relevance for predicting the origin of Nile tilapia samples. The resulting rank uncertainty is visualized as pie charts where the feature distribution at leading rank positions is represented as pie segments. While large generalization accuracy and mutual information of image based predictions would hint lake specific phenotypes, the biological relevance of such findings is still far fetched. To draw reproducible conclusions, we have to investigate the nature of patterns and image regions which provide information about the lake of origin. The trained CNNs are to this end analyzed with backward propagation of relevance [48] and Grad-CAM [49]. The generative nature of GP-LVM allows for a direct mapping of discriminatory features to image space. After this broad overview of the proposed data analysis we will discuss important methodological details in the subsequent sections.

#### Landmarks and GPA

To remove effects from landmarks like differences in sample size or orientation, GPA was performed using function *procGPA()* from the R package *shapes*, [41]. To discuss GPA briefly we follow [39–41] and represent landmark *m* ∈ [1, *M*] of Nile tilapia sample *n* ∈ [1, *N*] as two dimensional column vector $\psi_{n,m}=[x_{n,m},y_{n_{m}}{]}^{T}$. All landmarks of sample *n* can thus be represented as [2 × *M*] dimensional matrix ***Y***_*n*_ = (***ψ***_*n*,1_, …, ***ψ***_*n*,*M*_). The goal of the GPA is to convert all *N* initial configurations ***Y***_*n*_ to shapes. GPA adjusts to this end all configurations to obtain identical location and scale. A subsequent correction for rotational variation completes GPA. GPA is thus a three step procedure.

1. The initial configurations ***Y***_*n*_ are translated such that all sample centroids *c*(***Y***_*n*_) = 1/*M*∑_*m*_ ***ψ***_*n*,*m*_ coincide with the origin of the coordinate system.
2. The translated configurations are rescaled such that configuration ***Y***_*n*_ gets a unit centroid size. Using ***I***_*M*_ as *M*-dimensional identity matrix the centroid size is defined as $S(Y_{n})=\sqrt{\text{trace}(Y_{n}^{T}C^{T}CY_{n})}$ where $C=I_{M}-1/M1_{M}1_{M}^{T}$ denotes the centering matrix.
3. The final GPA shapes, ***Z***_*n*_, are obtained by rotation such that the agreement between ***Z***_*n*_ and the mean shape ***μ***_*Z*_ = 1/*N*∑_*n*_ ***Z***_*n*_ is optimized. Optimization minimizes to this end for every shape trace(***μ***_*Z*_***Z***_*n*_**Γ**_*n*_) w.r.t. **Γ**_*n*_ ∈ *Sr*(*R*^2^) where *Sr*(*R*^2^) denotes the set of all rotation matrices of the 2 dimensional Euclidean space.

The resulting set of shapes *Z* = [***Z***_1_, .., ***Z***_*N*_] characterize Nile tilapia landmarks invariant to differences in location, size and angular rotation of the fish in the image. To fit to the analysis which is shown in Fig 1 we convert *Z* to a [*N* × 2*M*] dimensional matrix ***X*** where column ***X***[:, 2*m* − 1] denotes all *x* and column ***X***[:, 2*m*] all *y* coordinates of the *m*-th GPA-shape feature.

#### Gaussian process latent variable models

To compare the landmark based analysis with analyses which rely entirely on ML methods, we extract features from Nile tilapia images with Gaussian process latent variable models (GP-LVM) [55, 56]. GP-LVM is an unsupervised ML method which represents an [*N* × *D*] dimensional data matrix ***Y*** by a [*N* × *L*] dimensional matrix ***X*** and a probabilistic model *p*(***Y***|***X***, ***θ***). We use *N* to denote the number of samples, *D* as dimension in observation space, *L* ≪ *D* as latent variable dimension and ***θ*** to denote the GP-LVM parameters.

GP-LVM models the *D* columns of ***Y***, ***y***_*d*_, independently as Gaussian process. We will to this end adopt the notation

$$\begin{array}{ccc} p(y_{d}|f_{d}) & = & {\left(2\pi \right)}^{-N/2}\lambda^{N/2}\exp\left(-0.5\lambda {\left(y_{d}-f_{d}\right)}^{T}I\left(y_{d}-f_{d}\right)\right),\\ p(f_{d}|X,\theta ) & = & {\left(2\pi \right)}^{-\frac{N}{2}}{\left|K_{f,f}\left(X,\theta \right)\right|}^{-\frac{1}{2}}\exp(-\frac{1}{2}f_{d}^{T}K_{f,f}{\left(X,\theta \right)}^{-1}f_{d})\\ \text{and}\;\text{thus} & & \\ p(Y|X,\theta ) & = & {\left(2\pi \right)}^{-\frac{ND}{2}}{\left|K_{f,f}\left(X,\theta \right)+\lambda^{-1}I\right|}^{-\frac{D}{2}}\\ & \cdot & \prod_{d=1}^{D}\exp(-0.5y_{d}^{T}{\left(K_{f,f}\left(X,\theta \right)+\lambda^{-1}I\right)}^{-1}y_{d}) \end{array}$$ (1)

All data columns, ***y***_*d*_, are hence considered observations of a zero mean Gaussian process with an additive Gaussian noise term with variance λ^−1^. The vector ***f***_*d*_ in Eq (1) represents the noiseless signal in column *d*. The Gaussian process (GP) *p*(***f***_*d*_|***X***) is governed by an [*N* × *N*] dimensional covariance matrix, ***K***_*f*,*f*_(***X***, ***θ***). The covariance matrix is a deterministic function of ***X*** and ***θ*** and in GP-LVM assumed to be identical for all data columns. GP-LVM learns thus to represent all columns of ***Y*** with the same latent variables ***X***. GP-LVM is parameterized via a covariance function ***K***_*f*,*f*_[*n*, *ν*](***X***, ***θ***) = *k*(***X***[*n*,:], ***X***[*ν*,:], ***θ***) where ***X***[*n*,:] and ***X***[*ν*,:] denote rows *n* and *ν* of ***X***. The vector ***θ*** represents the parameters of the covariance function. Due to the large number of parameters and many local optima, determining optimal values for ***X*** and ***θ*** by maximizing the likelihood *p*(***Y***|***X***, ***θ***) is cumbersome and has a tendency to overfit [57, 58].

Current applications of GP-LVM rely thus on a later extension by [38] which combines variational ideas and sparse GPs [58, 59] to reduce the number of parameters considerably. Denoting the latent representation of sample *n* as ***X***[*n*,:], [38] acknowledge that ***X*** is a [*N* × *L*] matrix of random variables and specify a factorizing prior $p(X)=\prod_{n=1}^{N}N(X[n,:]|0,I)$. Approximating the true posterior *p*(***Y***|***X***) as $Q(X)=\prod_{n=1}^{N}N(X[n,:]|\mu_{n},S_{n})$ allows the authors of [38] to formulate a variational lower bound *F*(*Q*) < log(*p*(***Y***))

$$\begin{array}{ccc} F(Q) & = & \int_{X}Q\left(X\right)\log(\frac{p(X)p(Y|X)}{Q(X)})dX\\ & = & \tilde{F}\left(Q\right)-\text{KL}\left(Q\left(X\right)||p\left(X\right)\right). \end{array}$$ (2)

While the Kullback Leibler (KL) divergence *KL*(*Q*(***X***)||*p*(***X***) is analytically tractable, nonlinear interactions between ***X*** and ***Y*** require approximating $\tilde{F}(Q)$ further. The suggestion in [38] to use a sparse GP for *p*(***f***_*d*_|***X***) is achieved by augmenting the model for every dimension *d* by *M* < *N* inducing variables ***u***_*d*_ and a common [*M* × *L*] dimensional matrix of inducing inputs ***Z***. Using a squared exponential [43] as covariance function

$$\begin{array}{c} k\left(x,x^{\prime }\right)=\sigma_{f}^{2}\exp(-0.5\sum_{l=1}^{L}\alpha_{l}{\left(x_{l}-x_{l}^{\prime }\right)}^{2}) \end{array}$$ (3)

allows [38] to derive an analytic lower bound for $\tilde{F}(Q)$. Eq (3) uses $\theta =[\sigma_{f}^{2},\alpha_{1},..,\alpha_{L}]$ as parameters of the covariance function. While *α*_*l*_ have an interpretation as inverse squared length scale in the latent dimension *l*, $\sigma_{f}^{2}$ represents a signal amplitude. The significance of introducing a sparse GP and a lower bound for $\tilde{F}(Q)$ is that [38] obtain an analytic expression $F_{Q}(Z,[\mu_{n},S_{n}\forall n],\theta )$ which bounds log(*p*(***Y***)) from below and allows for maximization w.r.t. the *variational* parameters ***Z***, [***μ***_*n*_, ***S***_*n*_∀*n*] and the parameters of the covariance function ***θ***.

The GP-LVM implementation by [38] requires in addition to chose between the *deterministic training conditional* (DTC) or the *fully independent training conditional* (FITC) parametrization of sparse GPs [60]. The maximum value of ${\text{max}}_{Z,[\mu_{n},S_{n}\forall n],\theta }(F_{Q})$ depends furthermore on the number of inducing values *M* and on the dimension of the latent space *L*. For applying GP-LVM we use the *BayesianGPLVM* model of the Python *GPy* package [61], induce sparsity by DTC and initialize the modes of *Q*(***X***) by the first *L* coordinates in principal component space. Except for the number of iterations which we set to 10000, optimization uses *BayesianGPLVM.optimize* with default parameters. This choice will optimize the lower bound of $F_{Q}$ with respect to the variational parameters ***Z*** and [***μ***_*n*_, ***S***_*n*_∀*n*] by using the L-BFGS-B optimizer of Python SciPy with default settings and iterations set to 10000. To obtain a reasonable number of inducing variables we set *L* to 36 and increase *M* until $\text{max}(F_{Q})$ reaches a plateau. The resulting *M* is then fixed and we determine the optimal latent dimension as $\hat{L}={\text{argmax}}_{L}(\text{max}(F_{Q}))$. The optimized Bayesian GP-LVM represents the 209 Nile tilapia images as a product of Gaussian distributions, $Q(X)=\prod_{n=1}^{N}N(X[n,:]|{\hat{\mu }}_{n},{\hat{S}}_{n})$. GP-LVM is meant to extract features from Nile tilapia images which allow predicting the lake of origin of the fish images. GP-LVM will however target image features irrespective of their biological relevance [36]. We refrain therefore from the proposition in [38, 59] to use GP-LVM as class conditional densities in a fully generative classifier. Instead we propose to use selected dimensions of the expectation *E*[***X***]_*Q*(***X***)_ as inputs for diagnostic classifiers after we convinced ourselves that the features represent genuine Nile tilapia morphology. As is pointed out by [43], we may use the size of the inverse length scales *α*_*l*_ to order the *L* dimensions of ***X*** by feature relevance. To elucidate the correspondence between latent dimensions and features in the Nile tilapia images we make use of the generative nature of GP-LVM. When fixing all but the *l*-th dimension of ***X*** to the respective sample mean, projecting the variability of dimension *l* with *p*(***Y***|***X***) to image coordinates illustrates how pixel variation in different image regions manifests itself in GP-LVM dimension *l*. Visualizations of the resulting saliency maps allow us to differentiate between latent GP-LVM dimensions which represent genuine Nile tilapia morphology and GP-LVM dimensions which are influenced by technical artifacts.

#### Predictive classification

To obtain robust analysis results we consider two classical machine learning methods to infer classifiers which are trained to predict the lake of origin when presented with GP-LVM or GPA transformed landmarks. To include a competitive approach which is based on classical neural networks, we use the hybrid Monte Carlo implementation for multi layer perceptrons (HMC-MLP) by R. Neal [44, 45]. As a second classical machine learning method we apply a Gaussian process classifier (GPC) [42, 43]. Using *C* = 6 as the number of lakes, predicting the lake of origin of Nile tilapia samples is a 1-of-*C* classification problem. A discrete column vector ***y*** denotes in this setting the lake of origin of every Nile tilapia sample. To obtain a measure of uncertainty when classifying test cases, the proposed approaches learn to predict the posterior probabilities of class. In preparation of model fitting the labels ***y*** have to be coded in a zero-one fashion such that ***Y*** is a [*N* × *C*] dimensional matrix of zeros, except for column *y*[*n*] in row *n* which we set to one. Denoting the classification inputs of one sample as *L*-dimensional vector ***x***, the probabilities, *P*(*y*|***x***), of 1-of-*C* problems are for HMC-MLPs obtained with the softmax transformation [42]. An implementation of HMC-MLP inference is provided by R. Neal at https://www.cs.toronto.edu/~radford/fbm.software.html.

Polychotomous 1-of-*C* classification with GPCs is most often approached with *C* binary classifiers. The model predicts in this case the zero-one coded ***Y*** matrix column wise by using a logistic sigmoid [42] as target transformation. Denoting the *L*-inputs of sample *n* as ***x***_*n*_ = ***X***[*n*,:]^*T*^, a GPC defines a Gaussian process in some latent map *f*(***x***_*n*_). If we condition on all training samples sample, *f*(***ξ***) has for a novel input ***ξ*** a univariate Gaussian distribution. GPC predictions of *C* *binary* class probabilities are obtained by integrating out *f*(***ξ***) from *P*(*y*_*c*_, *f*(***ξ***)|***ξ***). A subsequent normalization provides the 1-of-*C* probabilities as $P(y=c|\xi )=\frac{P(y_{c}=1|\xi )}{\sum_{k=1}^{C}P(y_{k}=1|\xi )}$. We apply in our analysis the GPC which is provided in *GPy* [61], use expectation propagation [62] with default parameters for inference and L-BGFS-B as optimizer.

GPC as well as HMC-MLP allow attenuating input features which do not aid classification. R. Neal coined in [44] the term *automatic relevance determination* (ARD) to denote this behavior. Introducing ARD in GPCs is a matter of using the squared exponential in Eq (3) as covariance function. The inverse squared length scales [*α*_1_, .., *α*_*L*_] which are inferred during inference can be used as ARD metric with larger values indicating more important inputs. Allowing for ARD during HMC-MLP inference requires specifying a hierarchical prior which governs the scale of a zero mean Gaussian prior over all input specific parameters. Our application relies on a prior which in the limit of an infinite number of hidden units converges to a Gaussian process. We consider separate ARD scales for input to hidden and input to output parameters and otherwise follow the default suggestions in the documentation [44]. While the number of inputs depends on whether we use GPA or GP-LVM features, all MLPs have 20 hidden units and six output units. The HMC simulations draw 2500 samples, discard the first 25% as burn in and extract independent predictions and ARD relevance scales from the remaining MCMC samples. Inference represents linear relevance, *σ*_*l*,1_, and nonlinear relevance, *σ*_*l*,2_, for every dimension *l* separately. Since HMC-MLP ARD values are prior scales, we suggest combining both relevance measure by summing squares, $\sigma_{l}=\sqrt{\sigma_{l,1}^{2}+\sigma_{l,2}^{2}}$. The same aggregation allows to interpret the relevance metrics of both landmark coordinates together. The *α*_*l*_ length scales in the squared exponential covariance function have a nonlinear effect on the covariance matrix of the Gaussian process. For combining GPC ARD metrics we suggest therefore to minimize the difference between the inferred covariance matrix and an approximation which results from using for all respective input dimensions the combined inverse length scales. To combine the inverse squared length scales [*α*_*l*_, .., *α*_*l*+*j*_] for input dimensions *l* to *l* + *j* to one $\alpha_{l}^{l+j}$ value, we obtain the approximate optimum as

$$\begin{array}{c} {\hat{\alpha }}_{l}^{l+j}=(\sum_{n}\eta_{n}^{T}L\eta_{n}\eta_{n}^{T}I\eta_{n})/(\sum_{n}{\left(\eta_{n}^{T}I\eta_{n}\right)}^{2}). \end{array}$$ (4)

Eq (4) expresses the original length scales as diagonal matrix ***L*** = diag(*α*_*l*_, ..*α*_*l*+*j*_) and denotes all samples of the corresponding input subsets as ***η***_*n*_. The ARD metrics which are obtained from ten fold cross testing are averaged and used to rank input features.

#### Convolutional neural networks

**Fig 3:**
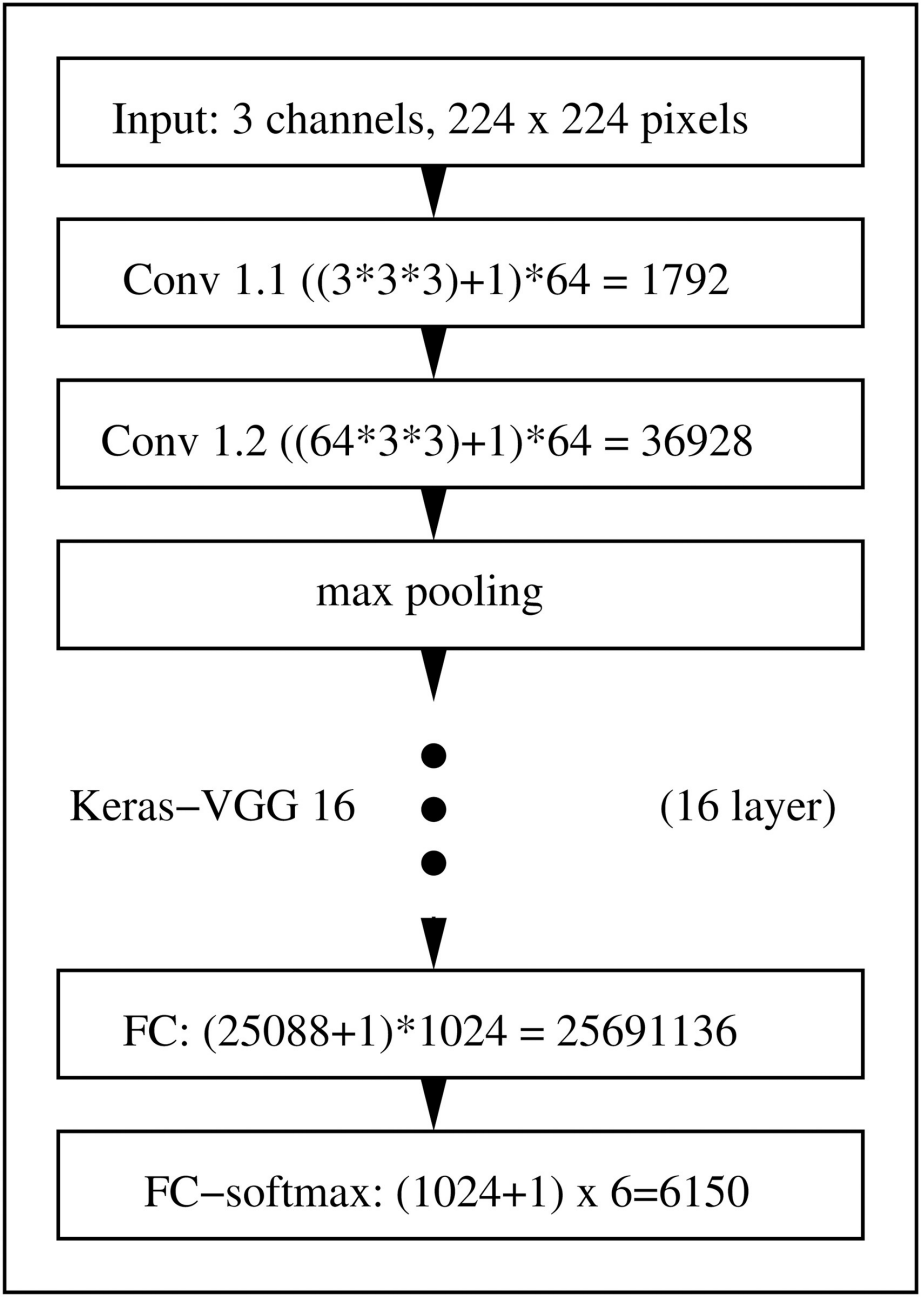
Sketch of the CNN architecture we use for predicting the lake of origin from 224 gray scale Nile tilapia images. Our architecture uses 16 layers. Except for the fully connected layers which use 1024 nodes and a 6 class softmax output layer, we apply the Keras version of VGG-16 by [24] which is inferred on ImageNet data [52].

Convolutional neuronal networks (CNN) were originally introduced in [25, 26] as optimal architecture for extracting information from images. Renewed interest in CNNs was triggered by [63] and CNNs since then regularly scoring top results in data analysis competitions. The CNN architecture which we apply for predicting the lake of origin from Nile tilapia images is the Keras [53, 54, 64] implementation of the ConvNet-D (VGG-16) architecture in [24]. Motivated by the success of VGG-16 with this resolution, we decided to use gray scale images with 224 × 224 pixels as CNN input. To allow using the pre-trained ImageNet [52] model of VGG-16 which expects three RGB channel images with a 224 × 224 resolution as input, we replicate the same gray scales in all three channels. Our CNN architecture, which we illustrate in Fig 3, follows largely the proposition in [24]. To fit to our Nile tilapia data, we replaced however their fully connected layers by a flatten layer, a dense layer with 1024 neurons, a dropout layer with a 50% dropout rate and a final dense layer with six neurons and softmax transformation.

Albeit relying on a pre-trained CNN, additional adaptation to our data set is crucial to reach good model performance. Satisfying results can be obtained with a procedure that was proposed by [24]. Training optimizes cross entropy, uses 70 epochs, a batch size of 8, the RMSprop optimizer with a learning rate of 10^−4^ and otherwise default settings. To achieve invariance to location, scale and orientation differences we follow [65] and use data augmentation with the following parameters: rescale = 1/255, rotation_range = 20, width_shift_range = 0.2, height_shift_range = 0.2, horizontal_flip = True and fill_mode = ‘nearest’.

The strength of CNNs to extract very subtle features lead to excellent performance characteristics which come however at the expense that the resulting models are complex and difficult to interpret. To avoid reporting results which are technically superior but result from the inclusion of rogue features, recent publications [36, 37, 48, 66–69] accentuate the importance of investigating how CNNs reach their decisions. We apply to this end the Grad-CAM [69] implementation in [70] and the implementation of layer wise propagation of relevance (LPR) [36, 37, 48] in [71]. Both approaches provide visual impressions about image regions which are important for trained CNNs to arrive for a test case at the respective prediction.

#### Statistical assessments of saliency maps

LPR [48] and Grad-CAM [69] provide quantitative assessments at pixel level about how different regions of a sample image contribute to the calculated predictions. By mapping the variation of individual latent dimensions into image space, GP-LVM provides comparable information for the entire data set. The LPR, GRAD-CAM and GP-LVM derived spatial importance metrics are positive, usually illustrated as saliency maps and interpreted manually. The decision whether a saliency map gives cause for concern may thus depend on the person assessing the map. To improve the reproducibility of determining image regions which are important for predicting the lake of origin of Nile tilapia samples, we suggest to assess the saliency maps with a statistical test.

To derive a test statistic a feature F which is represented by the convolution layers of VGG-16 or by the latent space of GP-LVM is considered as a set of *N* neighboring pixels, $F=[(\xi_{n},\eta_{n})\forall n\in [1,..,N]]$. The tuple (*ξ*_*n*_, *η*_*n*_) refers to the *x* and *y* coordinate of every pixel which characterizes the feature F. Spatial characteristics of extracted features like local persistence and sparsity is apparent in image processing literature (e.g. [54], chapter 5.4). Local persistence of feature coordinates implies that every feature pixel, (ξ,η)∈F, has a small neighborhood *ϵ*((*ξ*, *η*)) which contains other pixels which are also part of feature F. We may express this observation as $\exists (\xi_{\nu },\eta_{\nu })\in \epsilon ((\xi ,\eta ))|(\xi ,\eta )\in F\wedge (\xi_{\nu },\eta_{\nu })\in F$. Image regions which are important for model predictions will thus be characterized as comparably dense clusters of large LPR, GRAD-CAM or GP-LVM derived importance metric values. Such saliency maps may be described as marked spatial point processes (e.g. [72] chapter 6.4) on $\left({\mathbb{R}}^{2}\times {\mathbb{R}}^{+}\right)$ where ${\mathbb{R}}^{2}$ denotes the coordinate space of the spatial process which gives rise to a saliency map and ${\mathbb{R}}^{+}$ is the domain of the importance metric. Saliency maps are hence samples from a marked point process P(ξ,η)×G(γ|ξ,η), where P(ξ,η) determines the location of important image coordinates and G(γ|ξ,η) determines the location specific distribution of the importance metric. This notation allows expressing lack of importance of image regions as null hypothesis that the spatial process $P{\left(\xi ,\eta \right)}_{H0}$ on ${\mathbb{R}}^{2}$ which gives rise to an observed feature in the saliency is a homogeneous Poisson process. The expectation that important image regions are characterized by the existence of sparse clusters of large metric values suggests using a one sided alternative.

To obtain reproducible verdicts about image regions which contribute substantially to predictions, the proposed test is applied on a pixel per pixel basis in a non-parametric manner.

1. We start by regarding a saliency map to be a sample which was generated under the alternative hypothesis $P{\left(\xi ,\eta \right)}_{H1}\times G{\left(\gamma |\xi ,\eta \right)}_{H1}$.
2. A sample of the null hypothesis $P{\left(\xi ,\eta \right)}_{H0}\times G{\left(\gamma |\xi ,\eta \right)}_{H0}$ in the marked space is obtained by randomly reshuffling all *x* and *y* coordinates and rearranging the importance metric values of the original saliency map.
3. To assess the null hypothesis against the alternative at position (*x*, *y*), we compare the (*x*, *y*) surrounding local averages in the saliency map which represents the null hypothesis against the respective average value of the original saliency map. For calculating averages we convolve the saliency maps with a *k* × *k* pixel wide Gaussian kernel to obtain *g*_*H*1_(*x*, *y*) for the alternative hypothesis and *g*_*H*0_(*x*, *y*) for the null hypotheses. Using a one sided alternative implies that we obtain evidence in favor of the null hypothesis *H*0 if *g*_*H*0_(*x*, *y*)≥*g*_*H*1_(*x*, *y*).
4. A p-value map is obtained by iterating sample generation under the null hypothesis *N* times, where *N* is sufficiently large and counting for every location the number of instances *n*(*x*, *y*) for which we obtain evidence in favor of *H*0. Location specific p-values are obtained as ratio
  $$\begin{array}{c} \text{p-val}(x,y)=n(x,y)/N. \end{array}$$ (5)
5. To obtain a reproducible visualization from the saliency map we use a p-value threshold of 0.001 to obtain a binary mask which indicates image regions which are by LPR, GRAD-CAM or GP-LVM considered important for the calculated predictions. The sample specific nature of LPR and GRAD-CAM suggests to display the discretized p-value map on top of the image to highlight important image features. The saliency map which we obtain for GP-LVM assesses the data set in its entirety. In this case we use the binary mask to set all saliency values in irrelevant image regions to zero.

The implementation of this test relies on the Python OpenCV interface for calculating convolutions and sets *k* = 5 and *N* = 10^4^.

#### Performance metrics

To assess whether geographic variation exists in Ethiopian Nile tilapia body features, we perform in this paper an indirect analysis. The existence of population specific morphotypes would imply that image derived features contain information about the lake of origin of fish. If such information exists, we expect to obtain reasonably good performance characteristics. Drawing respective conclusions depends therefore on quantitative performance metrics which allow us to compare classifiers. While we did already point out that we use ten fold cross testing [46] to obtain unbiased estimates, we will now summarize three complementary metrics of classification performance.

1. Classification accuracy (Acc) is the most commonly applied performance metric for classifiers [46]. Evaluating classification accuracy requires us to determine the rate of agreement between predicted and true labels. If we use *N* to denote the number of samples and use ${\hat{y}}_{n}$ as predicted and *y*_*n*_ as true label and define $\delta ({\hat{y}}_{n}=y_{n})=1$ if labels agree and $\delta ({\hat{y}}_{n}=y_{n})=0$ if labels disagree, generalization accuracy is expressed as
  $$\begin{array}{c} \text{Acc}=\frac{100}{N}\sum_{n=1}^{N}\delta \left({\hat{y}}_{n}=y_{n}\right)\%. \end{array}$$ (6)
  Rating a classifier as reasonably good requires that its *Acc* is larger than the *Acc* obtained when predicting the majority label.
2. While Acc captures predictive accuracy, taking posterior probabilities for class, *P*(*y*|***x***_*n*_), into consideration provides further insight. Mutual information (MI) [73], *I*(***x***, *y*), between input ***x*** and class labels *y* provides us with such a metric. We obtain
  $$\begin{array}{c} I\left(x,y\right)\approx \frac{1}{N}\sum_{n=1}^{N}\sum_{y}P\left(y_{n}=y|x_{n}\right)\log_{2}(\frac{P(y_{n}=y|x_{n})}{P(y_{n}=y)}), \end{array}$$ (7)
  where use of log_2_ leads to quantifying MI in bit. To rate a classifier by MI as reasonably good we require that *I*(***x***, *y*) is larger than zero.
3. Assessing classifiers is in statistical terminology equivalent to conducting an experiment. Repeating the assessment will thus inevitably change *Acc* and *I*(***x***, *y*). To ensure that differences are systematic requires confirming that randomness could not have caused the differences. For assessing classifiers *a* and *b* McNemar [74, 75] proposed an optimal paired significance test. McNemars statistic consists of counts *n*_*a*_ and *n*_*b*_. While *n*_*a*_ counts the number of samples which were correctly predicted by classifier *a* and wrongly classified by classifier *b*, *n*_*b*_ counts the number of samples that were wrongly classified by classifier *a* and correctly predicted by classifier *b*. McNemars procedure is based on the idea that differences between both classifier arise by chance if (*n*_*a*_, *n*_*b*_) has a Binomial distribution with count *n*_*a*_ + *n*_*b*_ and probability 0.5. Denoting the probability mass of the binomial distribution as $P_{B}((k,l);k+l,0.5)$ and assuming a two sided alternative hypothesis, we get the McNemar p-value (Sig) as
  $$\begin{array}{c} p=2\sum_{n=0}^{\min(n_{a},n_{b})}P_{B}\left(\left(\min\left(n_{a},n_{b}\right)-n,\max\left(n_{a},n_{b}\right)+n\right);n_{a}+n_{b},0.5\right). \end{array}$$ (8)
  A reasonably good classifier will in comparison with predicting the majority label for all samples obtain a McNemar p-value, *p* < 0.05. For visualization purpose, we truncate p-values to be smaller than 0.99 and apply a logit transformation logit(*p*) = log(*p*) − log(1 − *p*).

Generalization accuracy, mutual information and McNemars significance test allow investigating whether Nile tilapia images contain information about the origin of fish samples. Mutual information provides as the name suggest a direct quantification of information content. Generalization accuracy and McNemars test require however to compare the image and GPA-shape based classifiers against predicting the majority label. Predicting the majority class is an input independent lower bound for generalization performance. To substantiate that Nile tilapia morphology depends on the lake of origin, respective classifiers must hence significantly outperform predictions which only depend on counting labels [46].

### Availability

To allow reproducing our findings we provide all fish images, raw landmark coordinates and annotation information as S1 File. All data and code from this paper are in addition available in a public GitHub repository under GPL v3 license. The repository can be reached at https://github.com/TW-Robotics/NT_BodyParts. Details on how to use the repository are provided online as markup file.

## Results

The results section provides first some insight into GPA and GP-LVM features. We will subsequently assess the results which we obtain from all analysis pipelines in Fig 1 on a global scale by looking at generalization accuracy, mutual information and McNemar p-values. To rule out that non biological information in the images about lake impedes drawing reliable biological conclusions, we provide a careful assessment of image features which are extracted by GP-LVMs and CNNs. The ARD metrics of GPC and HMC-MLP will finally be used to identify GPA transformed landmarks and GP-LVM image regions of Nile tilapia which our analysis considers relevant for predicting sample origin. Based on this analysis we identify Nile tilapia body features that contribute most to the divergence between populations. The identified differences in Nile tilapia morphology may be related to adaptation processes, change events, or result from phenotype plasticity.

### GPA transformed landmarks

**Fig 4:**
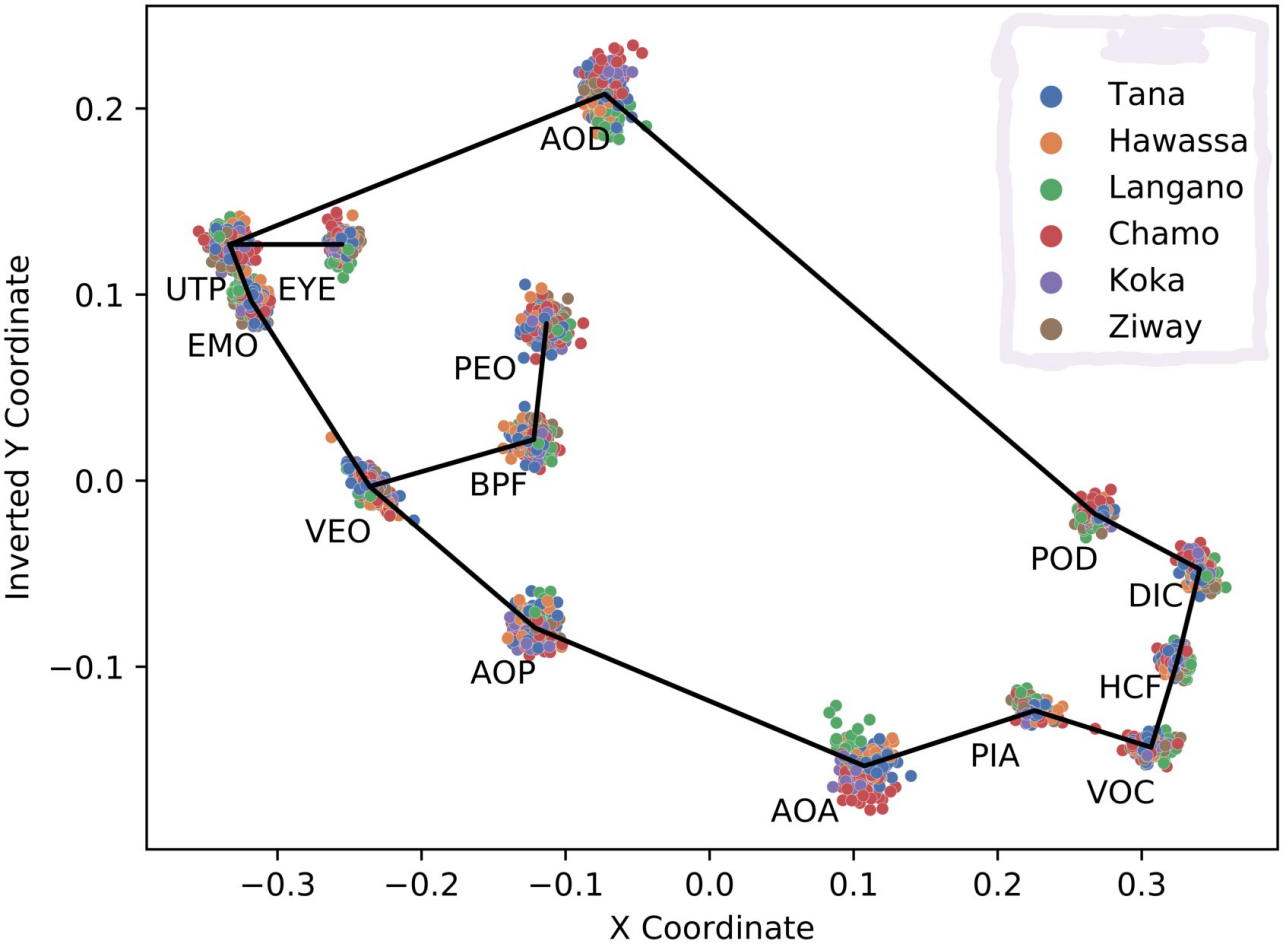
Scatter plot of GPA transformed landmark positions. The coordinates of the landmarks listed in Table 2 are processed with a generalized Procrustes analysis. After color coding by lake, the 14 two dimensional landmark coordinates of all Nile tilapia samples are visualized as dots. The solid line connects the center locations of all landmarks.

We use the manually assigned landmarks which we transform to GPA scale features as benchmark solution for assessing information contained in Ethiopian Nile tilapia fish about their respective habitat. The 28 GPA scale features which we illustrate in Fig 4 are labeled by the respective lake of origin. Gaussian process and HMC-MLP classifiers are subsequently used for predicting the lake from the respective GPA features. Performance metrics provide an indication of whether Nile tilapia populations show signs of adaptation to origin. ARD based rankings provide information about which Nile tilapia body parts contribute the most to differentiating between populations.

### GP-LVM features

**Fig 5:**
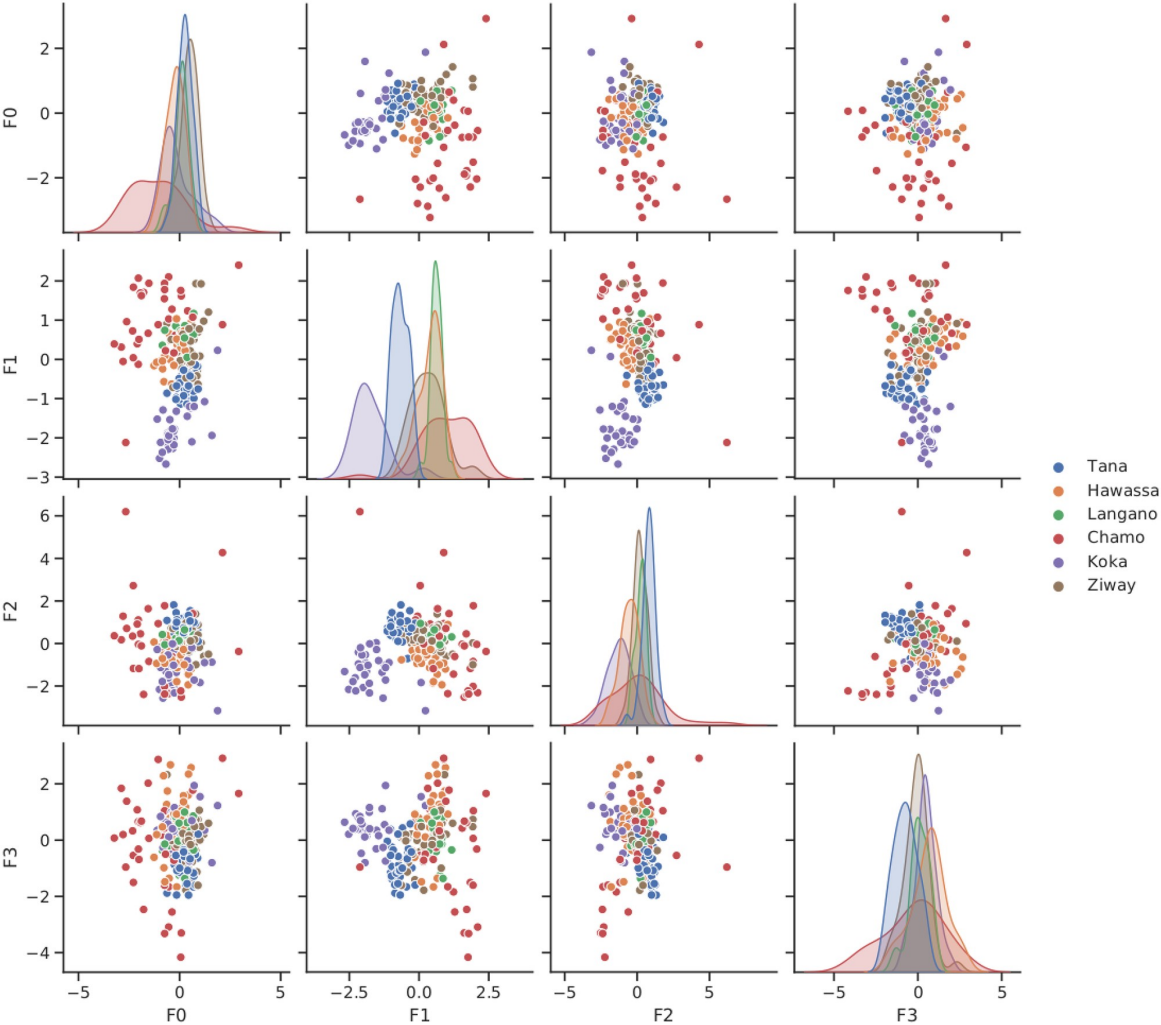
Visualization of GP-LVM projections. A scatter plot matrix provides pairwise impressions of the latent dimensions F0 to F3. Coloring by lake shows that dimension F0 separates lake Chamo from the other lakes while dimensions F1 and F2 show distinct values for lakes Koka and Tana.

To calibrate the number of inducing points GP-LVM inference is run with a latent dimension of 36. By visual inspection of the approximate log marginal likelihood (data not shown) it became evident that using more than 100 inducing points has only minor effects on the approximation. A subsequent search for optimal model complexity revealed that the approximate log marginal likelihood peaks, if we set the latent dimension to 100 as well. It is important to point out that this approach to model selection considers the representational capabilities of GP-LVM as generative model for unsupervised image analysis. The optimal number of features for the supervised task of predicting the lake of origin of Nile tilapia images may be considerably smaller. To use a feature vector which is comparable with the number of landmarks, 14 of the 100 features were selected as inputs for classification. Fig 5 visualizes the expected values of *Q*(***X***) in the first four GP-LVM dimensions as scatter plot matrix. Coloring by lake shows that dimension F0 allows distinguishing samples from lake Chamo from the samples gathered at the other lakes. Dimensions F1 and F2 a clearly distinct for lakes Koka and Tana.

### Performance metrics

To define population specific morphological features robustly we apply several data analysis pipelines in parallel. Table 3 reports for all analysis pipelines the generalization accuracy (Acc), the mutual information (MI) and the McNemar p-value (Sig) which we obtain by averaging over ten randomizations. The analysis pipelines are denoted by abbreviations. We use Deep CNV+CNN for the convolutional neural network, GPA+GPC for the Gaussian process classifier on Procrustes shape features, GPA+HMC-MLP for the Hybrid Monte Carlo multi layer perceptron on Procrustes shape, Top GP-LVM+GPC for the Gaussian process classifier on 14 most relevant GP latent variables as rated by GP-LVM ARD levels, Top GP-LVM+HMC-MLP for the Hybrid Monte Carlo multi layer perceptron on 14 most relevant GP latent variables, Sel GP-LVM+GPC for the Gaussian process classifier on 14 selected GP latent variables and Sel GP-LVM+HMC-MLP for the hybrid Monte Carlo multi layer perceptron on 14 selected GP latent variables. The McNemar p-values compare the GPA+GPC pipeline which achieved the least generalization accuracy against all other classification results.

**Table 3:** Average performance metrics of all tested classifiers.

Method	Acc [%]	MI [bit]	Sig
Deep CNV+CNN (VGG-16)	93.3	2.53	≪0.001
GPA+GPC	70.7	1.05	– – –
GPA+HMC-MLP	75.0	1.66	0.15
Top GP-LVM+GPC	92.5	1.38	≪0.001
Top GP-LVM+HMC-MLP	91.6	2.12	≪0.001
Sel GP-LVM+GPC	72.1	1.03	0.80
Sel GP-LVM+HMC-MLP	74.4	1.54	0.37

This table summarizes the average generalization accuracy (Acc) in percent, average mutual information (MI) in bit and McNemar significance (Sig). McNemar p-values result from pairwise comparisons between Gaussian process classification of GPA landmarks and six other combinations of input features and classification methods. The assessed classification methods are VGG-16, a 16 layer convolutional neural network (Deep CNV+CNN), a Gaussian processes classifier (GPC) and a multi layer perceptron inferred with hybrid Monte Carlo sampling (HMC-MLP). The assessed input features are generalized Procrustes analyzed landmarks (GPA) and the expectations in feature space of a Gaussian process latent variable model (GP-LVM). For the latter feature representation we considered two different selections: Top GP-LVM refers to the 14 most relevant latent dimensions and Sel GP-LVM refers to the 14 highest ranked dimensions with features excluded which by showing variation in the image background were suspected to be influenced by technical artifacts.

**Fig 6:**
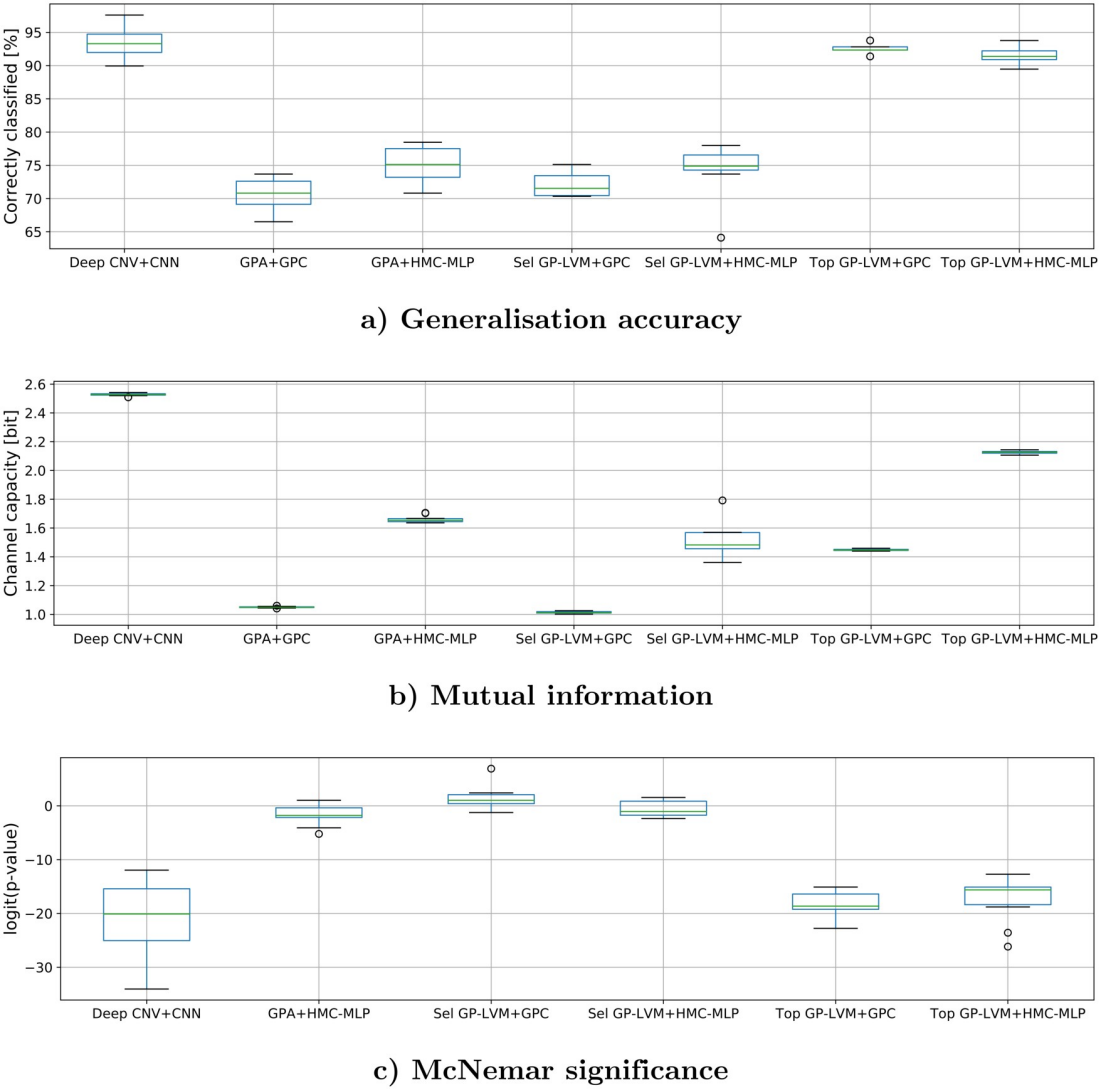
Box plots of generalization metrics that were obtained by resampling. The metric values which give rise to these box plots were obtained by ten fold cross testing after reshuffling the samples ten times. Every box illustrates the performance characteristics of a distinct combination of input feature and classifier. A description of the acronyms is provided in the legend of Table 3 and in the text. a) illustrates generalization accuracies in percent. b) illustrates the distribution of Mutual information and c) visualizes the McNemar p-values on a logit scale when comparing GPA+GPC against the other analysis pipelines.

To assess the range of values which we obtain by randomizing, Fig 6 visualizes individual metric values which were obtained on reshuffled data as box plots. The results in Table 3 and Fig 6 suggest that there are two groups of analysis pipelines. A group of methods with significantly higher generalization accuracy and larger mutual information consists of VGG-16 and both stand alone classifiers when using the 14 most relevant GP-LVM features. A second group with significantly lower generalization accuracies and mutual information contains the stand alone classifiers when we use 14 selected GP-LVM features or the Procrustes transformed landmarks as inputs. When interpreting the results, we should first note that the selected 14 GP-LVM features were carefully chosen: a GP-LVM dimension would only be used if feature specific variation in image space gave no concern for underlying technical contamination. This selection anticipates that variation which does not originate from genuine fish attributes might cause the improved results which we observe with some of the analysis methods.

### Features in image space

**Fig 7:**
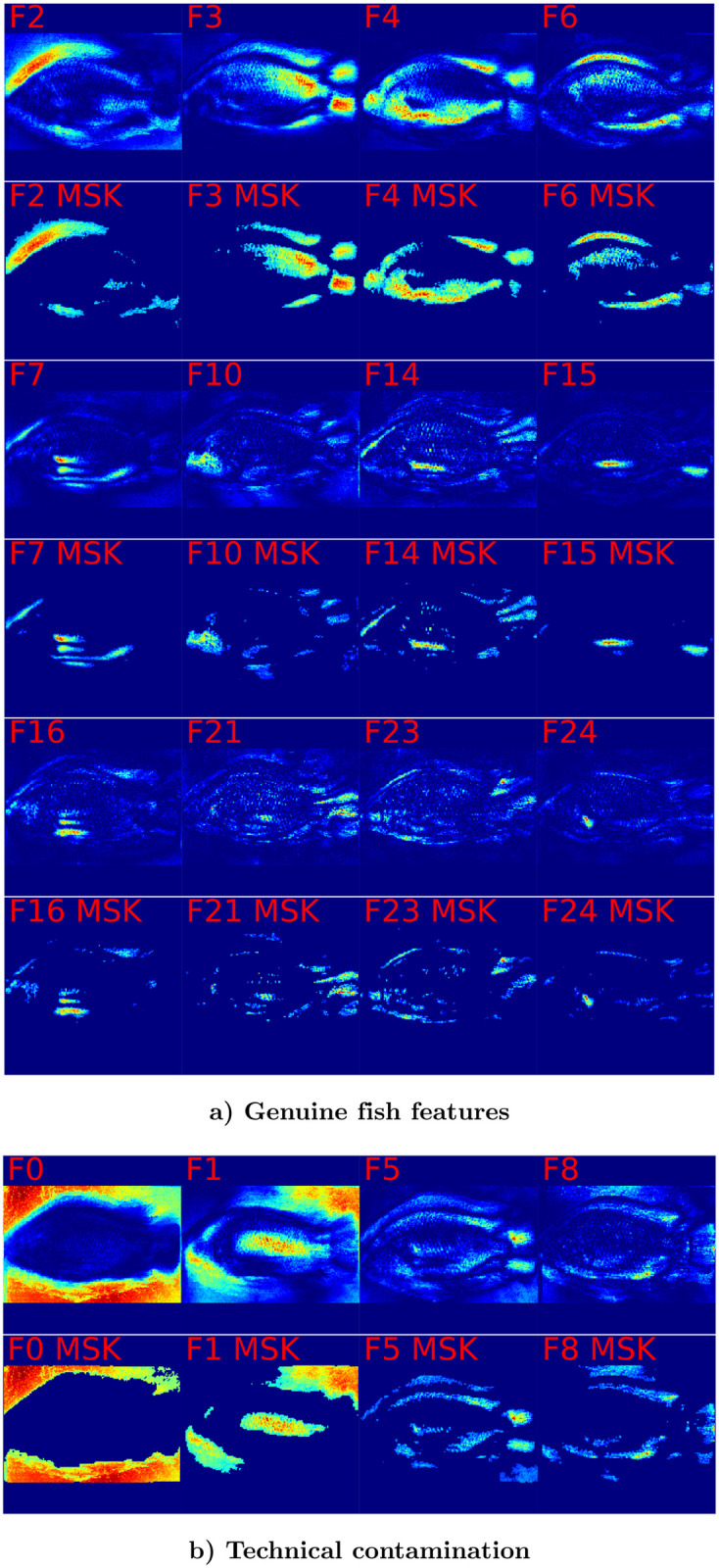
Variation of GP-LVM feature dimensions in image space. Image color is obtained by mapping variation of individual GP-LVM dimension to image coordinates. Low variation is represented as blue color. Intermediate variation has yellow color and red represents image regions which correspond to large variation in GP-LVM space. Odd rows display the entire saliency map. Even rows retain only highly relevant pixels which are by Eq (5) assessed as significant (p-value <0.001). Fig 6a) illustrates 12 GP-LVM dimensions which represent genuine fish morphology or skin markings and allow to draw valid conclusions about location specific adaptation. Fig 6b) illustrates 4 rogue GP-LVM feature dimensions which represent technical artifacts. The improved performance we observe in Table 3 for Top GP-LVM inputs is thus aided by technical artifacts which are correlated with the class label lake.

To investigate in detail how the analysis pipelines which we compare in Fig 6 and Table 3 arrive at their predictions, we have to investigate which image features are deemed important for predictions. As is further detailed in the methods section, the values in the GP-LVM saliency maps in Fig 7 make use of the generative nature of GP-LVM and are projections of variation in the latent space of the GP-LVM. To provide a more objective visualization of image regions which contribute to the latent features, insignificant entries of the saliency map are set to zero. The mask which is used for that purposes is a result of the p-value calculation in Eq (5) and applying 0.001 as significance threshold. The layout of Fig 7 is such that even rows illustrate the original saliency maps and odd rows show the masked saliency maps. Individual images in Fig 7 illustrate the projections of selected latent GP-LVM dimensions and are tagged as such. Blue color indicates regions which show little influence and red highlights the most influential image regions. Fig 7a) displays 12 of the 14 GP-LVM features which result in a classification performance which is comparable with the Procrustes landmark based classifications. We may confidently conclude by visual inspection of the masked map that variation of Nile tilapia body features gives rise to these latent variables. Fig 7b) visualizes 4 of the 14 top ranked latent GP-LVM dimensions which show noticeable to large response to variation in the image background. These latent dimensions are in part caused by technical variation and thus not entirely rooted in fish phenotype. The improved results which we obtain in Table 3 for the the two analysis pipelines which use the 14 top ranked GP-LVM dimensions are hence aided by technical variation which is correlated with the class label. Observing large generalization accuracy is thus not sufficient to justify statements that Nile tilapia morphology adapts to the fish habitat.

**Fig 8:**
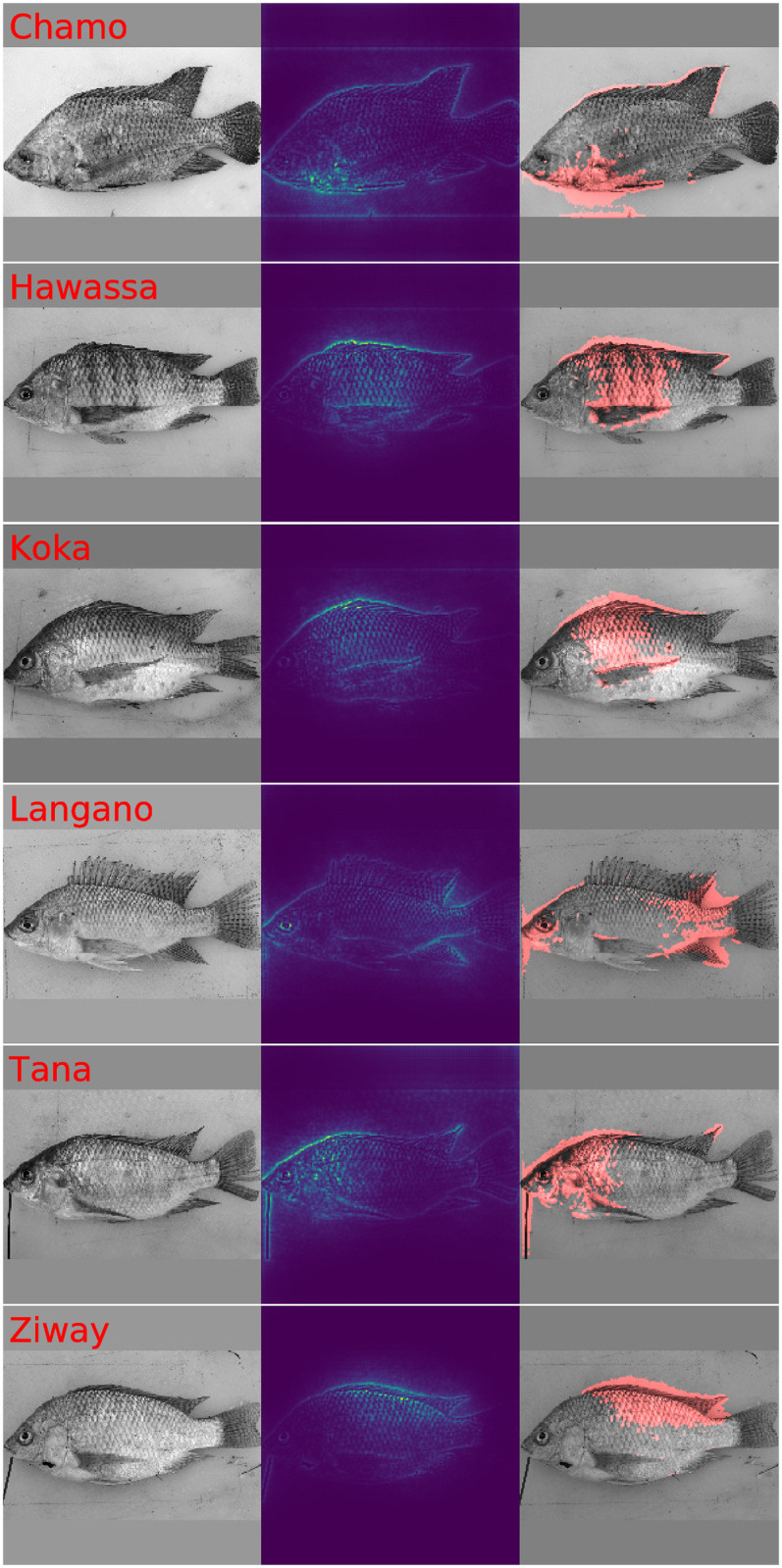
LPR diagnostic plots for highly representative Nile tilapia samples. This figure illustrates fish images, LPR saliency maps and diagnostic plots which display fish images and red color mask to highlight image features which contribute significantly to the prediction. The chosen samples are highly representative for the respective lake of origin. While the predictions of the samples for lakes Hawassa, Koka and Ziway are fine, the predictions for lake Chamo and Langano use information in the image background. The prediction of the lake Tana sample is aided by the presence of the fixation pin.

**Fig 9:**
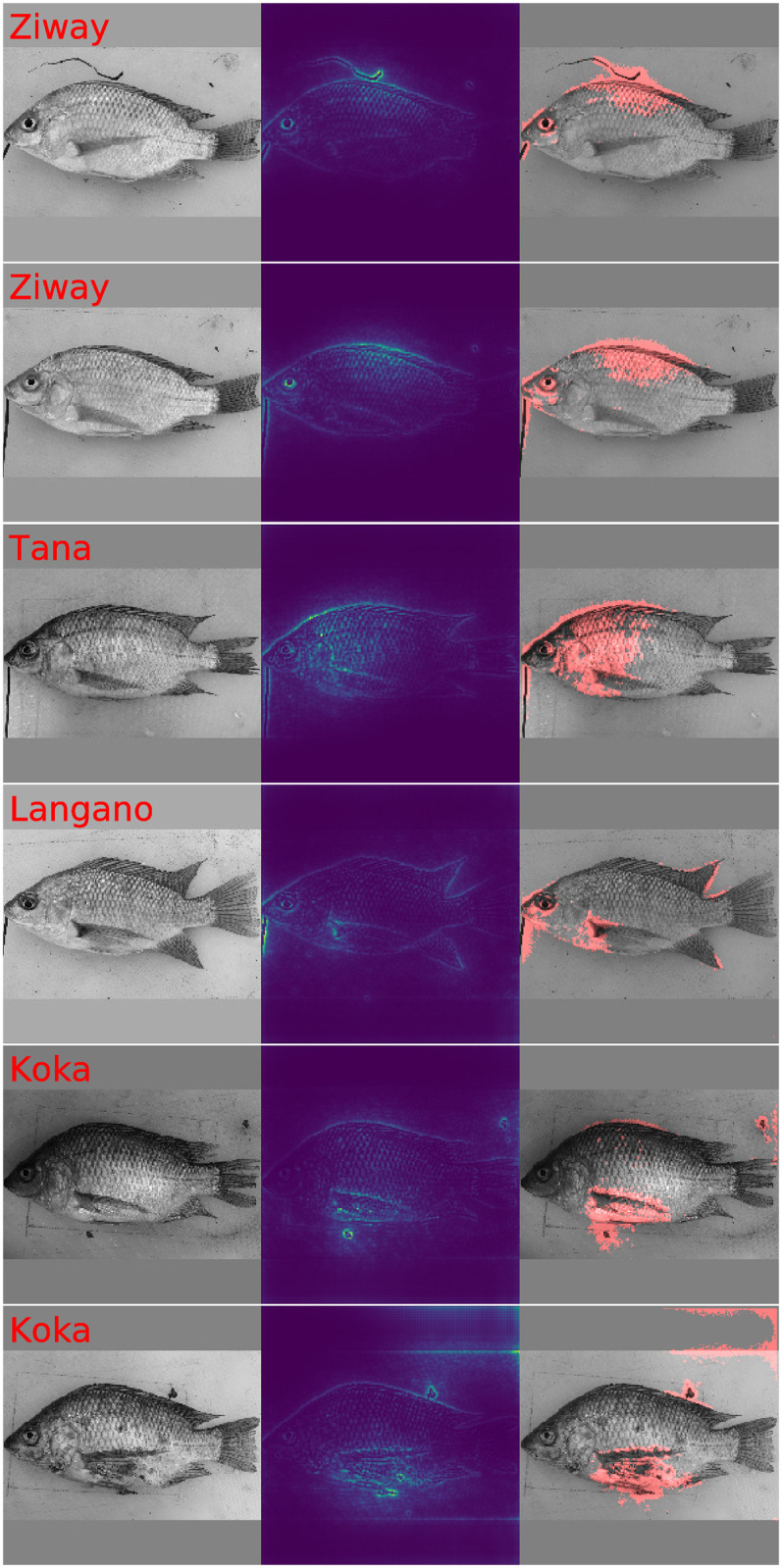
Selected LPR diagnostic plots with visible contamination by technical artifacts. This figure illustrates fish images, LPR saliency maps and diagnostic plots which display fish images and red color masks to highlight significant image features. All images contain indications that genuine Nile tilapia body features are considered relevant for predicting the lake of origin. It is however also obvious that features in the image background, dirt particles and the presence of the fixation pin provide rogue information which aids predicting the respective lake of origin.

To provide insight about which features in images give rise to the CNN predictions Fig 8 displays selected saliency maps which we obtain with LPR [48, 71]. The chosen Nile tilapia images resulted for the respective lakes in largest posterior probability. The first column in Fig 8 displays the sample image. The second column displays the LPR based saliency map which represents the importance of image pixels for predicting the lake of origin by a color scheme. Blue indicates pixels with low importance, yellow denotes mid important pixels and red is used for very important pixels. To obtain an objective indication for important regions we calculate pixel specific p-values according to Eq (5) and construct a binary map by applying a p-value threshold of 0.001. The third column of Fig 8 illustrates the sample image and displays the resulting importance mask as overlay in transparent red. Inspecting the saliency maps it is apparent that the CNN finds for all samples genuine morphological features of Nile tilapia which are indicative of the lake of origin. While the samples for lakes Hawassa, Koka and Ziway give no reason for concern, we observe for lakes Chamo and Langano that the image background and for lake Tana that the fixation pin have a significant influence on the predicted probabilities. It is worth noting that the fixation pin has no influence in the prediction given for the lake Ziway sample. Whether or not a particular artifact provides information for a prediction is sample dependent. The visualization in Fig 9 which has the same structure as Fig 8 shows examples where rogue image features like dirt particles, variation of the image background and the occasional presence of the fixation pin contribute significantly to predicting the lake of origin. We conclude therefore that the performance metrics for the CNN in Table 3 are not only a result of genuine differences in Nile tilapia phenotype but aided by artifacts which show some correlation with lake of origin. The GPA-shape features and the 14 carefully selected GP-LVM projections are a more reliable representation of visual differences of Nile tilapia which are biologically meaningful. Visualizations of Grad-CAM [69, 70] derived saliency maps are provided as S1 and S2 Figs. While Grad-CAM corroborates that predictions samples are influenced by features in image regions which are clearly not related to the fish, the maps provide much coarser impressions.

### Detection of Nile tilapia population specific morphology

The visualizations in Figs 7 and 9 hint visual features of Nile tilapia which are indicative for the lake of origin. The visualization however also shows that the CNN and classification with the 14 top ranked GP-LVM features overestimate the amount of information about lake specific variation of Nile tilapia morphology. To assess whether population specific morphotypes exist we compare the results in Table 3 against majority vote based predictions. Table 1 reports for lake Ziway a maximum of 40 samples. Predicting lake Ziway for all samples results therefore in a generalization accuracy of 19%, a mutual information of 0 bit and in McNemar p-values ≪0.001 when comparing the majority rule with the predictions of all other analysis pipelines. The result that Nile tilapia images contain information about the lake of origin is hence observed for all data analysis pipelines. We can thus safely conclude that there are population specific morphotypes which are likely the response to environmental and ecological factors [12, 15–17].

**Fig 10:**
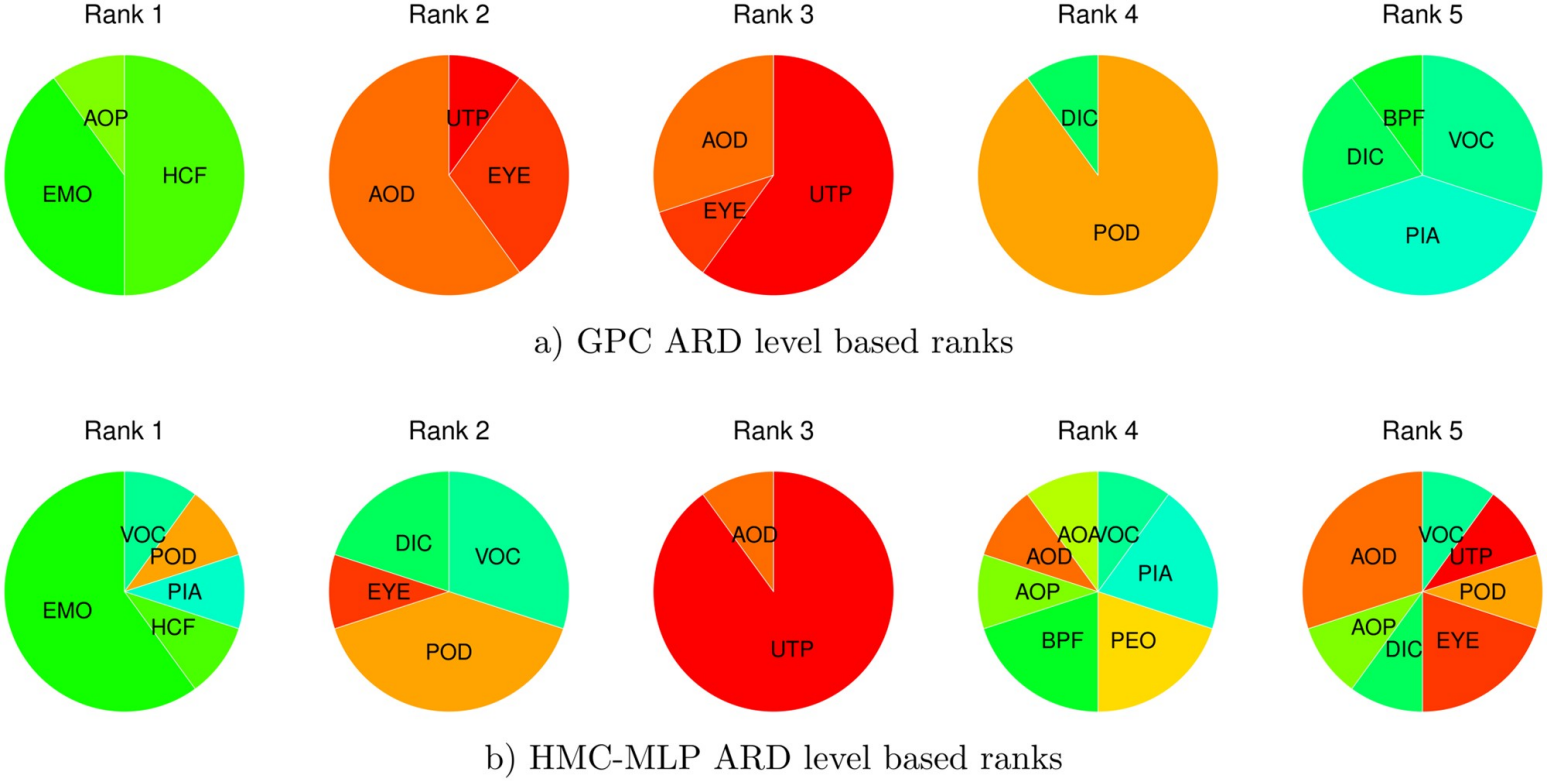
ARD level based ranking of GPA-shape features. The pie charts visualize for ranks one to five the relative number of occurrences of a landmark being ranked at the respective position when repeating inference ten times on reshuffled data. The graph in a) illustrates the ranks which we obtain with GPC based ARD levels. The graph in b) illustrates the ranks which we obtain with HMC-MLP based ARD levels. For improved reproducibility we look for agreement between both rankings to conclude that the upper tip of the snout (UTP), the posterior end of the mouth (EMO), the anterior insertion of the dorsal fin (AOD), and the posterior insertion of the dorsal fin (POD) show signs of differentiation. This suggests that dorsum and snout are affected the most from adaptation.

To obtain a better understanding about which features contribute the most to morphoological differences, we have a closer look at the inferred ARD hyper-parameters of GPC and HMC-MLP. We visualize to this end the ARD level based rankings of the GPA-shape transformed landmarks. Fig 10 displays the results separately for the Gaussian process classifier and the hybrid Monte Carlo inferred MLP. To arrive at robust conclusions we look for agreement between both classification results. Both rankings agree that the upper tip of the snout (UTP), the posterior end of the mouth (EMO), the anterior insertion of the dorsal fin (AOD), and the posterior insertion of the dorsal fin (POD), contribute to shape variation. We may therefore conclude from the GPA shape features that the features that are more important to define population specific morphotypes are the dorsum and the snout of Nile tilapia.

**Fig 11:**
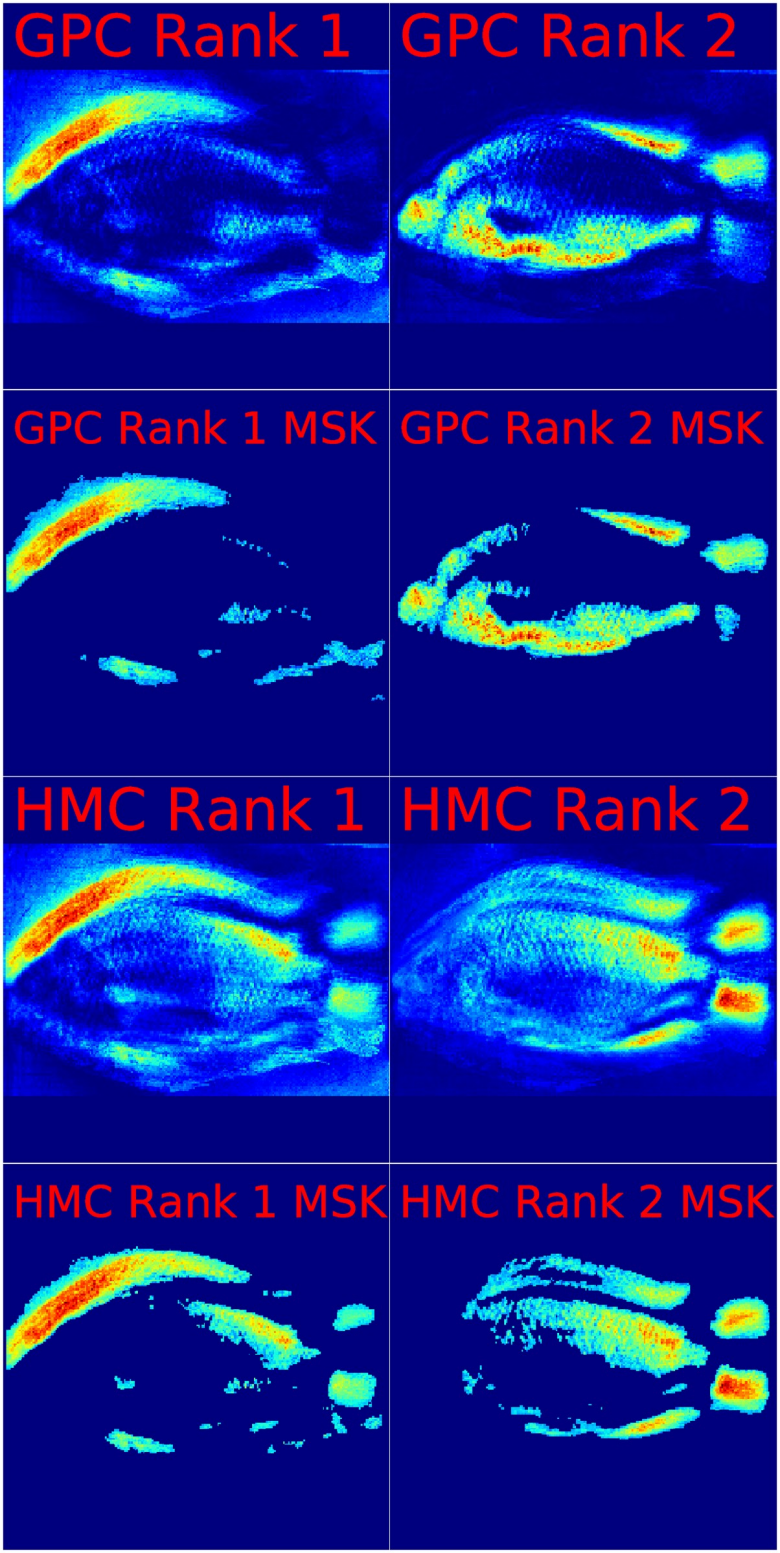
ARD level based visualizations of GP-LVM features in image space. This figure illustrates Nile tilapia body regions which are indicative for sample origin. Regional importance is visualized as color transition with blue indicating little importance, yellow indicating intermediate importance and red indicating high importance. Importance of image regions combines the GP-LVM variation maps in Fig 7 with the ARD level based ranks which obtain by classifying the lake of origin from 14 selected GP-LVM dimensions with Bayes inferred GPC and HMC-MLP. Odd rows in Fig 7 show the importance levels of the top two rank positions. Even rows focus attention on image regions which are by Eq (5) assessed as significant (p-value <0.001). Visualizations are tagged by the classification procedure which provides the ARD levels for ranking. With red indicating important image regions, the visualizations allow the conclusion that the anterior dorsal region, the belly region, the posterior dorsal region and the caudal fin of Nile tilapia are indicative for the origin of fish specimen.

To investigate which Nile tilapia body features are picked up by GP-LVM for distinguishing the lake of origin, we combine the GPC and HMC-MLP ARD level ranks with the representation of GP-LVM feature variation in image space. Fig 11 illustrates to this end weighted averages of the GP-LVM visualizations in Fig 7. The visualizations in Fig 11 result from using the relative number of occurrences of the first and second ranked GP-LVM dimensions when ordering by ARD levels. Individual saliency maps are tagged by the classifier which provides the ARD levels. Odd rows display the entire saliency map. Even rows focus attention on image regions which we assess by p-values calculated according to Eq (5) as significant (p-value <0.001). Irrespective of whether we rank by GPC ARD or HMC-MLP ARD we can see that the anterior dorsal region of Nile tilapia is the most important feature of fish for adaptation to habitat. This observation corroborates the result which we observe on the GPA-shape based assessment that landmarks in the anterior and dorsal regions vary on dependence of the samples lake of origin. By investigating the second rank positions we can deduce that both classifiers also agree about the contribution of the belly region, posterior dorsal region and to some extent the caudal fin to shape differences of Nile tilapia.

## Discussion

We hypothesize in this paper that complementary analyses are required to reliably infer whether Nile tilapia morphology is population specific. This hypothesis is evaluated against the alternative hypothesis that applying *one* machine learning method naïvely may result in conclusions about Nile tilapia phenotype being affected by technical artifacts. Established knowledge [35, 51] lead us to suggest that a technical reliable solution should look for agreement among several methods. We use to this end GPA scale transformed landmarks [40, 41] to capture morphology and apply GP-LVM [38] to extract visual features of Nile tilapia samples. GPA scale features and the GP-LVM representation are subsequently used as inputs to GPCs [42, 43] and HMC-MLPs [44, 45] to predict the lake of origin of 209 Nile tilapia samples. To compare the results which we obtain with classical ML with modern approaches, we furthermore include the Keras implementation of the VGG-16 deep learning architecture [24, 64] in our evaluation.

While looking for agreement among state of the art ML secures a technically optimal solution, recent discoveries by [37, 48, 69] suggest that excellent performance may be aided by what [36] call a *Clever Hans* predictor. Artifacts in the input data which happen to be correlated with the target label “lake” will aid predictive accuracy and lead to overly optimistic biological conclusions. We find that LPR generated feature maps find the occasional presence of a fixation pin, variation in image background and dirt particles important for predicting the lake of origin of Nile tilapia samples. This result provides clear evidence that obtaining biologically sound conclusions depends on complementary assessments to agree about findings and to rule out that reported biological findings are a result of artifacts in the data.

To avoid that we misjudge rogue features as evidence of true morphological change, we subsequently removed suspicious GP-LVM features. Despite that the reduced feature set lead to a significant loss in generalization performance, the results still allow concluding that there are population specific morphotypes. An investigation of ARD based ranks of landmarks in GPA shape representation in Fig 10 reveals that the snout and the anterior dorsum of Nile tilapia are most susceptible to adaptation. An investigation of weighted image representations of important GP-LVM feature variability in Fig 11 corroborates a predominant susceptibility of the anterior dorsum to adapting to fish habitat. The visual representation of the weighted GP-LVM features also reveals that the fish belly, the posterior dorsal region and the caudal fin show signs of differentiation which are likely the response to environmental and ecological factors [12, 15–17].

An elaborate ML analysis has thus proven potential to provide new insight into how Nile tilapia morphology changes. The combination of GP-LVM, a careful feature analysis and subsequent classification has the advantage of providing an unbiased view which detects morphological features irrespective of where landmarks are placed. Even if we have to investigate GP-LVM characteristics to rule out that biological conclusions are misled by image artifacts, a GP-LVM based analysis needs moreover considerably less time than manually placing landmarks in every Nile tilapia image. Carefully designed ML data analysis pipelines are thus very useful for research tasks which fall into the domain of classical morphometrics.

A final remark about ML driven knowledge discovery concerns the importance of careful data collection. To safeguard obtaining biologically meaningful results the experimental plan has to avoid introducing artifacts which are correlated with the dependent variable. State of the art ML methods are extremely powerful and will use all information which aids predictions. If discriminatory information which has no biological origin remains undetected, the conclusions drawn from such analysis may be misleading. Analyses which aim at identifying differentiated morphological features from sample images must keep technical variation in images like the orientation of samples, the placing of fixation pins and the image background at an absolute minimum.

## Supporting information

S1 Fig GRAD-CAM diagnostic plots for highly representative Nile tilapia samples. This figure illustrates fish images in column one, GRAD-CAM diagnostic plots in column two and fish images overlayed with a red colored GRAD-CAM derived significance mask in column three (p-val<0.001, calculation according to Eq (5)). Visualization are tagged by lake of origin and show samples which are highly representative for the respective lakes. While the samples of lakes Hawassa, Koka, Tana and Ziway raise no concern, the visualization of the samples for lakes Chamo and Langano suggest a significant dependency of predictions on the image background.
(TIF)

S2 Fig GRAD-CAM diagnostic plots with visible contamination by technical artifacts. This figure illustrates fish images in column one, GRAD-CAM diagnostic plots in column two and fish images overlayed with a red colored GRAD-CAM derived significance mask in column three (p-val<0.001, calculation according to Eq (5)). Except for the lake Tana specimen all samples show that predictions are aided by contamination with technical artifacts.
(TIF)

S1 File Data archive. To allow reproducing the results in this paper we provide all data in a zip archive. After expanding the archive users will find a directory Data with two subdirectories. Further information about the resource may be found in the file *readme.txt* which is located in the Data directory. A public GitHub repository which contains all data and code under a GPL v3 license can be accessed by following the link https://github.com/TW-Robotics/NT_BodyParts.
(ZIP)
