# Supplementary figures and images for: Identifying geographically differentiated features of Ethopian Nile tilapia (Oreochromis niloticus) morphology with machine learning (part 1 of 2)
Source: PLoS One. 2021 Apr 15;16(4):e0249593. doi: 10.1371/journal.pone.0249593 (PMC8049267; doi:10.1371/journal.pone.0249593)

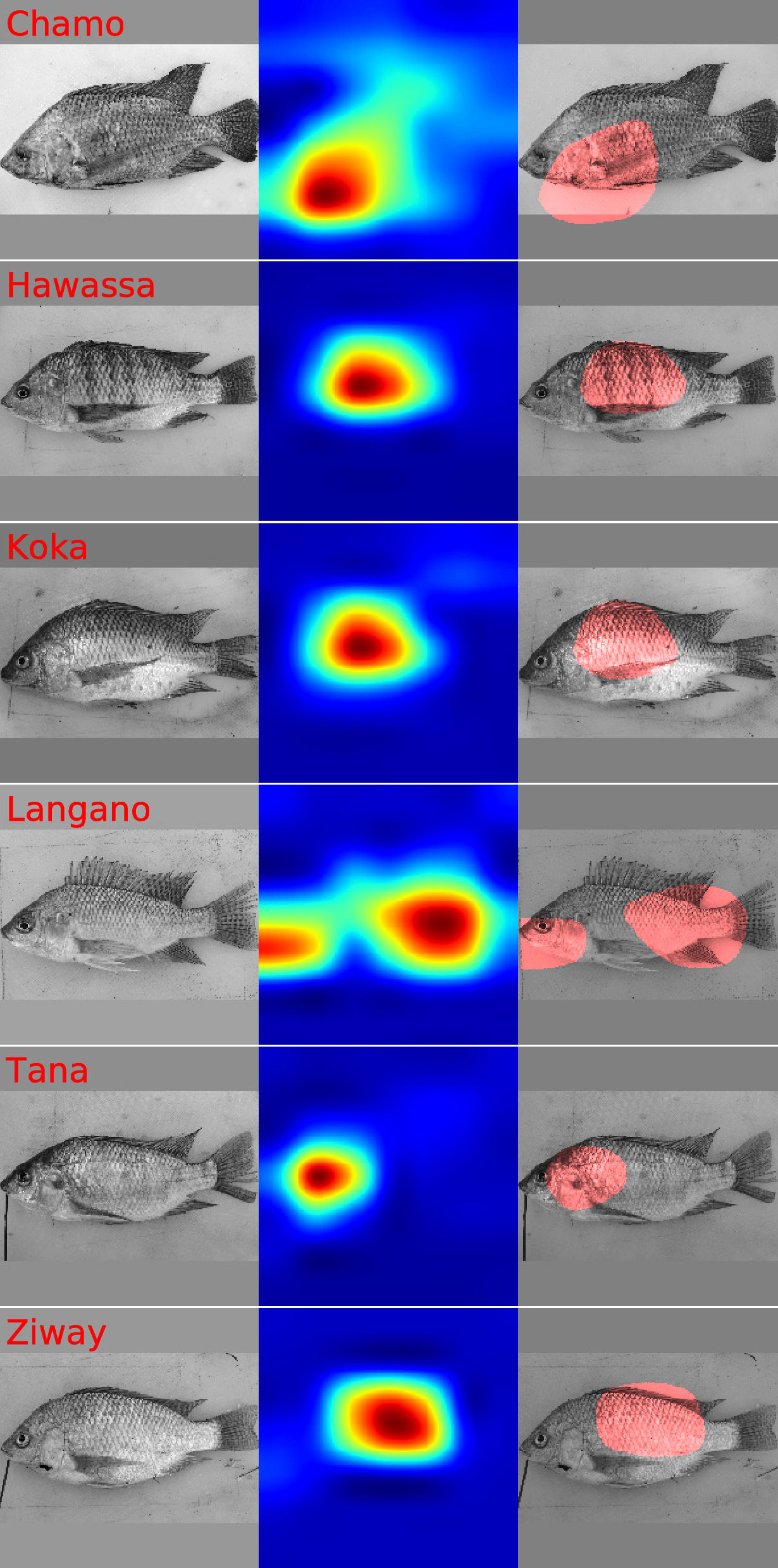

Supplement: S1 Fig — This figure illustrates fish images in column one, GRAD-CAM diagnostic plots in column two and fish images overlayed with a red colored GRAD-CAM derived significance mask in column three (p-val<0.001, calculation according to Eq (5)). Visualization are tagged by lake of origin and show samples which are highly representative for the respective lakes. While the samples of lakes Hawassa, Koka, Tana and Ziway raise no concern, the visualization of the samples for lakes Chamo and Langano suggest a significant dependency of predictions on the image background. (TIF) [file pone.0249593.s001.tif]

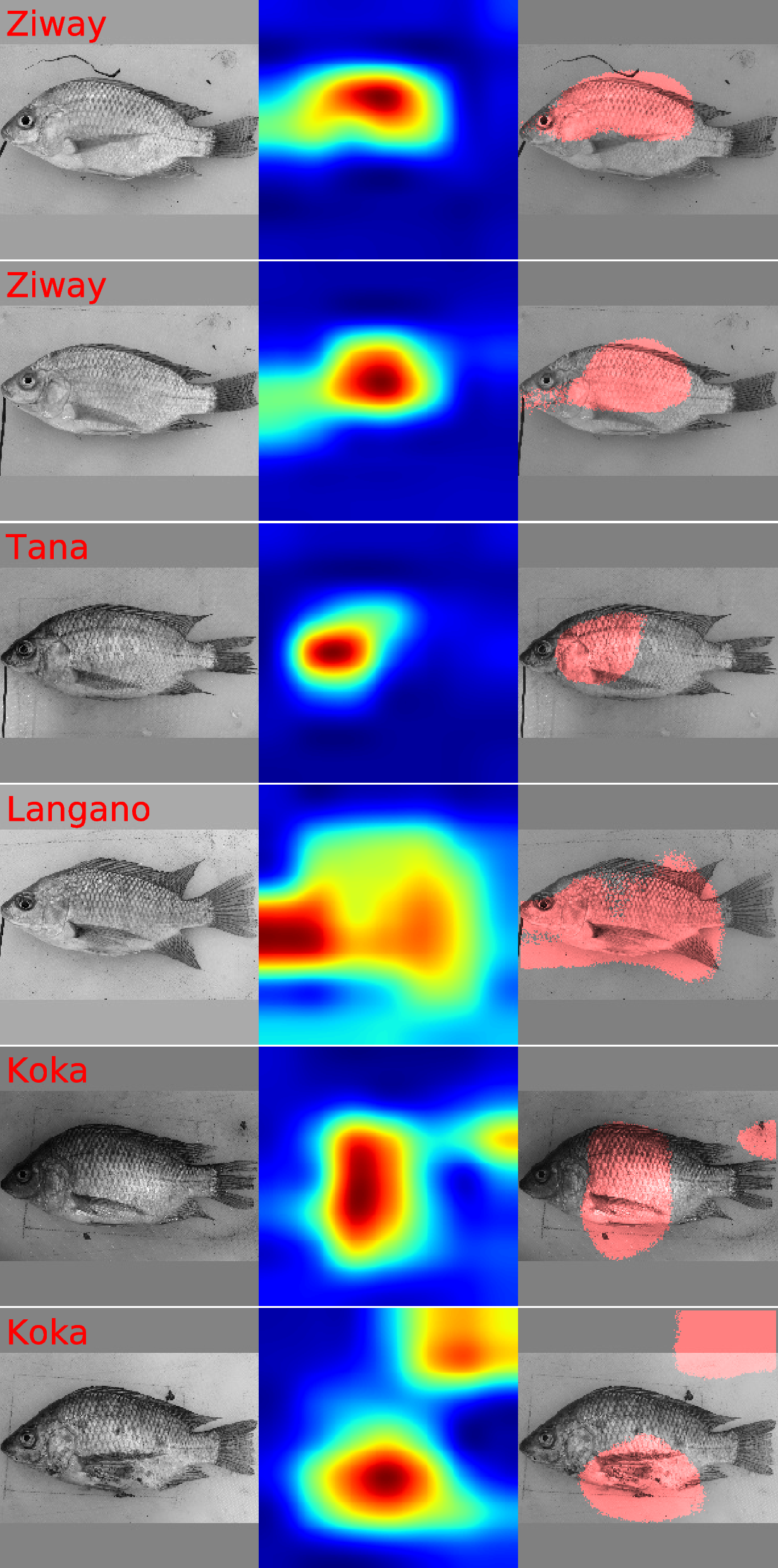

Supplement: S2 Fig — This figure illustrates fish images in column one, GRAD-CAM diagnostic plots in column two and fish images overlayed with a red colored GRAD-CAM derived significance mask in column three (p-val<0.001, calculation according to Eq (5)). Except for the lake Tana specimen all samples show that predictions are aided by contamination with technical artifacts. (TIF) [file pone.0249593.s002.tif]

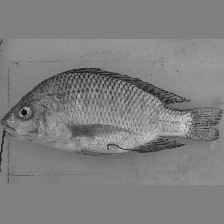

Supplement: S1 File — To allow reproducing the results in this paper we provide all data in a zip archive. After expanding the archive users will find a directory Data with two subdirectories. Further information about the resource may be found in the file readme.txt which is located in the Data directory. A public GitHub repository which contains all data and code under a GPL v3 license can be accessed by following the link https://github.com/TW-Robotics/NT_BodyParts. (ZIP) [file pone.0249593.s003.zip › Data/images/Ziway05.jpg]

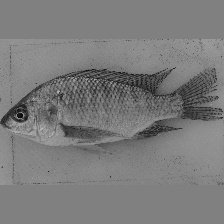

Supplement: S1 File — To allow reproducing the results in this paper we provide all data in a zip archive. After expanding the archive users will find a directory Data with two subdirectories. Further information about the resource may be found in the file readme.txt which is located in the Data directory. A public GitHub repository which contains all data and code under a GPL v3 license can be accessed by following the link https://github.com/TW-Robotics/NT_BodyParts. (ZIP) [file pone.0249593.s003.zip › Data/images/Langano23.jpg]

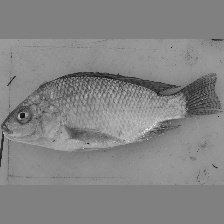

Supplement: S1 File — To allow reproducing the results in this paper we provide all data in a zip archive. After expanding the archive users will find a directory Data with two subdirectories. Further information about the resource may be found in the file readme.txt which is located in the Data directory. A public GitHub repository which contains all data and code under a GPL v3 license can be accessed by following the link https://github.com/TW-Robotics/NT_BodyParts. (ZIP) [file pone.0249593.s003.zip › Data/images/Ziway17.jpg]

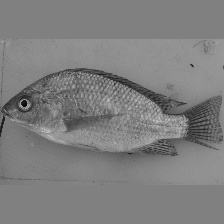

Supplement: S1 File — To allow reproducing the results in this paper we provide all data in a zip archive. After expanding the archive users will find a directory Data with two subdirectories. Further information about the resource may be found in the file readme.txt which is located in the Data directory. A public GitHub repository which contains all data and code under a GPL v3 license can be accessed by following the link https://github.com/TW-Robotics/NT_BodyParts. (ZIP) [file pone.0249593.s003.zip › Data/images/Ziway37.jpg]

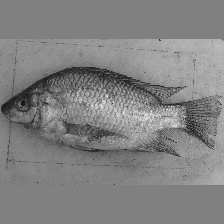

Supplement: S1 File — To allow reproducing the results in this paper we provide all data in a zip archive. After expanding the archive users will find a directory Data with two subdirectories. Further information about the resource may be found in the file readme.txt which is located in the Data directory. A public GitHub repository which contains all data and code under a GPL v3 license can be accessed by following the link https://github.com/TW-Robotics/NT_BodyParts. (ZIP) [file pone.0249593.s003.zip › Data/images/Koka15.jpg]

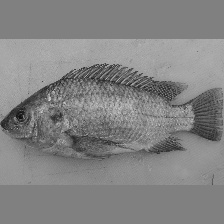

Supplement: S1 File — To allow reproducing the results in this paper we provide all data in a zip archive. After expanding the archive users will find a directory Data with two subdirectories. Further information about the resource may be found in the file readme.txt which is located in the Data directory. A public GitHub repository which contains all data and code under a GPL v3 license can be accessed by following the link https://github.com/TW-Robotics/NT_BodyParts. (ZIP) [file pone.0249593.s003.zip › Data/images/Tana11.jpg]

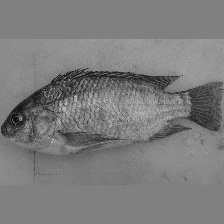

Supplement: S1 File — To allow reproducing the results in this paper we provide all data in a zip archive. After expanding the archive users will find a directory Data with two subdirectories. Further information about the resource may be found in the file readme.txt which is located in the Data directory. A public GitHub repository which contains all data and code under a GPL v3 license can be accessed by following the link https://github.com/TW-Robotics/NT_BodyParts. (ZIP) [file pone.0249593.s003.zip › Data/images/Tana32.jpg]

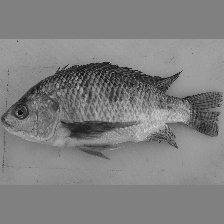

Supplement: S1 File — To allow reproducing the results in this paper we provide all data in a zip archive. After expanding the archive users will find a directory Data with two subdirectories. Further information about the resource may be found in the file readme.txt which is located in the Data directory. A public GitHub repository which contains all data and code under a GPL v3 license can be accessed by following the link https://github.com/TW-Robotics/NT_BodyParts. (ZIP) [file pone.0249593.s003.zip › Data/images/Hawassa07.jpg]

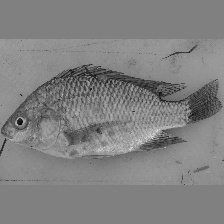

Supplement: S1 File — To allow reproducing the results in this paper we provide all data in a zip archive. After expanding the archive users will find a directory Data with two subdirectories. Further information about the resource may be found in the file readme.txt which is located in the Data directory. A public GitHub repository which contains all data and code under a GPL v3 license can be accessed by following the link https://github.com/TW-Robotics/NT_BodyParts. (ZIP) [file pone.0249593.s003.zip › Data/images/Ziway15.jpg]

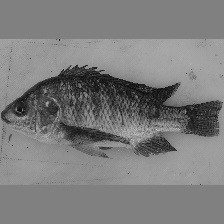

Supplement: S1 File — To allow reproducing the results in this paper we provide all data in a zip archive. After expanding the archive users will find a directory Data with two subdirectories. Further information about the resource may be found in the file readme.txt which is located in the Data directory. A public GitHub repository which contains all data and code under a GPL v3 license can be accessed by following the link https://github.com/TW-Robotics/NT_BodyParts. (ZIP) [file pone.0249593.s003.zip › Data/images/Hawassa25.jpg]

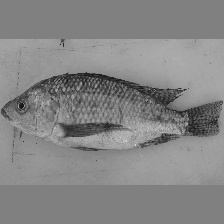

Supplement: S1 File — To allow reproducing the results in this paper we provide all data in a zip archive. After expanding the archive users will find a directory Data with two subdirectories. Further information about the resource may be found in the file readme.txt which is located in the Data directory. A public GitHub repository which contains all data and code under a GPL v3 license can be accessed by following the link https://github.com/TW-Robotics/NT_BodyParts. (ZIP) [file pone.0249593.s003.zip › Data/images/Hawassa24.jpg]

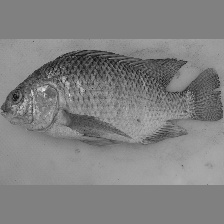

Supplement: S1 File — To allow reproducing the results in this paper we provide all data in a zip archive. After expanding the archive users will find a directory Data with two subdirectories. Further information about the resource may be found in the file readme.txt which is located in the Data directory. A public GitHub repository which contains all data and code under a GPL v3 license can be accessed by following the link https://github.com/TW-Robotics/NT_BodyParts. (ZIP) [file pone.0249593.s003.zip › Data/images/Chamo25.jpg]

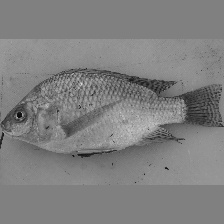

Supplement: S1 File — To allow reproducing the results in this paper we provide all data in a zip archive. After expanding the archive users will find a directory Data with two subdirectories. Further information about the resource may be found in the file readme.txt which is located in the Data directory. A public GitHub repository which contains all data and code under a GPL v3 license can be accessed by following the link https://github.com/TW-Robotics/NT_BodyParts. (ZIP) [file pone.0249593.s003.zip › Data/images/Ziway34.jpg]

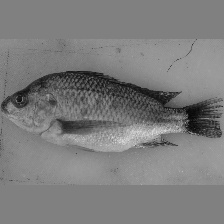

Supplement: S1 File — To allow reproducing the results in this paper we provide all data in a zip archive. After expanding the archive users will find a directory Data with two subdirectories. Further information about the resource may be found in the file readme.txt which is located in the Data directory. A public GitHub repository which contains all data and code under a GPL v3 license can be accessed by following the link https://github.com/TW-Robotics/NT_BodyParts. (ZIP) [file pone.0249593.s003.zip › Data/images/Hawassa38.jpg]

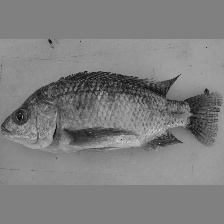

Supplement: S1 File — To allow reproducing the results in this paper we provide all data in a zip archive. After expanding the archive users will find a directory Data with two subdirectories. Further information about the resource may be found in the file readme.txt which is located in the Data directory. A public GitHub repository which contains all data and code under a GPL v3 license can be accessed by following the link https://github.com/TW-Robotics/NT_BodyParts. (ZIP) [file pone.0249593.s003.zip › Data/images/Hawassa16.jpg]

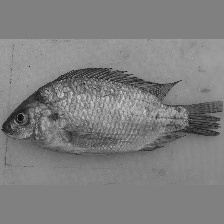

Supplement: S1 File — To allow reproducing the results in this paper we provide all data in a zip archive. After expanding the archive users will find a directory Data with two subdirectories. Further information about the resource may be found in the file readme.txt which is located in the Data directory. A public GitHub repository which contains all data and code under a GPL v3 license can be accessed by following the link https://github.com/TW-Robotics/NT_BodyParts. (ZIP) [file pone.0249593.s003.zip › Data/images/Tana12.jpg]

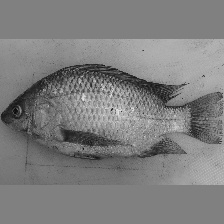

Supplement: S1 File — To allow reproducing the results in this paper we provide all data in a zip archive. After expanding the archive users will find a directory Data with two subdirectories. Further information about the resource may be found in the file readme.txt which is located in the Data directory. A public GitHub repository which contains all data and code under a GPL v3 license can be accessed by following the link https://github.com/TW-Robotics/NT_BodyParts. (ZIP) [file pone.0249593.s003.zip › Data/images/Koka12.jpg]

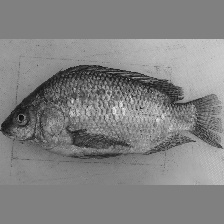

Supplement: S1 File — To allow reproducing the results in this paper we provide all data in a zip archive. After expanding the archive users will find a directory Data with two subdirectories. Further information about the resource may be found in the file readme.txt which is located in the Data directory. A public GitHub repository which contains all data and code under a GPL v3 license can be accessed by following the link https://github.com/TW-Robotics/NT_BodyParts. (ZIP) [file pone.0249593.s003.zip › Data/images/Koka05.jpg]

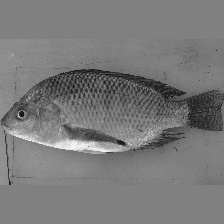

Supplement: S1 File — To allow reproducing the results in this paper we provide all data in a zip archive. After expanding the archive users will find a directory Data with two subdirectories. Further information about the resource may be found in the file readme.txt which is located in the Data directory. A public GitHub repository which contains all data and code under a GPL v3 license can be accessed by following the link https://github.com/TW-Robotics/NT_BodyParts. (ZIP) [file pone.0249593.s003.zip › Data/images/Ziway28.jpg]

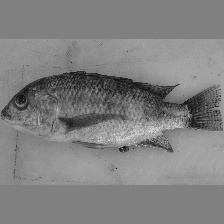

Supplement: S1 File — To allow reproducing the results in this paper we provide all data in a zip archive. After expanding the archive users will find a directory Data with two subdirectories. Further information about the resource may be found in the file readme.txt which is located in the Data directory. A public GitHub repository which contains all data and code under a GPL v3 license can be accessed by following the link https://github.com/TW-Robotics/NT_BodyParts. (ZIP) [file pone.0249593.s003.zip › Data/images/Hawassa37.jpg]

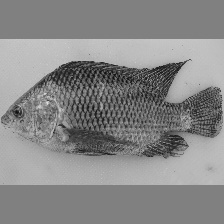

Supplement: S1 File — To allow reproducing the results in this paper we provide all data in a zip archive. After expanding the archive users will find a directory Data with two subdirectories. Further information about the resource may be found in the file readme.txt which is located in the Data directory. A public GitHub repository which contains all data and code under a GPL v3 license can be accessed by following the link https://github.com/TW-Robotics/NT_BodyParts. (ZIP) [file pone.0249593.s003.zip › Data/images/Chamo7.jpg]

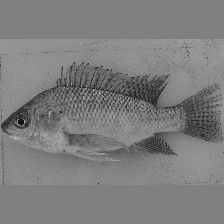

Supplement: S1 File — To allow reproducing the results in this paper we provide all data in a zip archive. After expanding the archive users will find a directory Data with two subdirectories. Further information about the resource may be found in the file readme.txt which is located in the Data directory. A public GitHub repository which contains all data and code under a GPL v3 license can be accessed by following the link https://github.com/TW-Robotics/NT_BodyParts. (ZIP) [file pone.0249593.s003.zip › Data/images/Langano26.jpg]

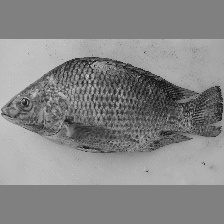

Supplement: S1 File — To allow reproducing the results in this paper we provide all data in a zip archive. After expanding the archive users will find a directory Data with two subdirectories. Further information about the resource may be found in the file readme.txt which is located in the Data directory. A public GitHub repository which contains all data and code under a GPL v3 license can be accessed by following the link https://github.com/TW-Robotics/NT_BodyParts. (ZIP) [file pone.0249593.s003.zip › Data/images/Chamo18.jpg]

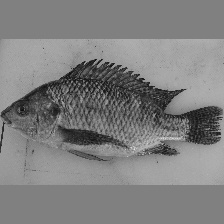

Supplement: S1 File — To allow reproducing the results in this paper we provide all data in a zip archive. After expanding the archive users will find a directory Data with two subdirectories. Further information about the resource may be found in the file readme.txt which is located in the Data directory. A public GitHub repository which contains all data and code under a GPL v3 license can be accessed by following the link https://github.com/TW-Robotics/NT_BodyParts. (ZIP) [file pone.0249593.s003.zip › Data/images/Hawassa31.jpg]

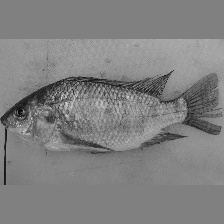

Supplement: S1 File — To allow reproducing the results in this paper we provide all data in a zip archive. After expanding the archive users will find a directory Data with two subdirectories. Further information about the resource may be found in the file readme.txt which is located in the Data directory. A public GitHub repository which contains all data and code under a GPL v3 license can be accessed by following the link https://github.com/TW-Robotics/NT_BodyParts. (ZIP) [file pone.0249593.s003.zip › Data/images/Tana38.jpg]

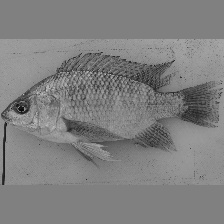

Supplement: S1 File — To allow reproducing the results in this paper we provide all data in a zip archive. After expanding the archive users will find a directory Data with two subdirectories. Further information about the resource may be found in the file readme.txt which is located in the Data directory. A public GitHub repository which contains all data and code under a GPL v3 license can be accessed by following the link https://github.com/TW-Robotics/NT_BodyParts. (ZIP) [file pone.0249593.s003.zip › Data/images/Langano21.jpg]

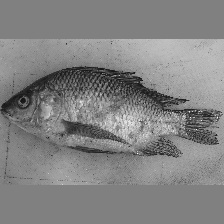

Supplement: S1 File — To allow reproducing the results in this paper we provide all data in a zip archive. After expanding the archive users will find a directory Data with two subdirectories. Further information about the resource may be found in the file readme.txt which is located in the Data directory. A public GitHub repository which contains all data and code under a GPL v3 license can be accessed by following the link https://github.com/TW-Robotics/NT_BodyParts. (ZIP) [file pone.0249593.s003.zip › Data/images/Koka23.jpg]

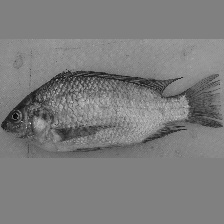

Supplement: S1 File — To allow reproducing the results in this paper we provide all data in a zip archive. After expanding the archive users will find a directory Data with two subdirectories. Further information about the resource may be found in the file readme.txt which is located in the Data directory. A public GitHub repository which contains all data and code under a GPL v3 license can be accessed by following the link https://github.com/TW-Robotics/NT_BodyParts. (ZIP) [file pone.0249593.s003.zip › Data/images/Tana18.jpg]

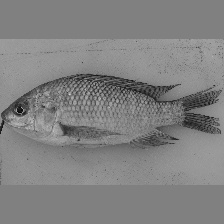

Supplement: S1 File — To allow reproducing the results in this paper we provide all data in a zip archive. After expanding the archive users will find a directory Data with two subdirectories. Further information about the resource may be found in the file readme.txt which is located in the Data directory. A public GitHub repository which contains all data and code under a GPL v3 license can be accessed by following the link https://github.com/TW-Robotics/NT_BodyParts. (ZIP) [file pone.0249593.s003.zip › Data/images/Langano19.jpg]

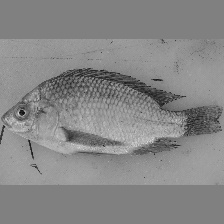

Supplement: S1 File — To allow reproducing the results in this paper we provide all data in a zip archive. After expanding the archive users will find a directory Data with two subdirectories. Further information about the resource may be found in the file readme.txt which is located in the Data directory. A public GitHub repository which contains all data and code under a GPL v3 license can be accessed by following the link https://github.com/TW-Robotics/NT_BodyParts. (ZIP) [file pone.0249593.s003.zip › Data/images/Ziway26.jpg]

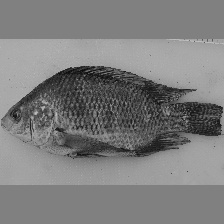

Supplement: S1 File — To allow reproducing the results in this paper we provide all data in a zip archive. After expanding the archive users will find a directory Data with two subdirectories. Further information about the resource may be found in the file readme.txt which is located in the Data directory. A public GitHub repository which contains all data and code under a GPL v3 license can be accessed by following the link https://github.com/TW-Robotics/NT_BodyParts. (ZIP) [file pone.0249593.s003.zip › Data/images/Chamo6.jpg]

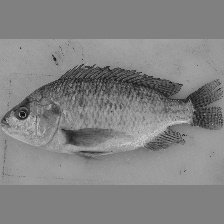

Supplement: S1 File — To allow reproducing the results in this paper we provide all data in a zip archive. After expanding the archive users will find a directory Data with two subdirectories. Further information about the resource may be found in the file readme.txt which is located in the Data directory. A public GitHub repository which contains all data and code under a GPL v3 license can be accessed by following the link https://github.com/TW-Robotics/NT_BodyParts. (ZIP) [file pone.0249593.s003.zip › Data/images/Hawassa19.jpg]

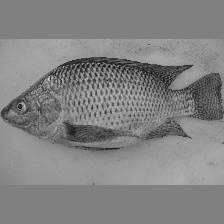

Supplement: S1 File — To allow reproducing the results in this paper we provide all data in a zip archive. After expanding the archive users will find a directory Data with two subdirectories. Further information about the resource may be found in the file readme.txt which is located in the Data directory. A public GitHub repository which contains all data and code under a GPL v3 license can be accessed by following the link https://github.com/TW-Robotics/NT_BodyParts. (ZIP) [file pone.0249593.s003.zip › Data/images/Chamo29.jpg]

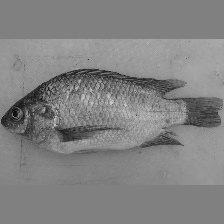

Supplement: S1 File — To allow reproducing the results in this paper we provide all data in a zip archive. After expanding the archive users will find a directory Data with two subdirectories. Further information about the resource may be found in the file readme.txt which is located in the Data directory. A public GitHub repository which contains all data and code under a GPL v3 license can be accessed by following the link https://github.com/TW-Robotics/NT_BodyParts. (ZIP) [file pone.0249593.s003.zip › Data/images/Tana14.jpg]

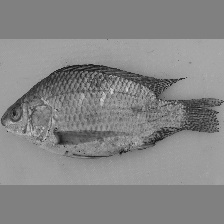

Supplement: S1 File — To allow reproducing the results in this paper we provide all data in a zip archive. After expanding the archive users will find a directory Data with two subdirectories. Further information about the resource may be found in the file readme.txt which is located in the Data directory. A public GitHub repository which contains all data and code under a GPL v3 license can be accessed by following the link https://github.com/TW-Robotics/NT_BodyParts. (ZIP) [file pone.0249593.s003.zip › Data/images/Chamo1.jpg]

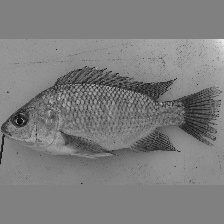

Supplement: S1 File — To allow reproducing the results in this paper we provide all data in a zip archive. After expanding the archive users will find a directory Data with two subdirectories. Further information about the resource may be found in the file readme.txt which is located in the Data directory. A public GitHub repository which contains all data and code under a GPL v3 license can be accessed by following the link https://github.com/TW-Robotics/NT_BodyParts. (ZIP) [file pone.0249593.s003.zip › Data/images/Lanagano20.jpg]

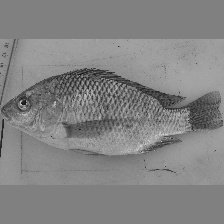

Supplement: S1 File — To allow reproducing the results in this paper we provide all data in a zip archive. After expanding the archive users will find a directory Data with two subdirectories. Further information about the resource may be found in the file readme.txt which is located in the Data directory. A public GitHub repository which contains all data and code under a GPL v3 license can be accessed by following the link https://github.com/TW-Robotics/NT_BodyParts. (ZIP) [file pone.0249593.s003.zip › Data/images/Ziway10.jpg]

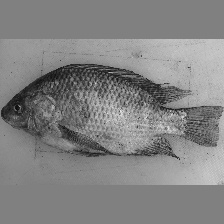

Supplement: S1 File — To allow reproducing the results in this paper we provide all data in a zip archive. After expanding the archive users will find a directory Data with two subdirectories. Further information about the resource may be found in the file readme.txt which is located in the Data directory. A public GitHub repository which contains all data and code under a GPL v3 license can be accessed by following the link https://github.com/TW-Robotics/NT_BodyParts. (ZIP) [file pone.0249593.s003.zip › Data/images/Koka10.jpg]

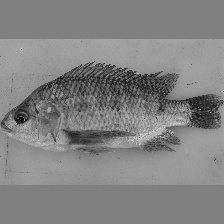

Supplement: S1 File — To allow reproducing the results in this paper we provide all data in a zip archive. After expanding the archive users will find a directory Data with two subdirectories. Further information about the resource may be found in the file readme.txt which is located in the Data directory. A public GitHub repository which contains all data and code under a GPL v3 license can be accessed by following the link https://github.com/TW-Robotics/NT_BodyParts. (ZIP) [file pone.0249593.s003.zip › Data/images/Hawassa13.jpg]

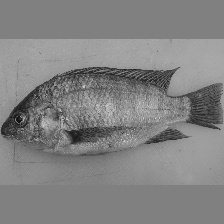

Supplement: S1 File — To allow reproducing the results in this paper we provide all data in a zip archive. After expanding the archive users will find a directory Data with two subdirectories. Further information about the resource may be found in the file readme.txt which is located in the Data directory. A public GitHub repository which contains all data and code under a GPL v3 license can be accessed by following the link https://github.com/TW-Robotics/NT_BodyParts. (ZIP) [file pone.0249593.s003.zip › Data/images/Tana02.jpg]

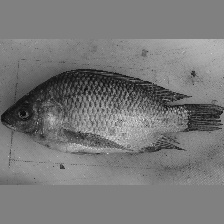

Supplement: S1 File — To allow reproducing the results in this paper we provide all data in a zip archive. After expanding the archive users will find a directory Data with two subdirectories. Further information about the resource may be found in the file readme.txt which is located in the Data directory. A public GitHub repository which contains all data and code under a GPL v3 license can be accessed by following the link https://github.com/TW-Robotics/NT_BodyParts. (ZIP) [file pone.0249593.s003.zip › Data/images/Koka28.jpg]

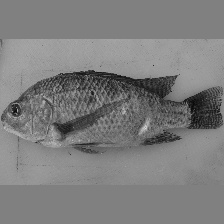

Supplement: S1 File — To allow reproducing the results in this paper we provide all data in a zip archive. After expanding the archive users will find a directory Data with two subdirectories. Further information about the resource may be found in the file readme.txt which is located in the Data directory. A public GitHub repository which contains all data and code under a GPL v3 license can be accessed by following the link https://github.com/TW-Robotics/NT_BodyParts. (ZIP) [file pone.0249593.s003.zip › Data/images/Hawassa01.jpg]

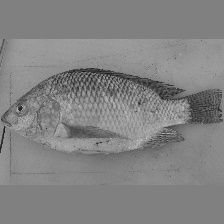

Supplement: S1 File — To allow reproducing the results in this paper we provide all data in a zip archive. After expanding the archive users will find a directory Data with two subdirectories. Further information about the resource may be found in the file readme.txt which is located in the Data directory. A public GitHub repository which contains all data and code under a GPL v3 license can be accessed by following the link https://github.com/TW-Robotics/NT_BodyParts. (ZIP) [file pone.0249593.s003.zip › Data/images/Ziway02.jpg]

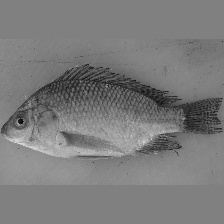

Supplement: S1 File — To allow reproducing the results in this paper we provide all data in a zip archive. After expanding the archive users will find a directory Data with two subdirectories. Further information about the resource may be found in the file readme.txt which is located in the Data directory. A public GitHub repository which contains all data and code under a GPL v3 license can be accessed by following the link https://github.com/TW-Robotics/NT_BodyParts. (ZIP) [file pone.0249593.s003.zip › Data/images/Ziway29.jpg]

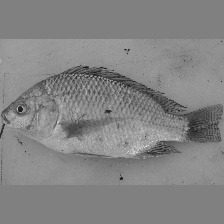

Supplement: S1 File — To allow reproducing the results in this paper we provide all data in a zip archive. After expanding the archive users will find a directory Data with two subdirectories. Further information about the resource may be found in the file readme.txt which is located in the Data directory. A public GitHub repository which contains all data and code under a GPL v3 license can be accessed by following the link https://github.com/TW-Robotics/NT_BodyParts. (ZIP) [file pone.0249593.s003.zip › Data/images/Ziway22.jpg]

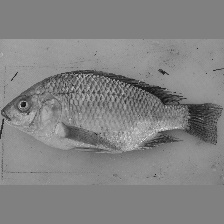

Supplement: S1 File — To allow reproducing the results in this paper we provide all data in a zip archive. After expanding the archive users will find a directory Data with two subdirectories. Further information about the resource may be found in the file readme.txt which is located in the Data directory. A public GitHub repository which contains all data and code under a GPL v3 license can be accessed by following the link https://github.com/TW-Robotics/NT_BodyParts. (ZIP) [file pone.0249593.s003.zip › Data/images/Ziway20.jpg]

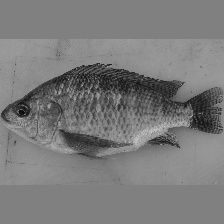

Supplement: S1 File — To allow reproducing the results in this paper we provide all data in a zip archive. After expanding the archive users will find a directory Data with two subdirectories. Further information about the resource may be found in the file readme.txt which is located in the Data directory. A public GitHub repository which contains all data and code under a GPL v3 license can be accessed by following the link https://github.com/TW-Robotics/NT_BodyParts. (ZIP) [file pone.0249593.s003.zip › Data/images/Hawassa32.jpg]

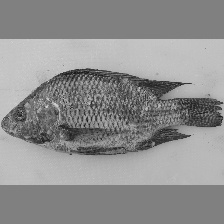

Supplement: S1 File — To allow reproducing the results in this paper we provide all data in a zip archive. After expanding the archive users will find a directory Data with two subdirectories. Further information about the resource may be found in the file readme.txt which is located in the Data directory. A public GitHub repository which contains all data and code under a GPL v3 license can be accessed by following the link https://github.com/TW-Robotics/NT_BodyParts. (ZIP) [file pone.0249593.s003.zip › Data/images/Chamo2.jpg]

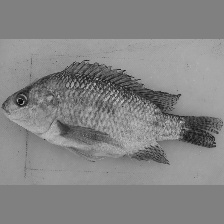

Supplement: S1 File — To allow reproducing the results in this paper we provide all data in a zip archive. After expanding the archive users will find a directory Data with two subdirectories. Further information about the resource may be found in the file readme.txt which is located in the Data directory. A public GitHub repository which contains all data and code under a GPL v3 license can be accessed by following the link https://github.com/TW-Robotics/NT_BodyParts. (ZIP) [file pone.0249593.s003.zip › Data/images/Hawassa04.jpg]

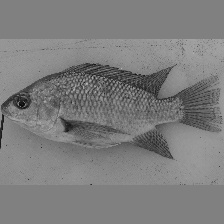

Supplement: S1 File — To allow reproducing the results in this paper we provide all data in a zip archive. After expanding the archive users will find a directory Data with two subdirectories. Further information about the resource may be found in the file readme.txt which is located in the Data directory. A public GitHub repository which contains all data and code under a GPL v3 license can be accessed by following the link https://github.com/TW-Robotics/NT_BodyParts. (ZIP) [file pone.0249593.s003.zip › Data/images/Langano25.jpg]

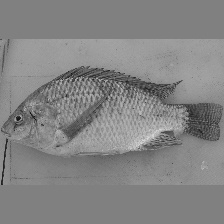

Supplement: S1 File — To allow reproducing the results in this paper we provide all data in a zip archive. After expanding the archive users will find a directory Data with two subdirectories. Further information about the resource may be found in the file readme.txt which is located in the Data directory. A public GitHub repository which contains all data and code under a GPL v3 license can be accessed by following the link https://github.com/TW-Robotics/NT_BodyParts. (ZIP) [file pone.0249593.s003.zip › Data/images/Ziway03.jpg]

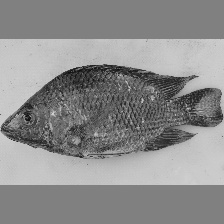

Supplement: S1 File — To allow reproducing the results in this paper we provide all data in a zip archive. After expanding the archive users will find a directory Data with two subdirectories. Further information about the resource may be found in the file readme.txt which is located in the Data directory. A public GitHub repository which contains all data and code under a GPL v3 license can be accessed by following the link https://github.com/TW-Robotics/NT_BodyParts. (ZIP) [file pone.0249593.s003.zip › Data/images/Chamo11.jpg]

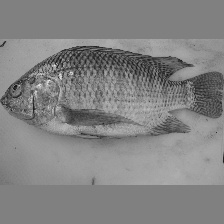

Supplement: S1 File — To allow reproducing the results in this paper we provide all data in a zip archive. After expanding the archive users will find a directory Data with two subdirectories. Further information about the resource may be found in the file readme.txt which is located in the Data directory. A public GitHub repository which contains all data and code under a GPL v3 license can be accessed by following the link https://github.com/TW-Robotics/NT_BodyParts. (ZIP) [file pone.0249593.s003.zip › Data/images/Chamo33.jpg]

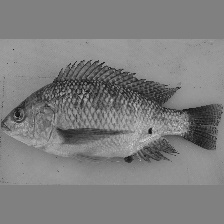

Supplement: S1 File — To allow reproducing the results in this paper we provide all data in a zip archive. After expanding the archive users will find a directory Data with two subdirectories. Further information about the resource may be found in the file readme.txt which is located in the Data directory. A public GitHub repository which contains all data and code under a GPL v3 license can be accessed by following the link https://github.com/TW-Robotics/NT_BodyParts. (ZIP) [file pone.0249593.s003.zip › Data/images/Hawassa09.jpg]

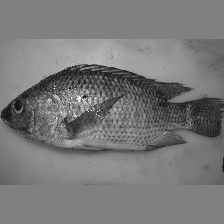

Supplement: S1 File — To allow reproducing the results in this paper we provide all data in a zip archive. After expanding the archive users will find a directory Data with two subdirectories. Further information about the resource may be found in the file readme.txt which is located in the Data directory. A public GitHub repository which contains all data and code under a GPL v3 license can be accessed by following the link https://github.com/TW-Robotics/NT_BodyParts. (ZIP) [file pone.0249593.s003.zip › Data/images/Chamo36.jpg]

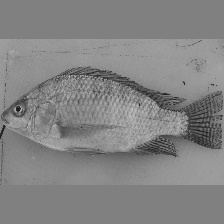

Supplement: S1 File — To allow reproducing the results in this paper we provide all data in a zip archive. After expanding the archive users will find a directory Data with two subdirectories. Further information about the resource may be found in the file readme.txt which is located in the Data directory. A public GitHub repository which contains all data and code under a GPL v3 license can be accessed by following the link https://github.com/TW-Robotics/NT_BodyParts. (ZIP) [file pone.0249593.s003.zip › Data/images/Ziway36.jpg]

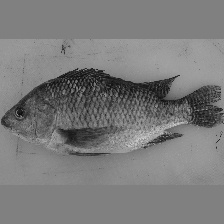

Supplement: S1 File — To allow reproducing the results in this paper we provide all data in a zip archive. After expanding the archive users will find a directory Data with two subdirectories. Further information about the resource may be found in the file readme.txt which is located in the Data directory. A public GitHub repository which contains all data and code under a GPL v3 license can be accessed by following the link https://github.com/TW-Robotics/NT_BodyParts. (ZIP) [file pone.0249593.s003.zip › Data/images/Hawassa20.jpg]

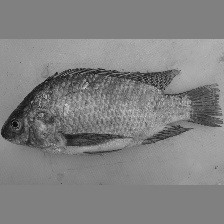

Supplement: S1 File — To allow reproducing the results in this paper we provide all data in a zip archive. After expanding the archive users will find a directory Data with two subdirectories. Further information about the resource may be found in the file readme.txt which is located in the Data directory. A public GitHub repository which contains all data and code under a GPL v3 license can be accessed by following the link https://github.com/TW-Robotics/NT_BodyParts. (ZIP) [file pone.0249593.s003.zip › Data/images/Tana15.jpg]

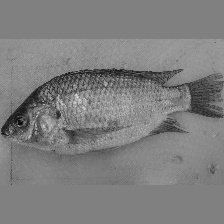

Supplement: S1 File — To allow reproducing the results in this paper we provide all data in a zip archive. After expanding the archive users will find a directory Data with two subdirectories. Further information about the resource may be found in the file readme.txt which is located in the Data directory. A public GitHub repository which contains all data and code under a GPL v3 license can be accessed by following the link https://github.com/TW-Robotics/NT_BodyParts. (ZIP) [file pone.0249593.s003.zip › Data/images/Tana28.jpg]

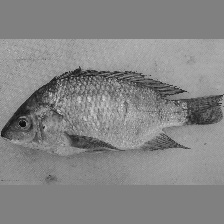

Supplement: S1 File — To allow reproducing the results in this paper we provide all data in a zip archive. After expanding the archive users will find a directory Data with two subdirectories. Further information about the resource may be found in the file readme.txt which is located in the Data directory. A public GitHub repository which contains all data and code under a GPL v3 license can be accessed by following the link https://github.com/TW-Robotics/NT_BodyParts. (ZIP) [file pone.0249593.s003.zip › Data/images/Tana37.jpg]

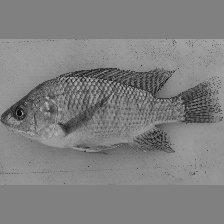

Supplement: S1 File — To allow reproducing the results in this paper we provide all data in a zip archive. After expanding the archive users will find a directory Data with two subdirectories. Further information about the resource may be found in the file readme.txt which is located in the Data directory. A public GitHub repository which contains all data and code under a GPL v3 license can be accessed by following the link https://github.com/TW-Robotics/NT_BodyParts. (ZIP) [file pone.0249593.s003.zip › Data/images/Langano22.jpg]

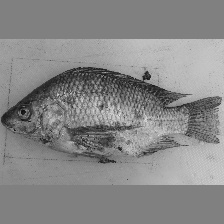

Supplement: S1 File — To allow reproducing the results in this paper we provide all data in a zip archive. After expanding the archive users will find a directory Data with two subdirectories. Further information about the resource may be found in the file readme.txt which is located in the Data directory. A public GitHub repository which contains all data and code under a GPL v3 license can be accessed by following the link https://github.com/TW-Robotics/NT_BodyParts. (ZIP) [file pone.0249593.s003.zip › Data/images/Koka03.jpg]

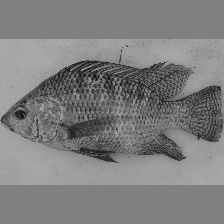

Supplement: S1 File — To allow reproducing the results in this paper we provide all data in a zip archive. After expanding the archive users will find a directory Data with two subdirectories. Further information about the resource may be found in the file readme.txt which is located in the Data directory. A public GitHub repository which contains all data and code under a GPL v3 license can be accessed by following the link https://github.com/TW-Robotics/NT_BodyParts. (ZIP) [file pone.0249593.s003.zip › Data/images/Chamo22.jpg]

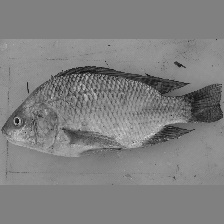

Supplement: S1 File — To allow reproducing the results in this paper we provide all data in a zip archive. After expanding the archive users will find a directory Data with two subdirectories. Further information about the resource may be found in the file readme.txt which is located in the Data directory. A public GitHub repository which contains all data and code under a GPL v3 license can be accessed by following the link https://github.com/TW-Robotics/NT_BodyParts. (ZIP) [file pone.0249593.s003.zip › Data/images/Ziway19.jpg]

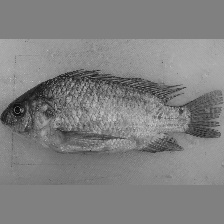

Supplement: S1 File — To allow reproducing the results in this paper we provide all data in a zip archive. After expanding the archive users will find a directory Data with two subdirectories. Further information about the resource may be found in the file readme.txt which is located in the Data directory. A public GitHub repository which contains all data and code under a GPL v3 license can be accessed by following the link https://github.com/TW-Robotics/NT_BodyParts. (ZIP) [file pone.0249593.s003.zip › Data/images/Tana21.jpg]

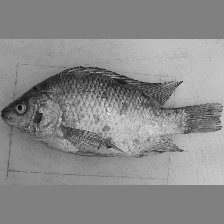

Supplement: S1 File — To allow reproducing the results in this paper we provide all data in a zip archive. After expanding the archive users will find a directory Data with two subdirectories. Further information about the resource may be found in the file readme.txt which is located in the Data directory. A public GitHub repository which contains all data and code under a GPL v3 license can be accessed by following the link https://github.com/TW-Robotics/NT_BodyParts. (ZIP) [file pone.0249593.s003.zip › Data/images/Koka01.jpg]

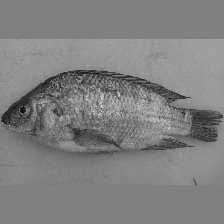

Supplement: S1 File — To allow reproducing the results in this paper we provide all data in a zip archive. After expanding the archive users will find a directory Data with two subdirectories. Further information about the resource may be found in the file readme.txt which is located in the Data directory. A public GitHub repository which contains all data and code under a GPL v3 license can be accessed by following the link https://github.com/TW-Robotics/NT_BodyParts. (ZIP) [file pone.0249593.s003.zip › Data/images/Tana05.jpg]

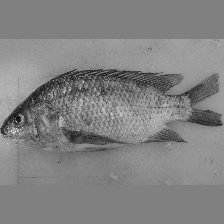

Supplement: S1 File — To allow reproducing the results in this paper we provide all data in a zip archive. After expanding the archive users will find a directory Data with two subdirectories. Further information about the resource may be found in the file readme.txt which is located in the Data directory. A public GitHub repository which contains all data and code under a GPL v3 license can be accessed by following the link https://github.com/TW-Robotics/NT_BodyParts. (ZIP) [file pone.0249593.s003.zip › Data/images/Tana23.jpg]

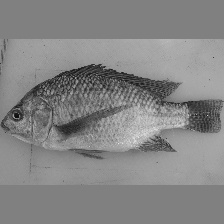

Supplement: S1 File — To allow reproducing the results in this paper we provide all data in a zip archive. After expanding the archive users will find a directory Data with two subdirectories. Further information about the resource may be found in the file readme.txt which is located in the Data directory. A public GitHub repository which contains all data and code under a GPL v3 license can be accessed by following the link https://github.com/TW-Robotics/NT_BodyParts. (ZIP) [file pone.0249593.s003.zip › Data/images/Hawassa03.jpg]

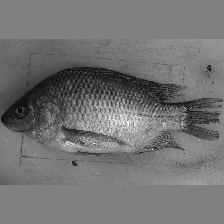

Supplement: S1 File — To allow reproducing the results in this paper we provide all data in a zip archive. After expanding the archive users will find a directory Data with two subdirectories. Further information about the resource may be found in the file readme.txt which is located in the Data directory. A public GitHub repository which contains all data and code under a GPL v3 license can be accessed by following the link https://github.com/TW-Robotics/NT_BodyParts. (ZIP) [file pone.0249593.s003.zip › Data/images/Koka27.jpg]

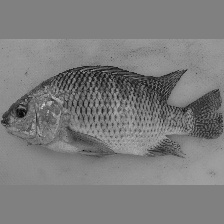

Supplement: S1 File — To allow reproducing the results in this paper we provide all data in a zip archive. After expanding the archive users will find a directory Data with two subdirectories. Further information about the resource may be found in the file readme.txt which is located in the Data directory. A public GitHub repository which contains all data and code under a GPL v3 license can be accessed by following the link https://github.com/TW-Robotics/NT_BodyParts. (ZIP) [file pone.0249593.s003.zip › Data/images/Chamo23.jpg]

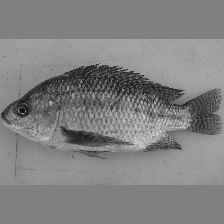

Supplement: S1 File — To allow reproducing the results in this paper we provide all data in a zip archive. After expanding the archive users will find a directory Data with two subdirectories. Further information about the resource may be found in the file readme.txt which is located in the Data directory. A public GitHub repository which contains all data and code under a GPL v3 license can be accessed by following the link https://github.com/TW-Robotics/NT_BodyParts. (ZIP) [file pone.0249593.s003.zip › Data/images/Hawassa05.jpg]

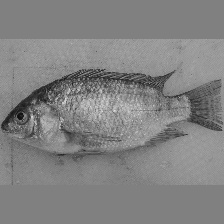

Supplement: S1 File — To allow reproducing the results in this paper we provide all data in a zip archive. After expanding the archive users will find a directory Data with two subdirectories. Further information about the resource may be found in the file readme.txt which is located in the Data directory. A public GitHub repository which contains all data and code under a GPL v3 license can be accessed by following the link https://github.com/TW-Robotics/NT_BodyParts. (ZIP) [file pone.0249593.s003.zip › Data/images/Tana31.jpg]

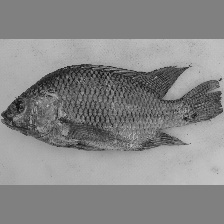

Supplement: S1 File — To allow reproducing the results in this paper we provide all data in a zip archive. After expanding the archive users will find a directory Data with two subdirectories. Further information about the resource may be found in the file readme.txt which is located in the Data directory. A public GitHub repository which contains all data and code under a GPL v3 license can be accessed by following the link https://github.com/TW-Robotics/NT_BodyParts. (ZIP) [file pone.0249593.s003.zip › Data/images/Chamo21.jpg]

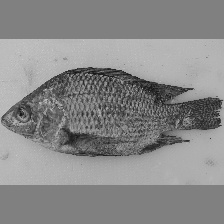

Supplement: S1 File — To allow reproducing the results in this paper we provide all data in a zip archive. After expanding the archive users will find a directory Data with two subdirectories. Further information about the resource may be found in the file readme.txt which is located in the Data directory. A public GitHub repository which contains all data and code under a GPL v3 license can be accessed by following the link https://github.com/TW-Robotics/NT_BodyParts. (ZIP) [file pone.0249593.s003.zip › Data/images/Chamo4.jpg]

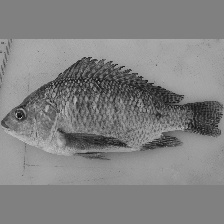

Supplement: S1 File — To allow reproducing the results in this paper we provide all data in a zip archive. After expanding the archive users will find a directory Data with two subdirectories. Further information about the resource may be found in the file readme.txt which is located in the Data directory. A public GitHub repository which contains all data and code under a GPL v3 license can be accessed by following the link https://github.com/TW-Robotics/NT_BodyParts. (ZIP) [file pone.0249593.s003.zip › Data/images/Hawassa02.jpg]

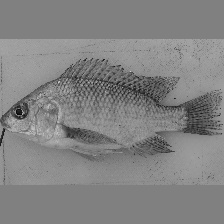

Supplement: S1 File — To allow reproducing the results in this paper we provide all data in a zip archive. After expanding the archive users will find a directory Data with two subdirectories. Further information about the resource may be found in the file readme.txt which is located in the Data directory. A public GitHub repository which contains all data and code under a GPL v3 license can be accessed by following the link https://github.com/TW-Robotics/NT_BodyParts. (ZIP) [file pone.0249593.s003.zip › Data/images/Langano11.jpg]

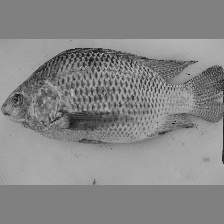

Supplement: S1 File — To allow reproducing the results in this paper we provide all data in a zip archive. After expanding the archive users will find a directory Data with two subdirectories. Further information about the resource may be found in the file readme.txt which is located in the Data directory. A public GitHub repository which contains all data and code under a GPL v3 license can be accessed by following the link https://github.com/TW-Robotics/NT_BodyParts. (ZIP) [file pone.0249593.s003.zip › Data/images/Chamo10.jpg]

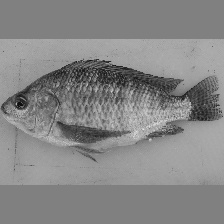

Supplement: S1 File — To allow reproducing the results in this paper we provide all data in a zip archive. After expanding the archive users will find a directory Data with two subdirectories. Further information about the resource may be found in the file readme.txt which is located in the Data directory. A public GitHub repository which contains all data and code under a GPL v3 license can be accessed by following the link https://github.com/TW-Robotics/NT_BodyParts. (ZIP) [file pone.0249593.s003.zip › Data/images/Hawassa08.jpg]

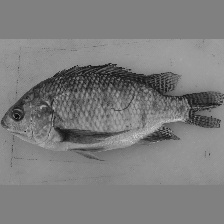

Supplement: S1 File — To allow reproducing the results in this paper we provide all data in a zip archive. After expanding the archive users will find a directory Data with two subdirectories. Further information about the resource may be found in the file readme.txt which is located in the Data directory. A public GitHub repository which contains all data and code under a GPL v3 license can be accessed by following the link https://github.com/TW-Robotics/NT_BodyParts. (ZIP) [file pone.0249593.s003.zip › Data/images/Hawassa18.jpg]

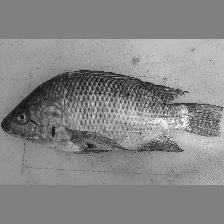

Supplement: S1 File — To allow reproducing the results in this paper we provide all data in a zip archive. After expanding the archive users will find a directory Data with two subdirectories. Further information about the resource may be found in the file readme.txt which is located in the Data directory. A public GitHub repository which contains all data and code under a GPL v3 license can be accessed by following the link https://github.com/TW-Robotics/NT_BodyParts. (ZIP) [file pone.0249593.s003.zip › Data/images/Koka22.jpg]

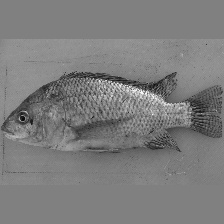

Supplement: S1 File — To allow reproducing the results in this paper we provide all data in a zip archive. After expanding the archive users will find a directory Data with two subdirectories. Further information about the resource may be found in the file readme.txt which is located in the Data directory. A public GitHub repository which contains all data and code under a GPL v3 license can be accessed by following the link https://github.com/TW-Robotics/NT_BodyParts. (ZIP) [file pone.0249593.s003.zip › Data/images/Ziway01.jpg]

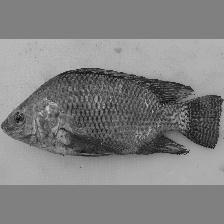

Supplement: S1 File — To allow reproducing the results in this paper we provide all data in a zip archive. After expanding the archive users will find a directory Data with two subdirectories. Further information about the resource may be found in the file readme.txt which is located in the Data directory. A public GitHub repository which contains all data and code under a GPL v3 license can be accessed by following the link https://github.com/TW-Robotics/NT_BodyParts. (ZIP) [file pone.0249593.s003.zip › Data/images/Chamo9.jpg]

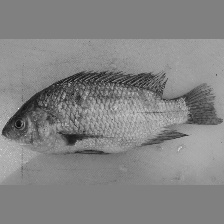

Supplement: S1 File — To allow reproducing the results in this paper we provide all data in a zip archive. After expanding the archive users will find a directory Data with two subdirectories. Further information about the resource may be found in the file readme.txt which is located in the Data directory. A public GitHub repository which contains all data and code under a GPL v3 license can be accessed by following the link https://github.com/TW-Robotics/NT_BodyParts. (ZIP) [file pone.0249593.s003.zip › Data/images/Tana26.jpg]

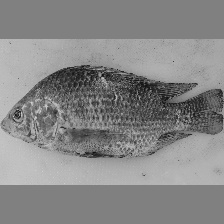

Supplement: S1 File — To allow reproducing the results in this paper we provide all data in a zip archive. After expanding the archive users will find a directory Data with two subdirectories. Further information about the resource may be found in the file readme.txt which is located in the Data directory. A public GitHub repository which contains all data and code under a GPL v3 license can be accessed by following the link https://github.com/TW-Robotics/NT_BodyParts. (ZIP) [file pone.0249593.s003.zip › Data/images/Chamo17.jpg]

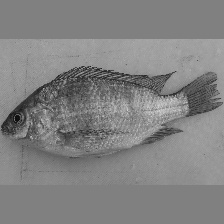

Supplement: S1 File — To allow reproducing the results in this paper we provide all data in a zip archive. After expanding the archive users will find a directory Data with two subdirectories. Further information about the resource may be found in the file readme.txt which is located in the Data directory. A public GitHub repository which contains all data and code under a GPL v3 license can be accessed by following the link https://github.com/TW-Robotics/NT_BodyParts. (ZIP) [file pone.0249593.s003.zip › Data/images/Tana06.jpg]

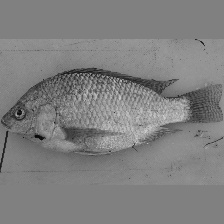

Supplement: S1 File — To allow reproducing the results in this paper we provide all data in a zip archive. After expanding the archive users will find a directory Data with two subdirectories. Further information about the resource may be found in the file readme.txt which is located in the Data directory. A public GitHub repository which contains all data and code under a GPL v3 license can be accessed by following the link https://github.com/TW-Robotics/NT_BodyParts. (ZIP) [file pone.0249593.s003.zip › Data/images/Ziway08.jpg]

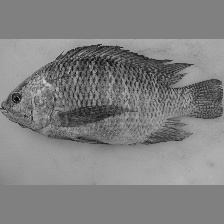

Supplement: S1 File — To allow reproducing the results in this paper we provide all data in a zip archive. After expanding the archive users will find a directory Data with two subdirectories. Further information about the resource may be found in the file readme.txt which is located in the Data directory. A public GitHub repository which contains all data and code under a GPL v3 license can be accessed by following the link https://github.com/TW-Robotics/NT_BodyParts. (ZIP) [file pone.0249593.s003.zip › Data/images/Chamo24.jpg]

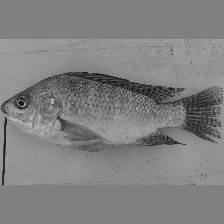

Supplement: S1 File — To allow reproducing the results in this paper we provide all data in a zip archive. After expanding the archive users will find a directory Data with two subdirectories. Further information about the resource may be found in the file readme.txt which is located in the Data directory. A public GitHub repository which contains all data and code under a GPL v3 license can be accessed by following the link https://github.com/TW-Robotics/NT_BodyParts. (ZIP) [file pone.0249593.s003.zip › Data/images/Langano08.jpg]

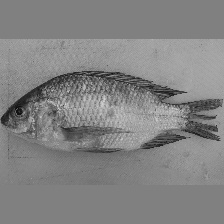

Supplement: S1 File — To allow reproducing the results in this paper we provide all data in a zip archive. After expanding the archive users will find a directory Data with two subdirectories. Further information about the resource may be found in the file readme.txt which is located in the Data directory. A public GitHub repository which contains all data and code under a GPL v3 license can be accessed by following the link https://github.com/TW-Robotics/NT_BodyParts. (ZIP) [file pone.0249593.s003.zip › Data/images/Tana27.jpg]

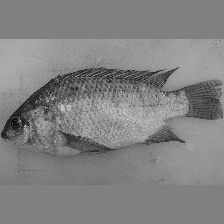

Supplement: S1 File — To allow reproducing the results in this paper we provide all data in a zip archive. After expanding the archive users will find a directory Data with two subdirectories. Further information about the resource may be found in the file readme.txt which is located in the Data directory. A public GitHub repository which contains all data and code under a GPL v3 license can be accessed by following the link https://github.com/TW-Robotics/NT_BodyParts. (ZIP) [file pone.0249593.s003.zip › Data/images/Tana20.jpg]

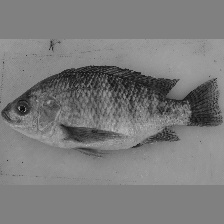

Supplement: S1 File — To allow reproducing the results in this paper we provide all data in a zip archive. After expanding the archive users will find a directory Data with two subdirectories. Further information about the resource may be found in the file readme.txt which is located in the Data directory. A public GitHub repository which contains all data and code under a GPL v3 license can be accessed by following the link https://github.com/TW-Robotics/NT_BodyParts. (ZIP) [file pone.0249593.s003.zip › Data/images/Hawassa22.jpg]

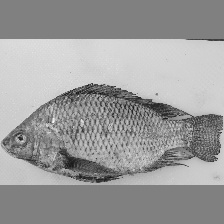

Supplement: S1 File — To allow reproducing the results in this paper we provide all data in a zip archive. After expanding the archive users will find a directory Data with two subdirectories. Further information about the resource may be found in the file readme.txt which is located in the Data directory. A public GitHub repository which contains all data and code under a GPL v3 license can be accessed by following the link https://github.com/TW-Robotics/NT_BodyParts. (ZIP) [file pone.0249593.s003.zip › Data/images/Chamo3.jpg]

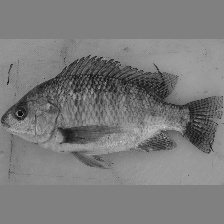

Supplement: S1 File — To allow reproducing the results in this paper we provide all data in a zip archive. After expanding the archive users will find a directory Data with two subdirectories. Further information about the resource may be found in the file readme.txt which is located in the Data directory. A public GitHub repository which contains all data and code under a GPL v3 license can be accessed by following the link https://github.com/TW-Robotics/NT_BodyParts. (ZIP) [file pone.0249593.s003.zip › Data/images/Hawassa35.jpg]

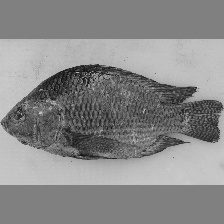

Supplement: S1 File — To allow reproducing the results in this paper we provide all data in a zip archive. After expanding the archive users will find a directory Data with two subdirectories. Further information about the resource may be found in the file readme.txt which is located in the Data directory. A public GitHub repository which contains all data and code under a GPL v3 license can be accessed by following the link https://github.com/TW-Robotics/NT_BodyParts. (ZIP) [file pone.0249593.s003.zip › Data/images/Chamo12.jpg]

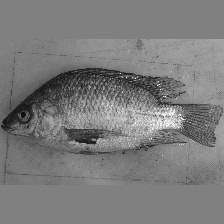

Supplement: S1 File — To allow reproducing the results in this paper we provide all data in a zip archive. After expanding the archive users will find a directory Data with two subdirectories. Further information about the resource may be found in the file readme.txt which is located in the Data directory. A public GitHub repository which contains all data and code under a GPL v3 license can be accessed by following the link https://github.com/TW-Robotics/NT_BodyParts. (ZIP) [file pone.0249593.s003.zip › Data/images/Koka20.jpg]

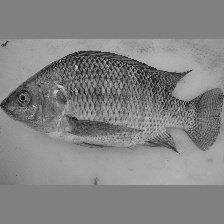

Supplement: S1 File — To allow reproducing the results in this paper we provide all data in a zip archive. After expanding the archive users will find a directory Data with two subdirectories. Further information about the resource may be found in the file readme.txt which is located in the Data directory. A public GitHub repository which contains all data and code under a GPL v3 license can be accessed by following the link https://github.com/TW-Robotics/NT_BodyParts. (ZIP) [file pone.0249593.s003.zip › Data/images/Chamo32.jpg]

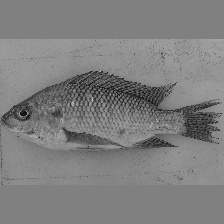

Supplement: S1 File — To allow reproducing the results in this paper we provide all data in a zip archive. After expanding the archive users will find a directory Data with two subdirectories. Further information about the resource may be found in the file readme.txt which is located in the Data directory. A public GitHub repository which contains all data and code under a GPL v3 license can be accessed by following the link https://github.com/TW-Robotics/NT_BodyParts. (ZIP) [file pone.0249593.s003.zip › Data/images/Langano10.jpg]
