# Supplementary figures and images for: Identifying geographically differentiated features of Ethopian Nile tilapia (Oreochromis niloticus) morphology with machine learning (part 2 of 2)
Source: PLoS One. 2021 Apr 15;16(4):e0249593. doi: 10.1371/journal.pone.0249593 (PMC8049267; doi:10.1371/journal.pone.0249593)

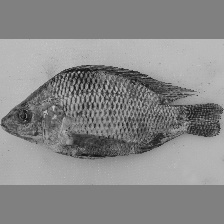

Supplement: S1 File — To allow reproducing the results in this paper we provide all data in a zip archive. After expanding the archive users will find a directory Data with two subdirectories. Further information about the resource may be found in the file readme.txt which is located in the Data directory. A public GitHub repository which contains all data and code under a GPL v3 license can be accessed by following the link https://github.com/TW-Robotics/NT_BodyParts. (ZIP) [file pone.0249593.s003.zip › Data/images/Chamo5.jpg]

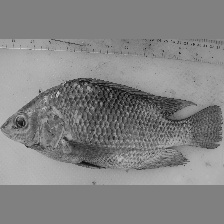

Supplement: S1 File — To allow reproducing the results in this paper we provide all data in a zip archive. After expanding the archive users will find a directory Data with two subdirectories. Further information about the resource may be found in the file readme.txt which is located in the Data directory. A public GitHub repository which contains all data and code under a GPL v3 license can be accessed by following the link https://github.com/TW-Robotics/NT_BodyParts. (ZIP) [file pone.0249593.s003.zip › Data/images/Chamo19.jpg]

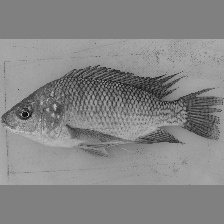

Supplement: S1 File — To allow reproducing the results in this paper we provide all data in a zip archive. After expanding the archive users will find a directory Data with two subdirectories. Further information about the resource may be found in the file readme.txt which is located in the Data directory. A public GitHub repository which contains all data and code under a GPL v3 license can be accessed by following the link https://github.com/TW-Robotics/NT_BodyParts. (ZIP) [file pone.0249593.s003.zip › Data/images/Langano01.jpg]

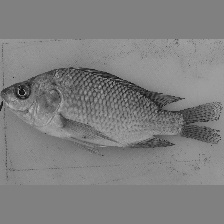

Supplement: S1 File — To allow reproducing the results in this paper we provide all data in a zip archive. After expanding the archive users will find a directory Data with two subdirectories. Further information about the resource may be found in the file readme.txt which is located in the Data directory. A public GitHub repository which contains all data and code under a GPL v3 license can be accessed by following the link https://github.com/TW-Robotics/NT_BodyParts. (ZIP) [file pone.0249593.s003.zip › Data/images/Langano06.jpg]

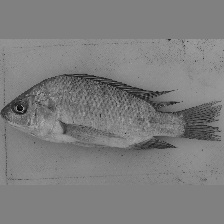

Supplement: S1 File — To allow reproducing the results in this paper we provide all data in a zip archive. After expanding the archive users will find a directory Data with two subdirectories. Further information about the resource may be found in the file readme.txt which is located in the Data directory. A public GitHub repository which contains all data and code under a GPL v3 license can be accessed by following the link https://github.com/TW-Robotics/NT_BodyParts. (ZIP) [file pone.0249593.s003.zip › Data/images/Langano15.jpg]

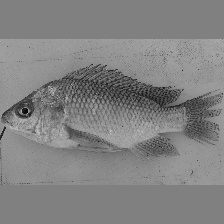

Supplement: S1 File — To allow reproducing the results in this paper we provide all data in a zip archive. After expanding the archive users will find a directory Data with two subdirectories. Further information about the resource may be found in the file readme.txt which is located in the Data directory. A public GitHub repository which contains all data and code under a GPL v3 license can be accessed by following the link https://github.com/TW-Robotics/NT_BodyParts. (ZIP) [file pone.0249593.s003.zip › Data/images/Langano07.jpg]

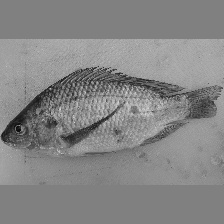

Supplement: S1 File — To allow reproducing the results in this paper we provide all data in a zip archive. After expanding the archive users will find a directory Data with two subdirectories. Further information about the resource may be found in the file readme.txt which is located in the Data directory. A public GitHub repository which contains all data and code under a GPL v3 license can be accessed by following the link https://github.com/TW-Robotics/NT_BodyParts. (ZIP) [file pone.0249593.s003.zip › Data/images/Tana30.jpg]

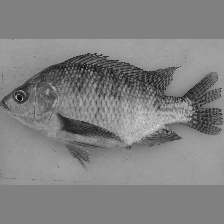

Supplement: S1 File — To allow reproducing the results in this paper we provide all data in a zip archive. After expanding the archive users will find a directory Data with two subdirectories. Further information about the resource may be found in the file readme.txt which is located in the Data directory. A public GitHub repository which contains all data and code under a GPL v3 license can be accessed by following the link https://github.com/TW-Robotics/NT_BodyParts. (ZIP) [file pone.0249593.s003.zip › Data/images/Hawassa11.jpg]

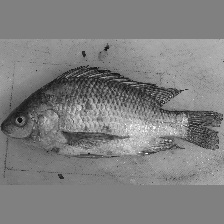

Supplement: S1 File — To allow reproducing the results in this paper we provide all data in a zip archive. After expanding the archive users will find a directory Data with two subdirectories. Further information about the resource may be found in the file readme.txt which is located in the Data directory. A public GitHub repository which contains all data and code under a GPL v3 license can be accessed by following the link https://github.com/TW-Robotics/NT_BodyParts. (ZIP) [file pone.0249593.s003.zip › Data/images/Koka29.jpg]

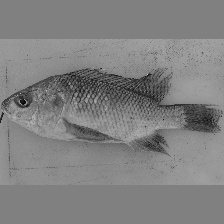

Supplement: S1 File — To allow reproducing the results in this paper we provide all data in a zip archive. After expanding the archive users will find a directory Data with two subdirectories. Further information about the resource may be found in the file readme.txt which is located in the Data directory. A public GitHub repository which contains all data and code under a GPL v3 license can be accessed by following the link https://github.com/TW-Robotics/NT_BodyParts. (ZIP) [file pone.0249593.s003.zip › Data/images/Langano02.jpg]

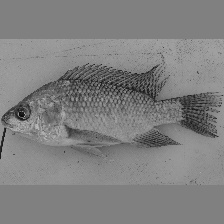

Supplement: S1 File — To allow reproducing the results in this paper we provide all data in a zip archive. After expanding the archive users will find a directory Data with two subdirectories. Further information about the resource may be found in the file readme.txt which is located in the Data directory. A public GitHub repository which contains all data and code under a GPL v3 license can be accessed by following the link https://github.com/TW-Robotics/NT_BodyParts. (ZIP) [file pone.0249593.s003.zip › Data/images/Langano13.jpg]

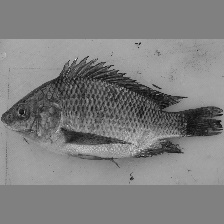

Supplement: S1 File — To allow reproducing the results in this paper we provide all data in a zip archive. After expanding the archive users will find a directory Data with two subdirectories. Further information about the resource may be found in the file readme.txt which is located in the Data directory. A public GitHub repository which contains all data and code under a GPL v3 license can be accessed by following the link https://github.com/TW-Robotics/NT_BodyParts. (ZIP) [file pone.0249593.s003.zip › Data/images/Ziway23.jpg]

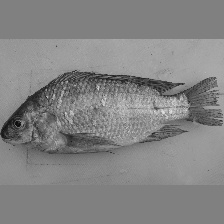

Supplement: S1 File — To allow reproducing the results in this paper we provide all data in a zip archive. After expanding the archive users will find a directory Data with two subdirectories. Further information about the resource may be found in the file readme.txt which is located in the Data directory. A public GitHub repository which contains all data and code under a GPL v3 license can be accessed by following the link https://github.com/TW-Robotics/NT_BodyParts. (ZIP) [file pone.0249593.s003.zip › Data/images/Tana03.jpg]

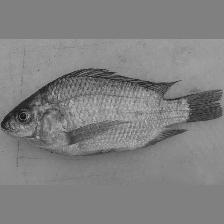

Supplement: S1 File — To allow reproducing the results in this paper we provide all data in a zip archive. After expanding the archive users will find a directory Data with two subdirectories. Further information about the resource may be found in the file readme.txt which is located in the Data directory. A public GitHub repository which contains all data and code under a GPL v3 license can be accessed by following the link https://github.com/TW-Robotics/NT_BodyParts. (ZIP) [file pone.0249593.s003.zip › Data/images/Tana10.jpg]

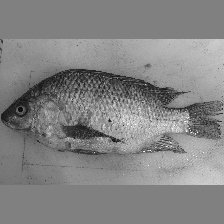

Supplement: S1 File — To allow reproducing the results in this paper we provide all data in a zip archive. After expanding the archive users will find a directory Data with two subdirectories. Further information about the resource may be found in the file readme.txt which is located in the Data directory. A public GitHub repository which contains all data and code under a GPL v3 license can be accessed by following the link https://github.com/TW-Robotics/NT_BodyParts. (ZIP) [file pone.0249593.s003.zip › Data/images/Koka26.jpg]

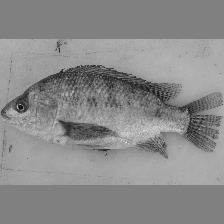

Supplement: S1 File — To allow reproducing the results in this paper we provide all data in a zip archive. After expanding the archive users will find a directory Data with two subdirectories. Further information about the resource may be found in the file readme.txt which is located in the Data directory. A public GitHub repository which contains all data and code under a GPL v3 license can be accessed by following the link https://github.com/TW-Robotics/NT_BodyParts. (ZIP) [file pone.0249593.s003.zip › Data/images/Hawassa28.jpg]

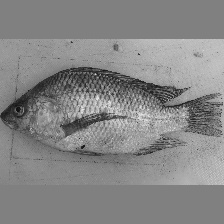

Supplement: S1 File — To allow reproducing the results in this paper we provide all data in a zip archive. After expanding the archive users will find a directory Data with two subdirectories. Further information about the resource may be found in the file readme.txt which is located in the Data directory. A public GitHub repository which contains all data and code under a GPL v3 license can be accessed by following the link https://github.com/TW-Robotics/NT_BodyParts. (ZIP) [file pone.0249593.s003.zip › Data/images/Koka14.jpg]

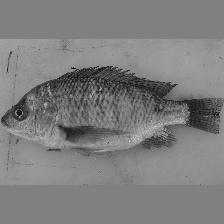

Supplement: S1 File — To allow reproducing the results in this paper we provide all data in a zip archive. After expanding the archive users will find a directory Data with two subdirectories. Further information about the resource may be found in the file readme.txt which is located in the Data directory. A public GitHub repository which contains all data and code under a GPL v3 license can be accessed by following the link https://github.com/TW-Robotics/NT_BodyParts. (ZIP) [file pone.0249593.s003.zip › Data/images/Hawassa34.jpg]

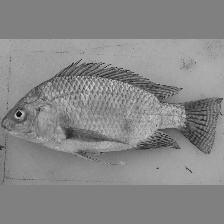

Supplement: S1 File — To allow reproducing the results in this paper we provide all data in a zip archive. After expanding the archive users will find a directory Data with two subdirectories. Further information about the resource may be found in the file readme.txt which is located in the Data directory. A public GitHub repository which contains all data and code under a GPL v3 license can be accessed by following the link https://github.com/TW-Robotics/NT_BodyParts. (ZIP) [file pone.0249593.s003.zip › Data/images/Ziway40.jpg]

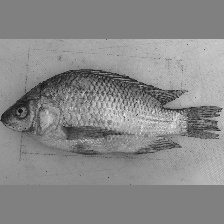

Supplement: S1 File — To allow reproducing the results in this paper we provide all data in a zip archive. After expanding the archive users will find a directory Data with two subdirectories. Further information about the resource may be found in the file readme.txt which is located in the Data directory. A public GitHub repository which contains all data and code under a GPL v3 license can be accessed by following the link https://github.com/TW-Robotics/NT_BodyParts. (ZIP) [file pone.0249593.s003.zip › Data/images/Koka16.jpg]

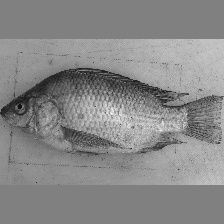

Supplement: S1 File — To allow reproducing the results in this paper we provide all data in a zip archive. After expanding the archive users will find a directory Data with two subdirectories. Further information about the resource may be found in the file readme.txt which is located in the Data directory. A public GitHub repository which contains all data and code under a GPL v3 license can be accessed by following the link https://github.com/TW-Robotics/NT_BodyParts. (ZIP) [file pone.0249593.s003.zip › Data/images/Koka17.jpg]

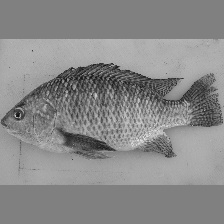

Supplement: S1 File — To allow reproducing the results in this paper we provide all data in a zip archive. After expanding the archive users will find a directory Data with two subdirectories. Further information about the resource may be found in the file readme.txt which is located in the Data directory. A public GitHub repository which contains all data and code under a GPL v3 license can be accessed by following the link https://github.com/TW-Robotics/NT_BodyParts. (ZIP) [file pone.0249593.s003.zip › Data/images/Hawassa10.jpg]

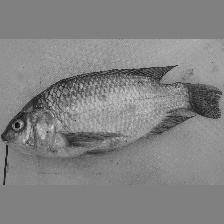

Supplement: S1 File — To allow reproducing the results in this paper we provide all data in a zip archive. After expanding the archive users will find a directory Data with two subdirectories. Further information about the resource may be found in the file readme.txt which is located in the Data directory. A public GitHub repository which contains all data and code under a GPL v3 license can be accessed by following the link https://github.com/TW-Robotics/NT_BodyParts. (ZIP) [file pone.0249593.s003.zip › Data/images/Tana36.jpg]

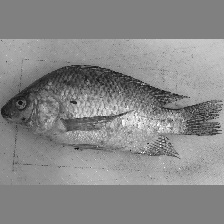

Supplement: S1 File — To allow reproducing the results in this paper we provide all data in a zip archive. After expanding the archive users will find a directory Data with two subdirectories. Further information about the resource may be found in the file readme.txt which is located in the Data directory. A public GitHub repository which contains all data and code under a GPL v3 license can be accessed by following the link https://github.com/TW-Robotics/NT_BodyParts. (ZIP) [file pone.0249593.s003.zip › Data/images/Koka24.jpg]

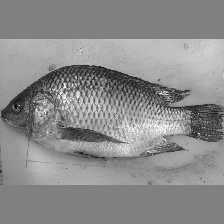

Supplement: S1 File — To allow reproducing the results in this paper we provide all data in a zip archive. After expanding the archive users will find a directory Data with two subdirectories. Further information about the resource may be found in the file readme.txt which is located in the Data directory. A public GitHub repository which contains all data and code under a GPL v3 license can be accessed by following the link https://github.com/TW-Robotics/NT_BodyParts. (ZIP) [file pone.0249593.s003.zip › Data/images/Koka31.jpg]

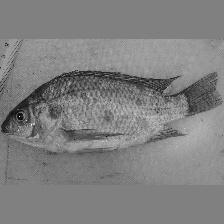

Supplement: S1 File — To allow reproducing the results in this paper we provide all data in a zip archive. After expanding the archive users will find a directory Data with two subdirectories. Further information about the resource may be found in the file readme.txt which is located in the Data directory. A public GitHub repository which contains all data and code under a GPL v3 license can be accessed by following the link https://github.com/TW-Robotics/NT_BodyParts. (ZIP) [file pone.0249593.s003.zip › Data/images/Tana01.jpg]

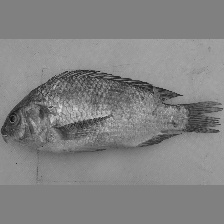

Supplement: S1 File — To allow reproducing the results in this paper we provide all data in a zip archive. After expanding the archive users will find a directory Data with two subdirectories. Further information about the resource may be found in the file readme.txt which is located in the Data directory. A public GitHub repository which contains all data and code under a GPL v3 license can be accessed by following the link https://github.com/TW-Robotics/NT_BodyParts. (ZIP) [file pone.0249593.s003.zip › Data/images/Tana04.jpg]

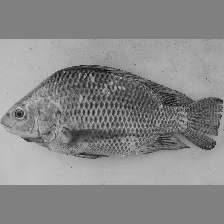

Supplement: S1 File — To allow reproducing the results in this paper we provide all data in a zip archive. After expanding the archive users will find a directory Data with two subdirectories. Further information about the resource may be found in the file readme.txt which is located in the Data directory. A public GitHub repository which contains all data and code under a GPL v3 license can be accessed by following the link https://github.com/TW-Robotics/NT_BodyParts. (ZIP) [file pone.0249593.s003.zip › Data/images/Chamo16.jpg]

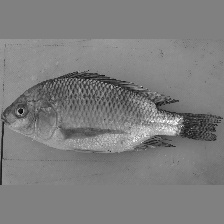

Supplement: S1 File — To allow reproducing the results in this paper we provide all data in a zip archive. After expanding the archive users will find a directory Data with two subdirectories. Further information about the resource may be found in the file readme.txt which is located in the Data directory. A public GitHub repository which contains all data and code under a GPL v3 license can be accessed by following the link https://github.com/TW-Robotics/NT_BodyParts. (ZIP) [file pone.0249593.s003.zip › Data/images/Ziway30.jpg]

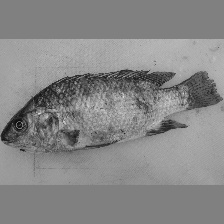

Supplement: S1 File — To allow reproducing the results in this paper we provide all data in a zip archive. After expanding the archive users will find a directory Data with two subdirectories. Further information about the resource may be found in the file readme.txt which is located in the Data directory. A public GitHub repository which contains all data and code under a GPL v3 license can be accessed by following the link https://github.com/TW-Robotics/NT_BodyParts. (ZIP) [file pone.0249593.s003.zip › Data/images/Tana35.jpg]

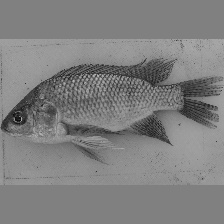

Supplement: S1 File — To allow reproducing the results in this paper we provide all data in a zip archive. After expanding the archive users will find a directory Data with two subdirectories. Further information about the resource may be found in the file readme.txt which is located in the Data directory. A public GitHub repository which contains all data and code under a GPL v3 license can be accessed by following the link https://github.com/TW-Robotics/NT_BodyParts. (ZIP) [file pone.0249593.s003.zip › Data/images/Langano24.jpg]

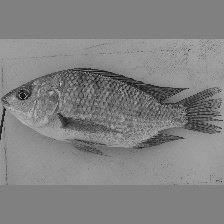

Supplement: S1 File — To allow reproducing the results in this paper we provide all data in a zip archive. After expanding the archive users will find a directory Data with two subdirectories. Further information about the resource may be found in the file readme.txt which is located in the Data directory. A public GitHub repository which contains all data and code under a GPL v3 license can be accessed by following the link https://github.com/TW-Robotics/NT_BodyParts. (ZIP) [file pone.0249593.s003.zip › Data/images/Langano09.jpg]

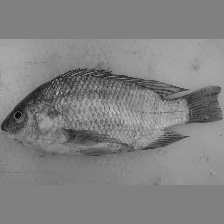

Supplement: S1 File — To allow reproducing the results in this paper we provide all data in a zip archive. After expanding the archive users will find a directory Data with two subdirectories. Further information about the resource may be found in the file readme.txt which is located in the Data directory. A public GitHub repository which contains all data and code under a GPL v3 license can be accessed by following the link https://github.com/TW-Robotics/NT_BodyParts. (ZIP) [file pone.0249593.s003.zip › Data/images/Tana22.jpg]

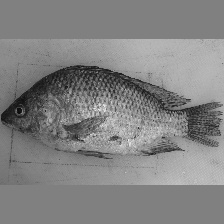

Supplement: S1 File — To allow reproducing the results in this paper we provide all data in a zip archive. After expanding the archive users will find a directory Data with two subdirectories. Further information about the resource may be found in the file readme.txt which is located in the Data directory. A public GitHub repository which contains all data and code under a GPL v3 license can be accessed by following the link https://github.com/TW-Robotics/NT_BodyParts. (ZIP) [file pone.0249593.s003.zip › Data/images/Koka04.jpg]

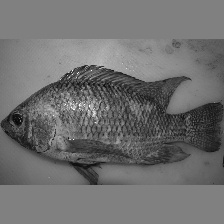

Supplement: S1 File — To allow reproducing the results in this paper we provide all data in a zip archive. After expanding the archive users will find a directory Data with two subdirectories. Further information about the resource may be found in the file readme.txt which is located in the Data directory. A public GitHub repository which contains all data and code under a GPL v3 license can be accessed by following the link https://github.com/TW-Robotics/NT_BodyParts. (ZIP) [file pone.0249593.s003.zip › Data/images/Chamo34.jpg]

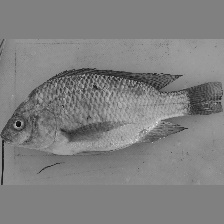

Supplement: S1 File — To allow reproducing the results in this paper we provide all data in a zip archive. After expanding the archive users will find a directory Data with two subdirectories. Further information about the resource may be found in the file readme.txt which is located in the Data directory. A public GitHub repository which contains all data and code under a GPL v3 license can be accessed by following the link https://github.com/TW-Robotics/NT_BodyParts. (ZIP) [file pone.0249593.s003.zip › Data/images/Ziway11.jpg]

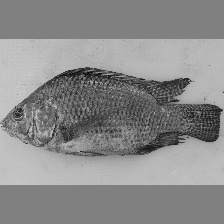

Supplement: S1 File — To allow reproducing the results in this paper we provide all data in a zip archive. After expanding the archive users will find a directory Data with two subdirectories. Further information about the resource may be found in the file readme.txt which is located in the Data directory. A public GitHub repository which contains all data and code under a GPL v3 license can be accessed by following the link https://github.com/TW-Robotics/NT_BodyParts. (ZIP) [file pone.0249593.s003.zip › Data/images/Chamo13.jpg]

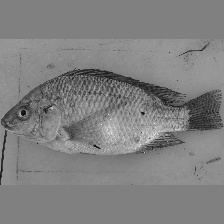

Supplement: S1 File — To allow reproducing the results in this paper we provide all data in a zip archive. After expanding the archive users will find a directory Data with two subdirectories. Further information about the resource may be found in the file readme.txt which is located in the Data directory. A public GitHub repository which contains all data and code under a GPL v3 license can be accessed by following the link https://github.com/TW-Robotics/NT_BodyParts. (ZIP) [file pone.0249593.s003.zip › Data/images/Ziway16.jpg]

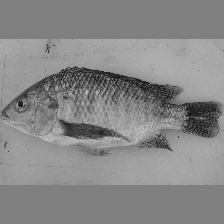

Supplement: S1 File — To allow reproducing the results in this paper we provide all data in a zip archive. After expanding the archive users will find a directory Data with two subdirectories. Further information about the resource may be found in the file readme.txt which is located in the Data directory. A public GitHub repository which contains all data and code under a GPL v3 license can be accessed by following the link https://github.com/TW-Robotics/NT_BodyParts. (ZIP) [file pone.0249593.s003.zip › Data/images/Hawassa23.jpg]

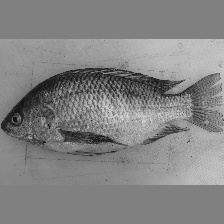

Supplement: S1 File — To allow reproducing the results in this paper we provide all data in a zip archive. After expanding the archive users will find a directory Data with two subdirectories. Further information about the resource may be found in the file readme.txt which is located in the Data directory. A public GitHub repository which contains all data and code under a GPL v3 license can be accessed by following the link https://github.com/TW-Robotics/NT_BodyParts. (ZIP) [file pone.0249593.s003.zip › Data/images/Koka11.jpg]

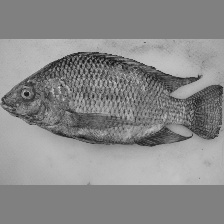

Supplement: S1 File — To allow reproducing the results in this paper we provide all data in a zip archive. After expanding the archive users will find a directory Data with two subdirectories. Further information about the resource may be found in the file readme.txt which is located in the Data directory. A public GitHub repository which contains all data and code under a GPL v3 license can be accessed by following the link https://github.com/TW-Robotics/NT_BodyParts. (ZIP) [file pone.0249593.s003.zip › Data/images/Chamo30.jpg]

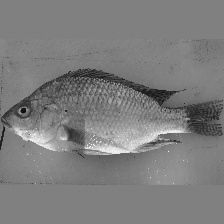

Supplement: S1 File — To allow reproducing the results in this paper we provide all data in a zip archive. After expanding the archive users will find a directory Data with two subdirectories. Further information about the resource may be found in the file readme.txt which is located in the Data directory. A public GitHub repository which contains all data and code under a GPL v3 license can be accessed by following the link https://github.com/TW-Robotics/NT_BodyParts. (ZIP) [file pone.0249593.s003.zip › Data/images/Ziway33.jpg]

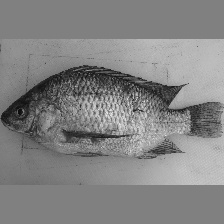

Supplement: S1 File — To allow reproducing the results in this paper we provide all data in a zip archive. After expanding the archive users will find a directory Data with two subdirectories. Further information about the resource may be found in the file readme.txt which is located in the Data directory. A public GitHub repository which contains all data and code under a GPL v3 license can be accessed by following the link https://github.com/TW-Robotics/NT_BodyParts. (ZIP) [file pone.0249593.s003.zip › Data/images/Koka02.jpg]

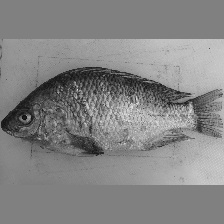

Supplement: S1 File — To allow reproducing the results in this paper we provide all data in a zip archive. After expanding the archive users will find a directory Data with two subdirectories. Further information about the resource may be found in the file readme.txt which is located in the Data directory. A public GitHub repository which contains all data and code under a GPL v3 license can be accessed by following the link https://github.com/TW-Robotics/NT_BodyParts. (ZIP) [file pone.0249593.s003.zip › Data/images/Koka09.jpg]

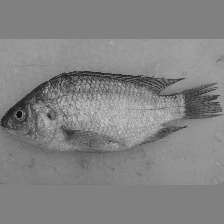

Supplement: S1 File — To allow reproducing the results in this paper we provide all data in a zip archive. After expanding the archive users will find a directory Data with two subdirectories. Further information about the resource may be found in the file readme.txt which is located in the Data directory. A public GitHub repository which contains all data and code under a GPL v3 license can be accessed by following the link https://github.com/TW-Robotics/NT_BodyParts. (ZIP) [file pone.0249593.s003.zip › Data/images/Tana17.jpg]

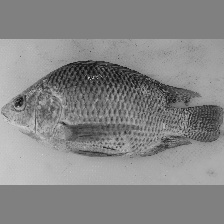

Supplement: S1 File — To allow reproducing the results in this paper we provide all data in a zip archive. After expanding the archive users will find a directory Data with two subdirectories. Further information about the resource may be found in the file readme.txt which is located in the Data directory. A public GitHub repository which contains all data and code under a GPL v3 license can be accessed by following the link https://github.com/TW-Robotics/NT_BodyParts. (ZIP) [file pone.0249593.s003.zip › Data/images/Chamo15.jpg]

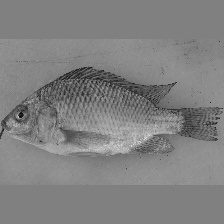

Supplement: S1 File — To allow reproducing the results in this paper we provide all data in a zip archive. After expanding the archive users will find a directory Data with two subdirectories. Further information about the resource may be found in the file readme.txt which is located in the Data directory. A public GitHub repository which contains all data and code under a GPL v3 license can be accessed by following the link https://github.com/TW-Robotics/NT_BodyParts. (ZIP) [file pone.0249593.s003.zip › Data/images/Ziway27.jpg]

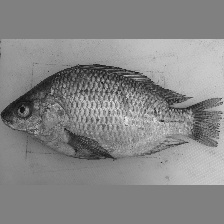

Supplement: S1 File — To allow reproducing the results in this paper we provide all data in a zip archive. After expanding the archive users will find a directory Data with two subdirectories. Further information about the resource may be found in the file readme.txt which is located in the Data directory. A public GitHub repository which contains all data and code under a GPL v3 license can be accessed by following the link https://github.com/TW-Robotics/NT_BodyParts. (ZIP) [file pone.0249593.s003.zip › Data/images/Koka07.jpg]

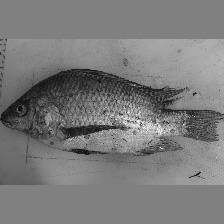

Supplement: S1 File — To allow reproducing the results in this paper we provide all data in a zip archive. After expanding the archive users will find a directory Data with two subdirectories. Further information about the resource may be found in the file readme.txt which is located in the Data directory. A public GitHub repository which contains all data and code under a GPL v3 license can be accessed by following the link https://github.com/TW-Robotics/NT_BodyParts. (ZIP) [file pone.0249593.s003.zip › Data/images/Koka19.jpg]

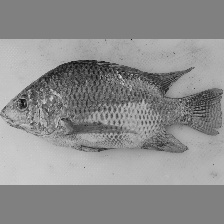

Supplement: S1 File — To allow reproducing the results in this paper we provide all data in a zip archive. After expanding the archive users will find a directory Data with two subdirectories. Further information about the resource may be found in the file readme.txt which is located in the Data directory. A public GitHub repository which contains all data and code under a GPL v3 license can be accessed by following the link https://github.com/TW-Robotics/NT_BodyParts. (ZIP) [file pone.0249593.s003.zip › Data/images/Chamo14.jpg]

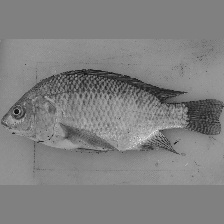

Supplement: S1 File — To allow reproducing the results in this paper we provide all data in a zip archive. After expanding the archive users will find a directory Data with two subdirectories. Further information about the resource may be found in the file readme.txt which is located in the Data directory. A public GitHub repository which contains all data and code under a GPL v3 license can be accessed by following the link https://github.com/TW-Robotics/NT_BodyParts. (ZIP) [file pone.0249593.s003.zip › Data/images/Ziway14.jpg]

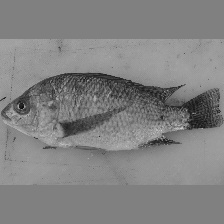

Supplement: S1 File — To allow reproducing the results in this paper we provide all data in a zip archive. After expanding the archive users will find a directory Data with two subdirectories. Further information about the resource may be found in the file readme.txt which is located in the Data directory. A public GitHub repository which contains all data and code under a GPL v3 license can be accessed by following the link https://github.com/TW-Robotics/NT_BodyParts. (ZIP) [file pone.0249593.s003.zip › Data/images/Hawassa33.jpg]

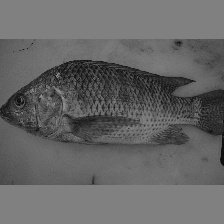

Supplement: S1 File — To allow reproducing the results in this paper we provide all data in a zip archive. After expanding the archive users will find a directory Data with two subdirectories. Further information about the resource may be found in the file readme.txt which is located in the Data directory. A public GitHub repository which contains all data and code under a GPL v3 license can be accessed by following the link https://github.com/TW-Robotics/NT_BodyParts. (ZIP) [file pone.0249593.s003.zip › Data/images/Chamo35.jpg]

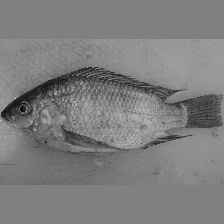

Supplement: S1 File — To allow reproducing the results in this paper we provide all data in a zip archive. After expanding the archive users will find a directory Data with two subdirectories. Further information about the resource may be found in the file readme.txt which is located in the Data directory. A public GitHub repository which contains all data and code under a GPL v3 license can be accessed by following the link https://github.com/TW-Robotics/NT_BodyParts. (ZIP) [file pone.0249593.s003.zip › Data/images/Tana25.jpg]

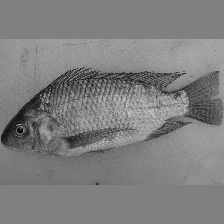

Supplement: S1 File — To allow reproducing the results in this paper we provide all data in a zip archive. After expanding the archive users will find a directory Data with two subdirectories. Further information about the resource may be found in the file readme.txt which is located in the Data directory. A public GitHub repository which contains all data and code under a GPL v3 license can be accessed by following the link https://github.com/TW-Robotics/NT_BodyParts. (ZIP) [file pone.0249593.s003.zip › Data/images/Tana16.jpg]

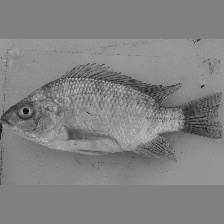

Supplement: S1 File — To allow reproducing the results in this paper we provide all data in a zip archive. After expanding the archive users will find a directory Data with two subdirectories. Further information about the resource may be found in the file readme.txt which is located in the Data directory. A public GitHub repository which contains all data and code under a GPL v3 license can be accessed by following the link https://github.com/TW-Robotics/NT_BodyParts. (ZIP) [file pone.0249593.s003.zip › Data/images/Ziway39.jpg]

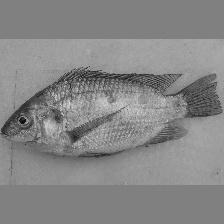

Supplement: S1 File — To allow reproducing the results in this paper we provide all data in a zip archive. After expanding the archive users will find a directory Data with two subdirectories. Further information about the resource may be found in the file readme.txt which is located in the Data directory. A public GitHub repository which contains all data and code under a GPL v3 license can be accessed by following the link https://github.com/TW-Robotics/NT_BodyParts. (ZIP) [file pone.0249593.s003.zip › Data/images/Tana08.jpg]

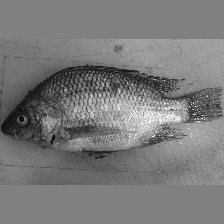

Supplement: S1 File — To allow reproducing the results in this paper we provide all data in a zip archive. After expanding the archive users will find a directory Data with two subdirectories. Further information about the resource may be found in the file readme.txt which is located in the Data directory. A public GitHub repository which contains all data and code under a GPL v3 license can be accessed by following the link https://github.com/TW-Robotics/NT_BodyParts. (ZIP) [file pone.0249593.s003.zip › Data/images/Koka21.jpg]

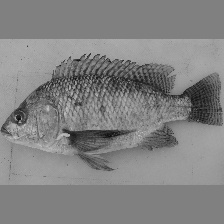

Supplement: S1 File — To allow reproducing the results in this paper we provide all data in a zip archive. After expanding the archive users will find a directory Data with two subdirectories. Further information about the resource may be found in the file readme.txt which is located in the Data directory. A public GitHub repository which contains all data and code under a GPL v3 license can be accessed by following the link https://github.com/TW-Robotics/NT_BodyParts. (ZIP) [file pone.0249593.s003.zip › Data/images/Hawassa15.jpg]

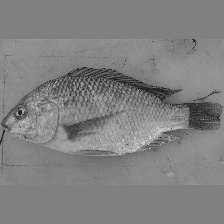

Supplement: S1 File — To allow reproducing the results in this paper we provide all data in a zip archive. After expanding the archive users will find a directory Data with two subdirectories. Further information about the resource may be found in the file readme.txt which is located in the Data directory. A public GitHub repository which contains all data and code under a GPL v3 license can be accessed by following the link https://github.com/TW-Robotics/NT_BodyParts. (ZIP) [file pone.0249593.s003.zip › Data/images/Ziway13.jpg]

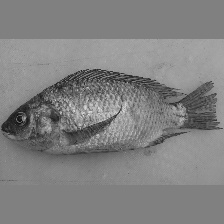

Supplement: S1 File — To allow reproducing the results in this paper we provide all data in a zip archive. After expanding the archive users will find a directory Data with two subdirectories. Further information about the resource may be found in the file readme.txt which is located in the Data directory. A public GitHub repository which contains all data and code under a GPL v3 license can be accessed by following the link https://github.com/TW-Robotics/NT_BodyParts. (ZIP) [file pone.0249593.s003.zip › Data/images/Tana07.jpg]

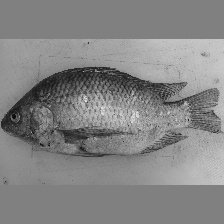

Supplement: S1 File — To allow reproducing the results in this paper we provide all data in a zip archive. After expanding the archive users will find a directory Data with two subdirectories. Further information about the resource may be found in the file readme.txt which is located in the Data directory. A public GitHub repository which contains all data and code under a GPL v3 license can be accessed by following the link https://github.com/TW-Robotics/NT_BodyParts. (ZIP) [file pone.0249593.s003.zip › Data/images/Koka13.jpg]

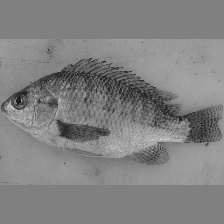

Supplement: S1 File — To allow reproducing the results in this paper we provide all data in a zip archive. After expanding the archive users will find a directory Data with two subdirectories. Further information about the resource may be found in the file readme.txt which is located in the Data directory. A public GitHub repository which contains all data and code under a GPL v3 license can be accessed by following the link https://github.com/TW-Robotics/NT_BodyParts. (ZIP) [file pone.0249593.s003.zip › Data/images/Hawassa29.jpg]

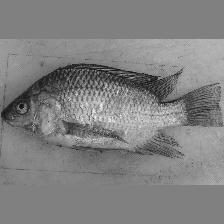

Supplement: S1 File — To allow reproducing the results in this paper we provide all data in a zip archive. After expanding the archive users will find a directory Data with two subdirectories. Further information about the resource may be found in the file readme.txt which is located in the Data directory. A public GitHub repository which contains all data and code under a GPL v3 license can be accessed by following the link https://github.com/TW-Robotics/NT_BodyParts. (ZIP) [file pone.0249593.s003.zip › Data/images/Koka18.jpg]

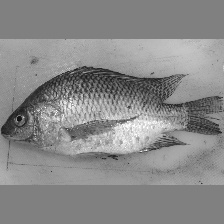

Supplement: S1 File — To allow reproducing the results in this paper we provide all data in a zip archive. After expanding the archive users will find a directory Data with two subdirectories. Further information about the resource may be found in the file readme.txt which is located in the Data directory. A public GitHub repository which contains all data and code under a GPL v3 license can be accessed by following the link https://github.com/TW-Robotics/NT_BodyParts. (ZIP) [file pone.0249593.s003.zip › Data/images/Koka30.jpg]

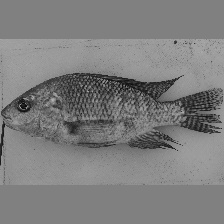

Supplement: S1 File — To allow reproducing the results in this paper we provide all data in a zip archive. After expanding the archive users will find a directory Data with two subdirectories. Further information about the resource may be found in the file readme.txt which is located in the Data directory. A public GitHub repository which contains all data and code under a GPL v3 license can be accessed by following the link https://github.com/TW-Robotics/NT_BodyParts. (ZIP) [file pone.0249593.s003.zip › Data/images/Langano18.jpg]

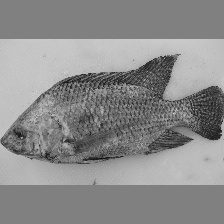

Supplement: S1 File — To allow reproducing the results in this paper we provide all data in a zip archive. After expanding the archive users will find a directory Data with two subdirectories. Further information about the resource may be found in the file readme.txt which is located in the Data directory. A public GitHub repository which contains all data and code under a GPL v3 license can be accessed by following the link https://github.com/TW-Robotics/NT_BodyParts. (ZIP) [file pone.0249593.s003.zip › Data/images/Chamo8.jpg]

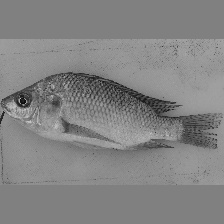

Supplement: S1 File — To allow reproducing the results in this paper we provide all data in a zip archive. After expanding the archive users will find a directory Data with two subdirectories. Further information about the resource may be found in the file readme.txt which is located in the Data directory. A public GitHub repository which contains all data and code under a GPL v3 license can be accessed by following the link https://github.com/TW-Robotics/NT_BodyParts. (ZIP) [file pone.0249593.s003.zip › Data/images/Langano04.jpg]

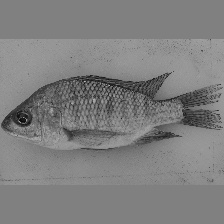

Supplement: S1 File — To allow reproducing the results in this paper we provide all data in a zip archive. After expanding the archive users will find a directory Data with two subdirectories. Further information about the resource may be found in the file readme.txt which is located in the Data directory. A public GitHub repository which contains all data and code under a GPL v3 license can be accessed by following the link https://github.com/TW-Robotics/NT_BodyParts. (ZIP) [file pone.0249593.s003.zip › Data/images/Langano16.jpg]

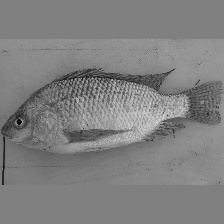

Supplement: S1 File — To allow reproducing the results in this paper we provide all data in a zip archive. After expanding the archive users will find a directory Data with two subdirectories. Further information about the resource may be found in the file readme.txt which is located in the Data directory. A public GitHub repository which contains all data and code under a GPL v3 license can be accessed by following the link https://github.com/TW-Robotics/NT_BodyParts. (ZIP) [file pone.0249593.s003.zip › Data/images/Ziway07.jpg]

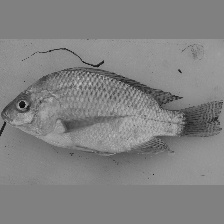

Supplement: S1 File — To allow reproducing the results in this paper we provide all data in a zip archive. After expanding the archive users will find a directory Data with two subdirectories. Further information about the resource may be found in the file readme.txt which is located in the Data directory. A public GitHub repository which contains all data and code under a GPL v3 license can be accessed by following the link https://github.com/TW-Robotics/NT_BodyParts. (ZIP) [file pone.0249593.s003.zip › Data/images/Ziway38.jpg]

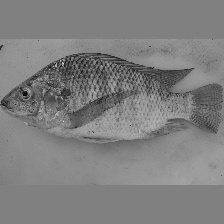

Supplement: S1 File — To allow reproducing the results in this paper we provide all data in a zip archive. After expanding the archive users will find a directory Data with two subdirectories. Further information about the resource may be found in the file readme.txt which is located in the Data directory. A public GitHub repository which contains all data and code under a GPL v3 license can be accessed by following the link https://github.com/TW-Robotics/NT_BodyParts. (ZIP) [file pone.0249593.s003.zip › Data/images/Chamo28.jpg]

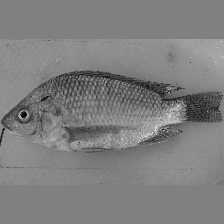

Supplement: S1 File — To allow reproducing the results in this paper we provide all data in a zip archive. After expanding the archive users will find a directory Data with two subdirectories. Further information about the resource may be found in the file readme.txt which is located in the Data directory. A public GitHub repository which contains all data and code under a GPL v3 license can be accessed by following the link https://github.com/TW-Robotics/NT_BodyParts. (ZIP) [file pone.0249593.s003.zip › Data/images/Ziway31.jpg]

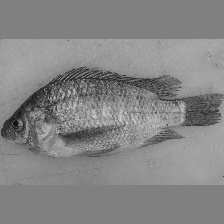

Supplement: S1 File — To allow reproducing the results in this paper we provide all data in a zip archive. After expanding the archive users will find a directory Data with two subdirectories. Further information about the resource may be found in the file readme.txt which is located in the Data directory. A public GitHub repository which contains all data and code under a GPL v3 license can be accessed by following the link https://github.com/TW-Robotics/NT_BodyParts. (ZIP) [file pone.0249593.s003.zip › Data/images/Tana13.jpg]

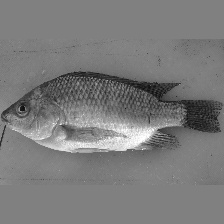

Supplement: S1 File — To allow reproducing the results in this paper we provide all data in a zip archive. After expanding the archive users will find a directory Data with two subdirectories. Further information about the resource may be found in the file readme.txt which is located in the Data directory. A public GitHub repository which contains all data and code under a GPL v3 license can be accessed by following the link https://github.com/TW-Robotics/NT_BodyParts. (ZIP) [file pone.0249593.s003.zip › Data/images/Ziway32.jpg]

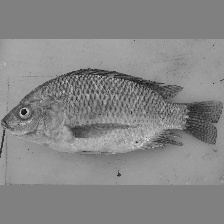

Supplement: S1 File — To allow reproducing the results in this paper we provide all data in a zip archive. After expanding the archive users will find a directory Data with two subdirectories. Further information about the resource may be found in the file readme.txt which is located in the Data directory. A public GitHub repository which contains all data and code under a GPL v3 license can be accessed by following the link https://github.com/TW-Robotics/NT_BodyParts. (ZIP) [file pone.0249593.s003.zip › Data/images/Ziway21.jpg]

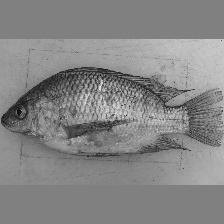

Supplement: S1 File — To allow reproducing the results in this paper we provide all data in a zip archive. After expanding the archive users will find a directory Data with two subdirectories. Further information about the resource may be found in the file readme.txt which is located in the Data directory. A public GitHub repository which contains all data and code under a GPL v3 license can be accessed by following the link https://github.com/TW-Robotics/NT_BodyParts. (ZIP) [file pone.0249593.s003.zip › Data/images/Koka06.jpg]

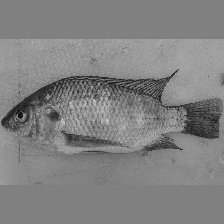

Supplement: S1 File — To allow reproducing the results in this paper we provide all data in a zip archive. After expanding the archive users will find a directory Data with two subdirectories. Further information about the resource may be found in the file readme.txt which is located in the Data directory. A public GitHub repository which contains all data and code under a GPL v3 license can be accessed by following the link https://github.com/TW-Robotics/NT_BodyParts. (ZIP) [file pone.0249593.s003.zip › Data/images/Tana29.jpg]

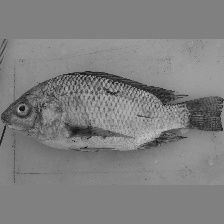

Supplement: S1 File — To allow reproducing the results in this paper we provide all data in a zip archive. After expanding the archive users will find a directory Data with two subdirectories. Further information about the resource may be found in the file readme.txt which is located in the Data directory. A public GitHub repository which contains all data and code under a GPL v3 license can be accessed by following the link https://github.com/TW-Robotics/NT_BodyParts. (ZIP) [file pone.0249593.s003.zip › Data/images/Ziway09.jpg]

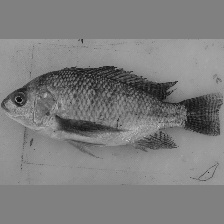

Supplement: S1 File — To allow reproducing the results in this paper we provide all data in a zip archive. After expanding the archive users will find a directory Data with two subdirectories. Further information about the resource may be found in the file readme.txt which is located in the Data directory. A public GitHub repository which contains all data and code under a GPL v3 license can be accessed by following the link https://github.com/TW-Robotics/NT_BodyParts. (ZIP) [file pone.0249593.s003.zip › Data/images/Hawassa26.jpg]

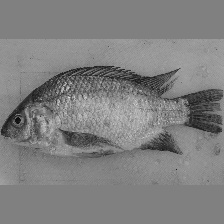

Supplement: S1 File — To allow reproducing the results in this paper we provide all data in a zip archive. After expanding the archive users will find a directory Data with two subdirectories. Further information about the resource may be found in the file readme.txt which is located in the Data directory. A public GitHub repository which contains all data and code under a GPL v3 license can be accessed by following the link https://github.com/TW-Robotics/NT_BodyParts. (ZIP) [file pone.0249593.s003.zip › Data/images/Tana33.jpg]

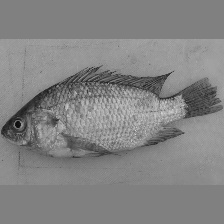

Supplement: S1 File — To allow reproducing the results in this paper we provide all data in a zip archive. After expanding the archive users will find a directory Data with two subdirectories. Further information about the resource may be found in the file readme.txt which is located in the Data directory. A public GitHub repository which contains all data and code under a GPL v3 license can be accessed by following the link https://github.com/TW-Robotics/NT_BodyParts. (ZIP) [file pone.0249593.s003.zip › Data/images/Tana09.jpg]

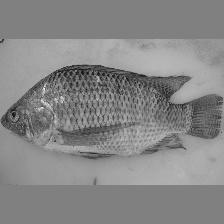

Supplement: S1 File — To allow reproducing the results in this paper we provide all data in a zip archive. After expanding the archive users will find a directory Data with two subdirectories. Further information about the resource may be found in the file readme.txt which is located in the Data directory. A public GitHub repository which contains all data and code under a GPL v3 license can be accessed by following the link https://github.com/TW-Robotics/NT_BodyParts. (ZIP) [file pone.0249593.s003.zip › Data/images/Chamo31.jpg]

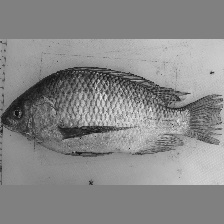

Supplement: S1 File — To allow reproducing the results in this paper we provide all data in a zip archive. After expanding the archive users will find a directory Data with two subdirectories. Further information about the resource may be found in the file readme.txt which is located in the Data directory. A public GitHub repository which contains all data and code under a GPL v3 license can be accessed by following the link https://github.com/TW-Robotics/NT_BodyParts. (ZIP) [file pone.0249593.s003.zip › Data/images/Koka08.jpg]

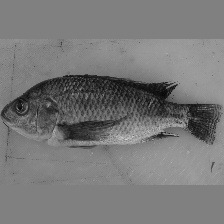

Supplement: S1 File — To allow reproducing the results in this paper we provide all data in a zip archive. After expanding the archive users will find a directory Data with two subdirectories. Further information about the resource may be found in the file readme.txt which is located in the Data directory. A public GitHub repository which contains all data and code under a GPL v3 license can be accessed by following the link https://github.com/TW-Robotics/NT_BodyParts. (ZIP) [file pone.0249593.s003.zip › Data/images/Hawassa36.jpg]

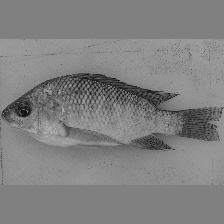

Supplement: S1 File — To allow reproducing the results in this paper we provide all data in a zip archive. After expanding the archive users will find a directory Data with two subdirectories. Further information about the resource may be found in the file readme.txt which is located in the Data directory. A public GitHub repository which contains all data and code under a GPL v3 license can be accessed by following the link https://github.com/TW-Robotics/NT_BodyParts. (ZIP) [file pone.0249593.s003.zip › Data/images/Langano12.jpg]

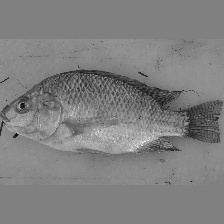

Supplement: S1 File — To allow reproducing the results in this paper we provide all data in a zip archive. After expanding the archive users will find a directory Data with two subdirectories. Further information about the resource may be found in the file readme.txt which is located in the Data directory. A public GitHub repository which contains all data and code under a GPL v3 license can be accessed by following the link https://github.com/TW-Robotics/NT_BodyParts. (ZIP) [file pone.0249593.s003.zip › Data/images/Ziway18.jpg]

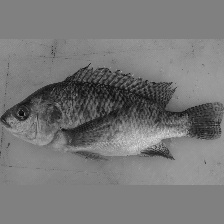

Supplement: S1 File — To allow reproducing the results in this paper we provide all data in a zip archive. After expanding the archive users will find a directory Data with two subdirectories. Further information about the resource may be found in the file readme.txt which is located in the Data directory. A public GitHub repository which contains all data and code under a GPL v3 license can be accessed by following the link https://github.com/TW-Robotics/NT_BodyParts. (ZIP) [file pone.0249593.s003.zip › Data/images/Hawassa30.jpg]

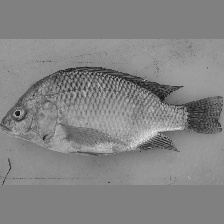

Supplement: S1 File — To allow reproducing the results in this paper we provide all data in a zip archive. After expanding the archive users will find a directory Data with two subdirectories. Further information about the resource may be found in the file readme.txt which is located in the Data directory. A public GitHub repository which contains all data and code under a GPL v3 license can be accessed by following the link https://github.com/TW-Robotics/NT_BodyParts. (ZIP) [file pone.0249593.s003.zip › Data/images/Ziway12.jpg]

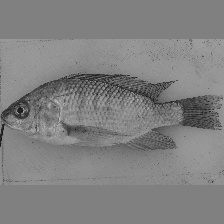

Supplement: S1 File — To allow reproducing the results in this paper we provide all data in a zip archive. After expanding the archive users will find a directory Data with two subdirectories. Further information about the resource may be found in the file readme.txt which is located in the Data directory. A public GitHub repository which contains all data and code under a GPL v3 license can be accessed by following the link https://github.com/TW-Robotics/NT_BodyParts. (ZIP) [file pone.0249593.s003.zip › Data/images/Langano17.jpg]

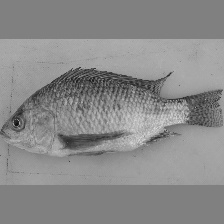

Supplement: S1 File — To allow reproducing the results in this paper we provide all data in a zip archive. After expanding the archive users will find a directory Data with two subdirectories. Further information about the resource may be found in the file readme.txt which is located in the Data directory. A public GitHub repository which contains all data and code under a GPL v3 license can be accessed by following the link https://github.com/TW-Robotics/NT_BodyParts. (ZIP) [file pone.0249593.s003.zip › Data/images/Hawassa12.jpg]

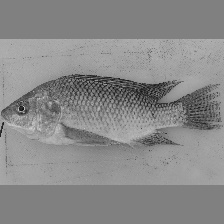

Supplement: S1 File — To allow reproducing the results in this paper we provide all data in a zip archive. After expanding the archive users will find a directory Data with two subdirectories. Further information about the resource may be found in the file readme.txt which is located in the Data directory. A public GitHub repository which contains all data and code under a GPL v3 license can be accessed by following the link https://github.com/TW-Robotics/NT_BodyParts. (ZIP) [file pone.0249593.s003.zip › Data/images/Langano03.jpg]

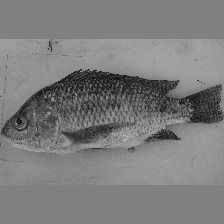

Supplement: S1 File — To allow reproducing the results in this paper we provide all data in a zip archive. After expanding the archive users will find a directory Data with two subdirectories. Further information about the resource may be found in the file readme.txt which is located in the Data directory. A public GitHub repository which contains all data and code under a GPL v3 license can be accessed by following the link https://github.com/TW-Robotics/NT_BodyParts. (ZIP) [file pone.0249593.s003.zip › Data/images/Hawassa21.jpg]

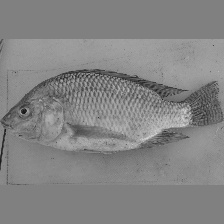

Supplement: S1 File — To allow reproducing the results in this paper we provide all data in a zip archive. After expanding the archive users will find a directory Data with two subdirectories. Further information about the resource may be found in the file readme.txt which is located in the Data directory. A public GitHub repository which contains all data and code under a GPL v3 license can be accessed by following the link https://github.com/TW-Robotics/NT_BodyParts. (ZIP) [file pone.0249593.s003.zip › Data/images/Ziway04.jpg]

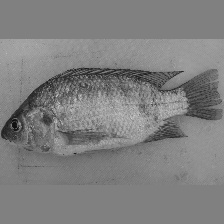

Supplement: S1 File — To allow reproducing the results in this paper we provide all data in a zip archive. After expanding the archive users will find a directory Data with two subdirectories. Further information about the resource may be found in the file readme.txt which is located in the Data directory. A public GitHub repository which contains all data and code under a GPL v3 license can be accessed by following the link https://github.com/TW-Robotics/NT_BodyParts. (ZIP) [file pone.0249593.s003.zip › Data/images/Tana19.jpg]

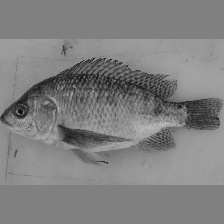

Supplement: S1 File — To allow reproducing the results in this paper we provide all data in a zip archive. After expanding the archive users will find a directory Data with two subdirectories. Further information about the resource may be found in the file readme.txt which is located in the Data directory. A public GitHub repository which contains all data and code under a GPL v3 license can be accessed by following the link https://github.com/TW-Robotics/NT_BodyParts. (ZIP) [file pone.0249593.s003.zip › Data/images/Hawassa27.jpg]

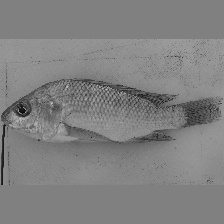

Supplement: S1 File — To allow reproducing the results in this paper we provide all data in a zip archive. After expanding the archive users will find a directory Data with two subdirectories. Further information about the resource may be found in the file readme.txt which is located in the Data directory. A public GitHub repository which contains all data and code under a GPL v3 license can be accessed by following the link https://github.com/TW-Robotics/NT_BodyParts. (ZIP) [file pone.0249593.s003.zip › Data/images/Langano05.jpg]
